# A Programmed DNA Dynamic Assembly‐Guided Molecular Amplifier for Authentic Information Decryption

**DOI:** 10.1002/advs.202409586

**Published:** 2025-05-19

**Authors:** Ning Yang, Jie Zhou, Fengying Yuan, Yajuan Liu, Jia Zhang, Ying Zhuo, Yu Ouyang, Yaqin Chai, Pu Zhang, Ruo Yuan

**Affiliations:** ^1^ Key Laboratory of Luminescence Analysis and Molecular Sensing (Southwest University) Ministry of Education Chongqing Engineering Laboratory of Nanomaterials & Sensor Technologies College of Chemistry and Chemical Engineering Southwest University Chongqing 400715 P. R. China

**Keywords:** DNA self‐assembly, DNA decryption, DNA sensing, DNA nanotechnology, nanomachine

## Abstract

Nucleic acid‐based cryptographic approaches are an innovative emerging field for information process. However, the poor reproducibility and interference from bioenvironment of the existing decryption led to different binary translation according to the fixed threshold defined by “Sender”, which seriously affects the authenticity during message communication. Here, a programmed DNA constitutional dynamic network (CDN)‐derived adaptive threshold is shown, which is defined by the difference value of the two groups of the output patterns from CDN. Under external stimuli, the threshold is adaptive to the generated dynamic output patterns, which avoids the contrary binary translation from a slight difference on the output under fixed threshold. Importantly, there are two self‐calibrating patterns in each output group and the total concentration of constituents from the CDN system are constant, which greatly eliminates the data error. The CDN system is accompanied by computational simulation, which can predict the output patterns of the system at different states. The CDN is used to control the orthogonal and cascaded nanoparticle‐based molecular amplifiers to expand the volume of the transmitting message, as well as allow the accurate and specific sensing of DNA. Various state‐of‐the‐art representation is demonstrated by coding and decoding different types of messages.

## Graphical abstract

This study presents a DNA‐based constitutional dynamic network (CDN) with adaptive thresholds for secure information decryption. The system self‐calibrates output patterns, ensuring reproducibility and accuracy, and integrates molecular amplifiers for expanded message capacity and precise DNA sensing.

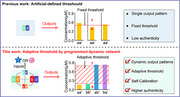

## 1 Introduction

In the information era, a large amount of sensitive information is exchanged through public communications or computer networks, and the security of transmitting sensitive information has become a thriving and improving discipline.^[^ ^1^ ^]^ Sophisticated cryptography serves as the foundational technical framework to ensure the security, authenticity, and integrity of information during transmission.^[^ ^2^ ^]^ Electronic computers and the new emerging quantum computers are two streams of popular cryptography means, while these are allowed to be disturbed under electronic viruses or brute force attacks.^[^ ^3^ ^]^ Besides, the semiconductor‐based technology shows a limited lifespan, large space consumption and poor environmental stability compared to that of the biotechnology‐dependent deciphering tools.^[^ ^4^ ^]^

**Figure 1:**
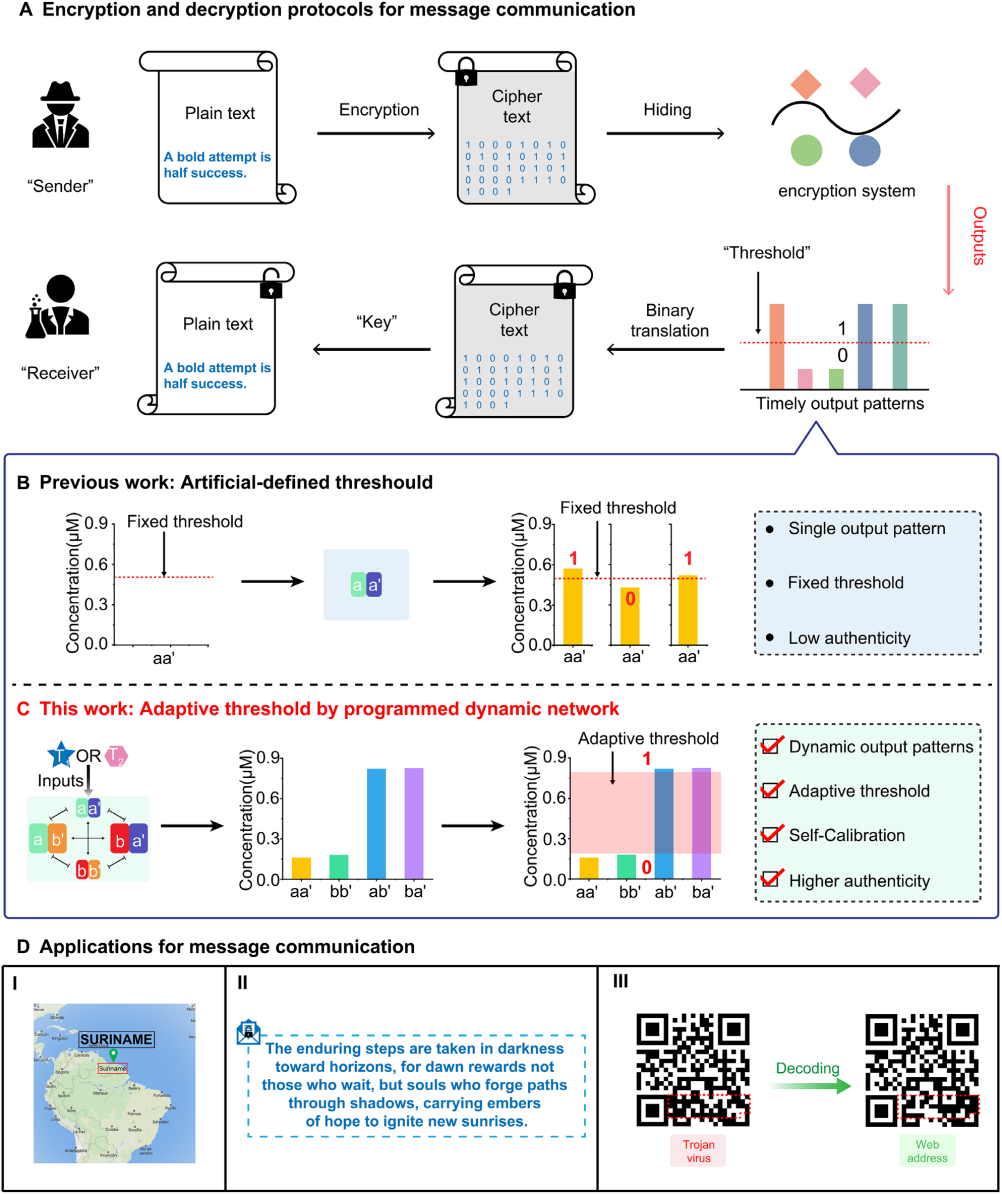
Comparison of the threshold between the traditional artificial predefined system and the adaptive, systematic defined CDN system. A) The encryption and decryption protocols based on the timely output patterns. B) Traditional system generated from aa' predefined with a fixed threshold and the corresponding output patterns. C) The CDN system generating output pattern defined by an adaptive threshold under the external stimuli of DNA, T_1_, or T_2_. The CDN‐based system shows an adaptive threshold than that of the artificial predefinition conducted by a dependent aa'‐based signal report, which may intensively improve the authenticity of the decryption process. D) Schematic demonstration of diverse contemporary representations through the encoding and decoding of various message types, including geographical location data (I), textual information (II), and Quick Response (QR) codes (III). This illustration highlights the versatility and applicability of the encoding‐decoding framework across multiple data formats.

Deoxyribonucleic acid, DNA, is one of the most important basic molecules of life system, which constitute the genetic instructions to guide biological heredity, various life functions, and subcellular biofeatures.^[^ ^5^ ^]^ The base sequences, A, T, G, and C, encoded in DNA programs various functional and structural regulating information by Watson‐Crick base‐paring through hydrogen bonds.^[^ ^6^ ^]^ Compared to traditional mathematics‐based cryptography, DNA‐based encryption and decryption technology provides high security since it depends not only on the mathematical theory but also on the biotechnology that the DNA molecules possess native complexity and randomness.^[^ ^7^ ^]^ And it shows high information storage capacity and long‐term preservation of the information, making it a promising candidate for modern information processes.^[^ ^8^ ^]^ Besides, highly programmed functional features of DNA provide a reservoir of tools to use DNA as functional, controllable material for information cryptography.^[^ ^9^ ^]^ Encryption and decryption of sensitive information by DNA technology usually undergo the following steps, **Figure** **1**A.^[^ ^10^ ^]^ The “Sender” constructs a binary coded array that is used as the cipher book to decipher the output patterns into “0/1” digits. The “Receiver” subjects “inputs” stimuli to encryption device to get a “0/1” binary code. The binary text is then translated to plain text under the instruction of the cipher book. The “threshold” determination is highly important to decipher the output pattern since it decides the binary translation. That is, different ranges of the “threshold” may translate the output patterns into reverse binary‐coded digits, “0” into “1” or “1” into “0”, which could intensively disturb the authenticity of the transmitting message. To date, a variety of DNA‐based strategies have been utilized for information cryptography, including nucleic acid sequencing,^[^ ^11^ ^]^ visible microscopies,^[^ ^10^, ^12^ ^]^ as well as timely optical readout response.^[^ ^13^ ^]^ For example, the secret information is encoded into a DNA‐based, doubly steganographic microdot,^[^ ^14^ ^]^ and the secret message is concealed in microdots containing 100 copies of DNA and is deciphered by sequencing the PCR amplified DNA product. Fan's group developed DNA origami‐based information encryption that an M13 viral scaffold is self‐assembled into a braille‐like nano‐pattern for secure communication.^[^ ^10^ ^]^ While sequencing‐based methodologies are inherently associated with a non‐negligible error rate, which can result in nucleotide misidentification and indel (insertion/deletion) errors, particularly within repetitive genomic regions. Therefore, the sequencing‐based decryption methodology is fundamentally dependent on the statistical analysis of a substantial volume of sequencing results. The origami‐based methods ask for precise calculation and optimization, demanding specialized knowledge and experience from the designer. Furthermore, the associated data analysis utilizing atomic force microscopy (AFM), which lacks quantitative capability, is prone to the observation of incomplete reaction products, consequently exacerbating the decryption error rate. Timely optical readout response may be considered as an ideal method for message decryption. For example, Gao's group reported programmable DNA motifs that are coordinately regulated by enthalpy and entropy for information encryption.^[^ ^15^ ^]^ Through the above encryption methods, DNA,^[^ ^16^ ^]^ as well as related biomolecules, e.g. protein,^[^ ^17^ ^]^ amino acids,^[^ ^18^ ^]^ can be programmed to stimulate the sensing systems by inducing the conformational change of the DNA motifs and thus may provide a collection of “smart” candidates for information communication. However, in most cases, the binary‐coding digit evaluation, termed as “threshold”, “0” or “1”, of the DNA‐based encryption system is artificially defined, or “predefined”, by the “Sender”, which is presented as a “liner” distribution, as shown in Figure 1B. The fixed threshold may probably be altered by slight difference in the output signal intensity. As stated above, the very minute “threshold” definition may cause information transmitting error under the circumstance that the output pattern shows accident expression. Therefore, a rapid, easy‐to‐decode, and most importantly, highly authentic DNA decryption with a wide “threshold” definition is extremely challenging.

Intermolecular dynamic interactions between DNA ^[^ ^19^ ^]^ and relating biomolecules, e.g., RNA, proteins, small molecule metabolites, and low‐molecular‐weight ligands, form complicated biological networks ^[^ ^20^ ^]^ and underpin various fundamental life processes.^[^ ^21^ ^]^ The constituents in these networks may exchange under environmental physical ^[^ ^22^ ^]^ or chemical stimuli,^[^ ^23^ ^]^ which terms as constitutional dynamic network, CDN.^[^ ^24^ ^]^ The basic model of a DNA‐based CDN system, a [2 × 2] network, consisted of four equilibrated nucleic acid components, C, C', D, D', generating four DNA constituents, CC', CD', DC', DD' under sequence‐complementation. Treated with external stimuli, the antagonistic constituents, CC' and DD', are up‐regulated, while the CD' and DC' are down‐regulated. These dynamic, equilibrated features of DNA‐based CDN systems enable the stimuli‐responsive, multi‐signal transduction, up‐regulated or down‐regulated constituents in the dynamic system. Especially, the unique regulation of dynamic signal output may provide an adaptive, combinatorial threshold definition that is generated from the network system, instead of an artificial predetermination during the output pattern transferred to binary digits process, as shown in Figure 1C. It should be noted that, few researches have utilized the CDN systems for information process by using the dynamic component exchanging or selection of different organic molecules instead of nucleic acid constituents.^[^ ^25^ ^]^ These researches realize the information process via reconfiguration of networks by regulating the aggregation of small organic molecules, while the programmability of these small organic molecules is not comparable to the nucleic acid‐based CDN. For example, the DNA‐based systems are possible to be responsive to nucleic acids,^[^ ^26^ ^]^ proteins,^[^ ^27^ ^]^ small molecules ^[^ ^28^ ^]^ under reasonable, precise programmability. Also, it can be triggered by light ^[^ ^22^ ^]^ or chemical reagents ^[^ ^28^ ^]^ through intercalating corresponding photosensitive small molecules or conjugating potassium ions by stabilizing the G‐quadruplex DNA motif. Besides, applying CDNs as external functional moduli that control the sophisticated, smart, molecular amplifiers ^[^ ^29^ ^]^ is at present unreported. In view of this, we envision that developing a DNA‐based CDN‐guided molecular amplifier system could lead to a dramatic improvement in the authenticity of the information transmission and provide a sophisticated, complicated message encryption platform.

**Figure 2:**
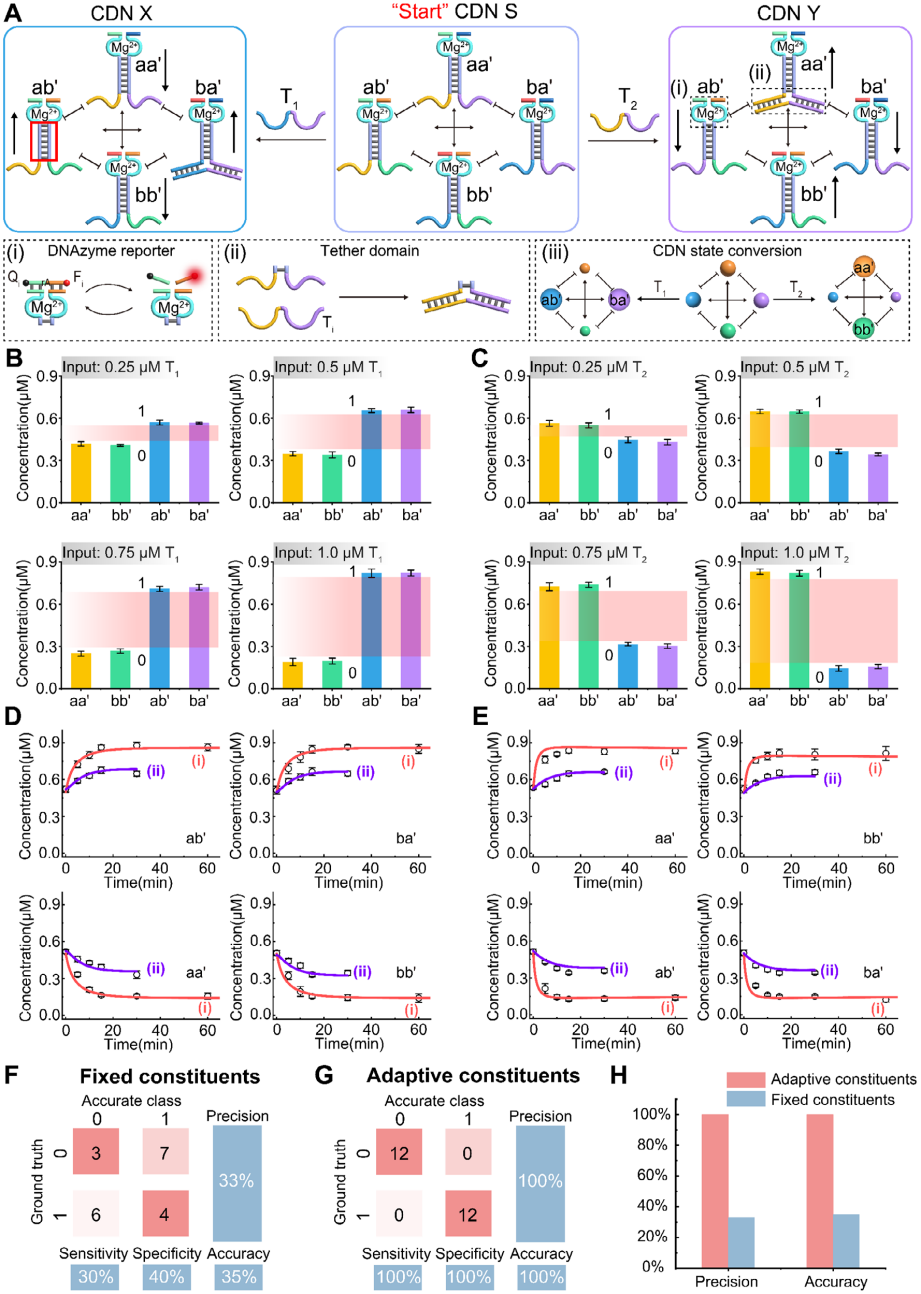
A) Schematic presentation of the trigger‐guided reconfiguration of CDN, aa', bb', ab', and ba'. The concentrations of the constituents are probed by the DNAzyme reporter units associated with the constituents that cleave the respective fluorophore (F_i_)/quencher (Q_i_)‐modified substrates, panel (i). The transformation of the CDN by the respective trigger (T_i_ = T_1_ or T_2_) hybridize to the tether domain associated to corresponding CDN constituents, panel (ii). Schematic adaptive reconfiguration of the [2 × 2] CDN “S” into CDN “X” in the presence of trigger T_1_, and the reconfiguration of CDN “S” into CDN “Y” in the presence of trigger T_2_, panel (iii). B) Content changes generated by the reporter units associated with CDN constituents at different concentrations of T_1_ in the form of a bar presentation. The red area is the adaptive threshold. Error bars represent mean ± SD, *n* = 3. C) Content changes generated by the reporter units associated with CDN constituents at different concentrations of T_2_ in the form of a bar presentation. The red area is the adaptive threshold. Error bars represent mean ± SD, *n* = 3. D) Temporal concentration changes of the constituents upon the (i) 1 µM and (ii) 0.5 µM of T_1_‐triggered reconfiguration of CDN “S” to CDN “X” (Experimentally, point; computationally simulated, solid lines; computationally simulated concentration changes are based on the kinetic model formulated in Figure S9, Supporting Information). Error bars represent mean ± SD, *n* = 3. E) Temporal concentration changes of the constituents upon the 0.5 µM (ii) and 1 µM (i) T_2_‐triggered reconfiguration of CDN “S” to CDN “Y” (Experimentally, point; computationally simulated, solid lines; computationally simulated concentration changes using the kinetic model formulated in Figure S10, Supporting Information). Error bars represent mean ± SD, *n* = 3. F) Confusion matrix analysis evaluating the authenticity of message decryption for systems with fixed constituents. G) Confusion matrix analysis assessing the authenticity of message decryption for systems with adaptive constituents. H) Comparative analysis of precision and accuracy between fixed and adaptive constituent systems, derived from the data presented in Figure F and G. This figure provides a comprehensive evaluation of the performance metrix for both fixed and adaptive constituent configurations in message decryption tasks.

Herein, we present a DNA‐based CDN system, where four components, a, a', b, b', are self‐assembled into four equilibrated constituents, aa', ab', ba', bb', sharing complementary sequences, followed by subjecting trigger strand, T_1_ or T_2_, to reconfigure the network by dynamically exchanging the components from the corresponding constituents, as shown in **Figure** **2**A. It should be noted that, each of the constituents, aa', ab', ba', bb', is engineering programmed with a signal reporter, an E6 deoxy‐ribozyme (DNAzyme), which enables the CDN system to generate four mutual‐dependent, mutual‐verifiable, timely fluorescent readout patterns during the re‐equilibrated process. Figure 1C demonstrates the four output channels corresponding to the four constituents in CDN without a pre‐defined threshold. The combination of the four output signals automatically generates an adaptive threshold that defines the binary‐coded digits, “0” or “1”. As comparison, a non‐dynamic aa“‐based system is artificially defined with a fixed threshold before conducting the output signal. As shown in Figure 1B, the signals would probably show slightly difference by conducting the experiments for few times. While the signals may appear higher or lower value than the threshold, resulting in contrary binary translation during the message decryption process. Comparing the two methods, it can be concluded that the CDN‐based system shows a significantly difference value than that of the artificial predefinition conducted by a dependent aa”‐based signal report, which avoiding message decryption error from different output pattern by accident. Based on the fundamental CDN system, increment on the capacity of binary translation strings in cipher text can indeed lead to enhanced security and increased data representation, which allows for greater precision, larger ranges, or more complex data encoding. To extend the multi‐class application of the nucleic acid CDN‐based system, the dynamic network is used to control the orthogonal and cascaded nanoparticle‐based molecular amplifier, as well as allow the accurate and specific sensing of DNAs through the mutual‐verified multiple output signals. Finally, this system has been successfully applied to encode and decode different types of messages, including location, text, and Quick Response (QR) codes, demonstrating its great potential in the field of information decryption, as shown in Figure 1D.

## 2 Results and Discussion

### 2.1 Constitutional Dynamic Network for Authentic Message Decryption

Figure 2A schematically shows the constitutional dynamic network “S” (CDN “S”) initiating by triggers T_1_ or T_2_ to yield CDN “X” or CDN “Y”, respectively. Four single stranded components, a, a', b, b', are self‐assembled into four equilibrated constituents, aa', ab', ba', bb', sharing complementary sequences. Each of the constituents in different CDNs includes a Mg^2+^‐ion‐dependent E6 DNAzyme as reporter unit, Figure 2A panel (i), and includes a tether domain to recognize the DNA stimuli for the reconfiguration of the dynamic system, Figure 2A panel (ii). The four constituents in CDN “S” are under equilibrium state with the equal concentration. Subjection of trigger strand, T_1_, to CDN “S” stabilizes the constituent ba' by the dissociation of constituents, aa' and bb', thus increasing the content of ab' from the hybridization of the residual components a and b'. As a result, the CDN “S” transfers to CDN “X” by dynamically exchanging the components from the corresponding constituents. The corresponding up‐regulation or down‐regulation of the constituents are marked with arrow. Accordingly, subjection of trigger strand, T_2_, leads to CDN “S” transfers to CDN “Y” by the stabilization of the constituent aa' through the dissociation of ab' and ba', and the accompanying increasing of bb' content. The state conversion of different CDNs under up‐regulation or down‐regulation of respective constituents by different triggers is shown in Figure 2A panel (iii). The engineering design of CDN system by using a NUPACK program is shown in Figure S1 (Supporting Information). The characterization of the formation of each constituent and the reconfiguration of the CDN system by polyacrylamide gel electrophoresis (PAGE) using DNA ladder as the reference is shown in Figure S2 (Supporting Information). It should be noted that a nucleic acid tether is extended from component a and b' to generate a clear separation bands corresponding to the four constituents associated with CDN “S”, “X”, “Y”, which might slightly affect on the equilibrium of the CDN system, see Figure S2B (Supporting Information) and the accompanying discussion. The DNAzyme reporters engineered in each of the constituents, aa', ab', ba', bb', enable the CDN system to generate four mutual‐dependent, coordinate‐verifiable, timely fluorescent readout patterns during the re‐equilibrated process. The primary fluorescent characterization of the conversion between different states of the CDNs by subjecting 1.0 µM T_1_ or T_2_, is shown in Figure S4A (Supporting Information). Based on the appropriate calibration curves from the four fluorescent reporters (Figure S3, Supporting Information), the concentrations of different constituents can be seen in Figure S4B (Supporting Information). A light‐stimulated CDN model is designed by embedding the o‐nitrobenzyl phosphate photoresponsive units (PC linker) into the trigger‐blocked strand to demonstrate the high programmability of the DNA‐based decryption method. The schematic illustration and corresponding experimental discussion are depicted in Figures S6 and S7 (Supporting Information).

After the preliminary verification on the conversions of different states of CDN, we subjected different concentrations of T_1_ to generate CDN “X” characterized by a combinatorial output of four time‐dependent fluorescence signals, see Figure S8 (Supporting Information). According to the appropriate calibration curves (Figure S5, Supporting Information), the concentrations of each constituent in CDN “X” in a bar presentation are shown in Figure 2B. The differential among the four bars automatically generates an adaptive threshold (marked in red), and the four bars is later translated to binary digits based on the “threshold” definition, “0” or “1”. Accordingly, the bar presentation of CDN “Y” under different concentrations of trigger T_2_ is shown in Figure 2C, and the corresponding time‐dependent fluorescence signals are shown in Figure S8 (Supporting Information). Based on the primary chemical reactions among the constituents and external DNA stimuli, we built a kinetic model to computationally simulate the time‐dependent concentration changes of the constituents by DNA stimuli triggering. As shown in Figure 2D,E, subjecting the CDN “S” by 1 µM (i) T_1_ or T_2_ and 0.5 µM (ii) respectively, the kinetic model (solid lines) fits well to the experimental data (dots), demonstrating that the content changes of the dynamic system are predictable at different concentrations of DNA stimuli. As can be seen from the result, the CDN system can complete the reconfiguration from different state within 15 minutes. The rate constants (k) corresponding to the conversions between CDN “S” to “X” or CDN “S” to “Y” simulated from the kinetic models are shown in Tables S1 and S2 (Supporting Information). (The reaction equations Figures S9 and S10 (Supporting Information), and the corresponding time‐dependent fluorescence signals Figures S11 and S12, are shown in Supporting Information, Pages S15‐S19). Kinetic model of the CDN system treated with 0.75 µM of T_1_ is examplified in Figures S13 and S14 (Supporting Information). To elucidate the authenticity of the adaptive threshold‐derived method, we compared the precision and accuracy between an individual‐based DNAzyme and CDN‐derived DNAzyme system. Through two sets of ten parallel tests, we counted the experiments‐derived binary translation (ground truth) and the correct information‐required binary digits (accurate class), the confusion matrix is shown in Figure 2F. The two sets of original experimental data on individual aa' DNAzyme and binary digital translation are depicted in Figures S15 (set 1) and S16 (set 2), (Supporting Information). According to the confusion matrix, it can be concluded that the overall precision and accuracy of the adaptive method is around threefold than that of the individual constituent, Figure 2H. Mistranslation of the message by following the individual aa' constituent is discussed, vide infra. The CDN system treated to foreign nucleic acids triggers, miRNA‐21 and miRNA‐221, demonstrates the good selectivity of the designed system. The results are shown in Figure S17 (Supporting Information) and the accompanying discussion, Supporting Information, page S21.

**Figure 3:**
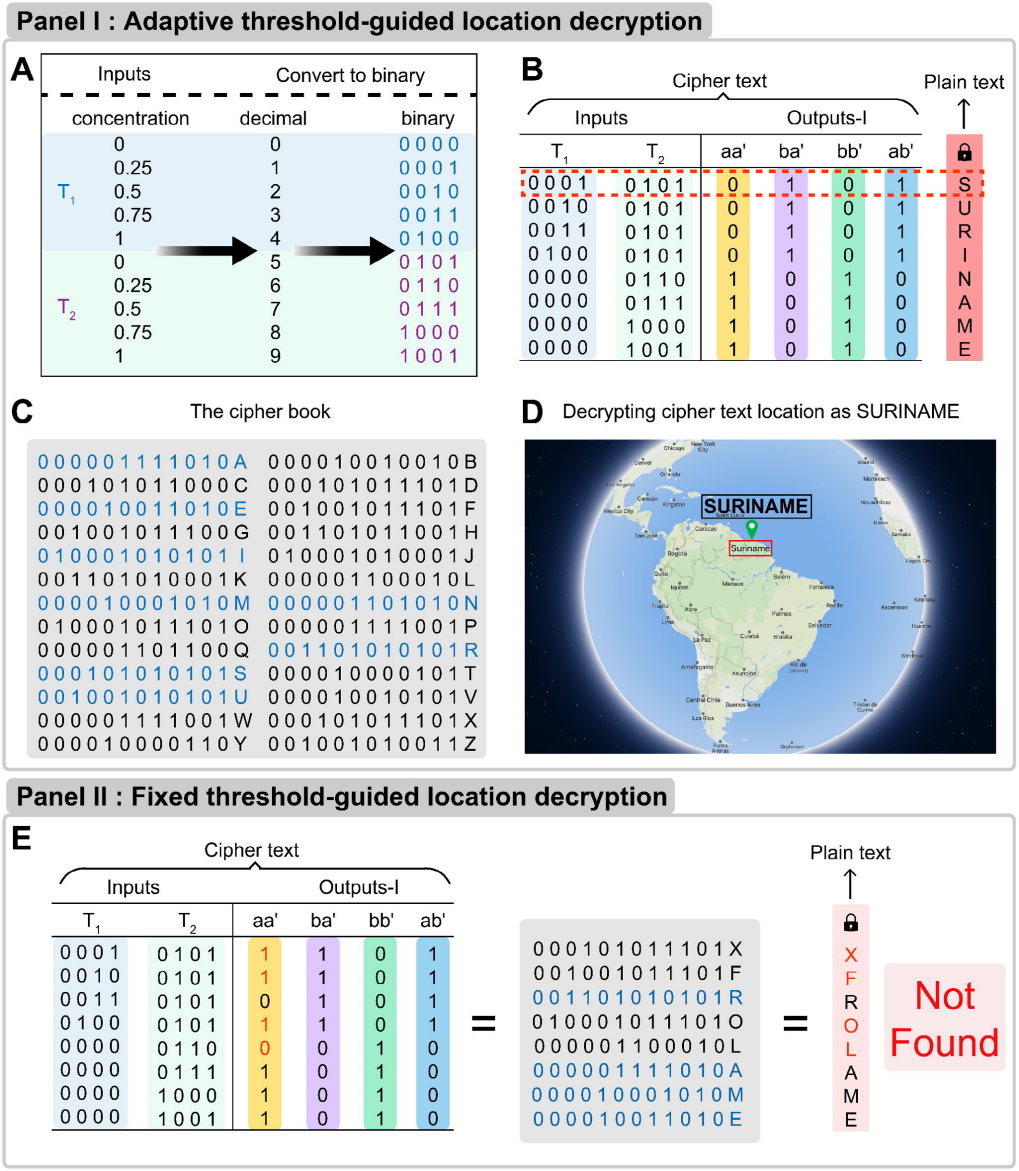
The designed dynamic system is applied to transmit a location message, SURINAME. Panel I: adaptive threshold‐guided location decryption. A) The “input” library given by the “Sender” with specific concentrations of T_1_ and T_2_, and the corresponding decimal and binary digital translation. B) The binary digits library of the inputs and outputs‐I. Specific encryption model of a location, Suriname. C) The cipher book containing the 26‐bit alphabetic code. D) Corresponding decrypted plain text, SURINAME. Panel II: fixed threshold‐guided location decryption. E) The inputs and outputs‐I binary digits by substituting the aa' constituent in the CDN system with an individual aa', and the resulting plain text is decrypted using the cipher book, yielding the incorrect message “XFROLAME”. This panel illustrates the limitations and potential inaccuracies associated with fixed threshold‐based decryption methodologies.

The designed dynamic system is applied to transmit a location message with an eight‐letter text, SURINAME. The plain text of the eight letter is encrypted with cipher text consisting of inputs and outputs‐I, by the “Sender”, into “inputs” list that contains the specific concentrations of triggers (T_1_ and T_2_) with the successional decimal and binary translation (**Figure** **3**, panel I A). The “Receiver” conducted the experiments by subjecting the specific concentrations of T_1_ or T_2_ to observe the output patterns, followed by translating it into binary digits according to the adaptive threshold derived from Figure 2B,C. Combining the binary digits of inputs and outputs‐I, a group of binary digits of 12‐bit, “000 101 010101”, is obtained, as shown in Figure 3, panel I B, line 1. Each of the 12‐bit binary digits represents a letter, e.g., digits in line 1 represents for “S”, which generates a cipher book as shown in Figure 3, panel I C. Based on the cipher text in Figure 3, panel I B and the cipher book as a dictionary (Figure 3, panel I C), the location message, “SURINAME” (Figure 3, panel I D), can be deciphered accurately. As comparison, we substituted the aa' constituent in the CDN system with an individual aa', see Figure 3, panel II E. The aa'‐derived experimental data (shown in Figure S15, Supporting Information) leads to mis‐binary translation, the misleading digits are marked in red. In addition, the stimulation of certain T_1_ and T_2_ indeed limits the capacity of inputs. The capacity of inputs can be expanded by increasing the concentration gradients of T_1_/T_2_, and thus the scalability of the transmitted message can also be expanded. We give an example by triggering the CDN system using T_1_ and T_2_ under different molar ratios, as shown in Figures S18 and S19 (Supporting Information).

The computational cost is a crucial factor when evaluating the algorithms, especially in the context of information encryption and decryption. First, the time complexity is one of the key parameters to investigate. The decryption process for a single character is achieved through the reference to the alphabetical index (cipher book) and the time grows linearly with the input size, O (*n*), where *n* represents the scale of the decryption content (*e.g*., the number of letters). We compared the time complexity of our proposed adaptive method with other DNA‐based algorithms, see Table S3 (Supporting Information). Second, the space complexity is primarily determined by the size of the cipher book utilized in this method, which is constant, i.e., O (*1*). As illustrated in Figure 3C, each character is encoded using twelve binary digits, equivalent to 1.5 bytes. Consequently, the entire cipher book requires a total of 39 bytes of storage. Finally, we evaluate the wall‐clock time of our proposed method. Based on our measurements, a single decryption operation requires ≈15 min to complete. Consequently, within a one‐hour timeframe, our method is capable of decrypting one letter. The comparison of the read out times of this method to other DNA‐based encryption methods is shown in Table S3 (Supporting Information). The conduction time can be principally reduced by shortening the shared domain in each constituent, Figure 2A, marked in red square. The adaptive DNA‐based decryption method has the potential to become an ideal platform in efficient information decryption from computational cost point of view.

### 2.2 Constitutional Dynamic Network‐Guided Orthogonal Molecular Amplifier for Deciphering a Sentence

**Figure 4:**
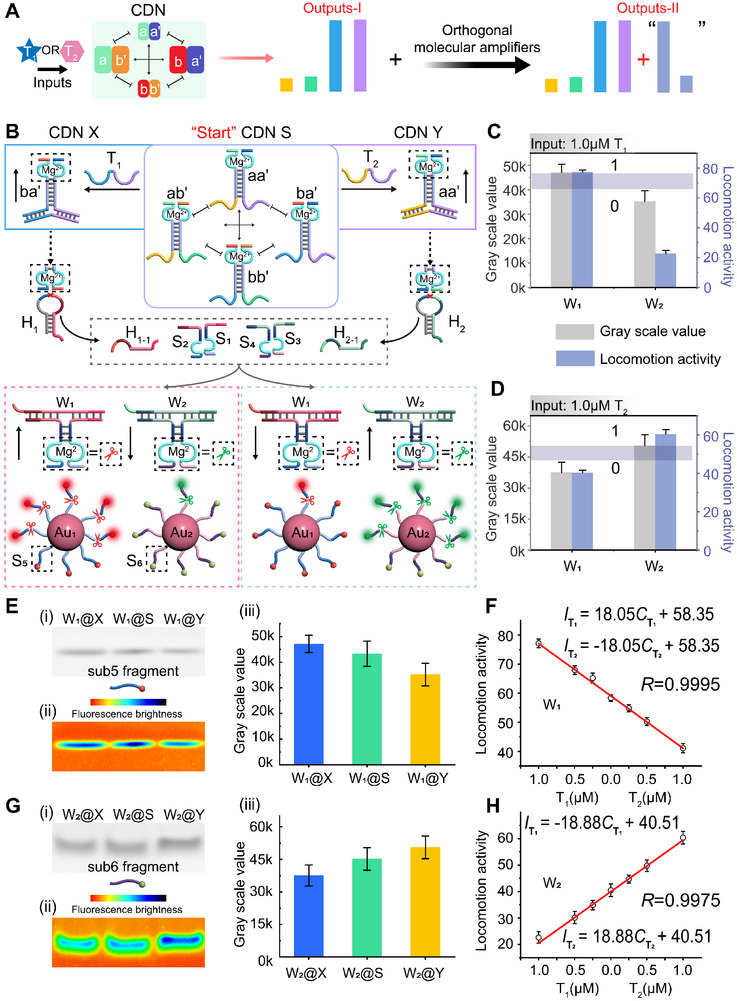
A) Schematic representation of the dynamic adaptive patterns (outputs‐I) derived from the CDN system, and the adaptive patterns (outputs‐II) are expanded by utilizing the CDN system to control over the orthogonal DNA molecular amplifiers. B) The schematic description of the CDN‐guided locomotion of the Au NP‐based molecular amplifier. C) The distributed patterns and the systematic threshold of the T_1_‐treated CDN‐guided walking system from the coordinately expression of gray scale value by the PAGE analysis and the corresponding locomotion activity of the DNA walker, W_1_ and W_2_, from the fluorescent characterization. The blue area is the adaptive threshold. D) The distributed patterns and the systematic threshold of the T_2_‐treated CDN‐guided walking system from the coordinately expression of gray scale value by the PAGE analysis and the corresponding locomotion activity of the DNA walker from the fluorescent characterization. The blue area is the adaptive threshold. E) Representative PAGE analysis showing the locomotion speed of the W_1_ under CDN “S”, “X”, “Y” by cleaving sub5. (i) PAGE images. (ii) Heat maps generated from panel (i). (iii) The corresponding normalized intensity of the bands generated from (i). The cleavage reaction of sub5 is conducted in HEPES buffer (10 mM, pH = 7.2, containing 10 mM MgCl_2_). Error bars represent mean ± SD, *n* = 3. F) The sensing curve of the locomotion activity of the W_1_ (Au_1_‐based molecular amplifier) under different concentrations of T_1_ and T_2_. Error bars represent mean ± SD, *n* = 3. G) Representative PAGE analysis showing the locomotion speed of the W_2_ under CDN “S”, “X”, “Y” by cleaving sub6. (i) PAGE images. (ii) Heat maps generated from panel (i). (iii) The corresponding normalized intensity of the bands generated from (i). The cleavage reactions of sub6 are conducted in HEPES buffer (10 mM, pH = 7.2, containing 10 mM MgCl_2_). Error bars represent mean ± SD, *n* = 3. H) The sensing curve of the locomotion activity (Au_2_‐based molecular amplifier) under different concentrations of T_1_ and T_2_. Error bars represent mean ± SD, *n* = 3.

Realizing that CDN can be applied to build an adaptive threshold‐determined decryption system, we engineered CDN to guide the locomotion of an orthogonal gold nanoparticles (Au NPs)‐based molecular amplifier, to increase the volume of the transmitting message by generating two more “outputs”, outputs‐II in **Figure** **4**A. The decryption system with increased volume is then applied to transfer a “sentence” text, as shown subsequently. The schematic representation of the Au NPs‐based molecular amplifier regulated by the dynamically equilibrated CDN system is shown in Figure 4B. The CDN is subjected with two independent hairpins, H_1_, engineered to be recognized by ba', and H_2_, engineered to be recognized by aa'. H_1_ contains a ribonucleobase‐site, rA, in its loop domain that can be cleaved by ba' to generate a H_1‐1_ fragment. The H_1‐1_ is able to hybridize to two single strands, S_1_ and S_2_, to generate a newly DNAzyme configuration, W_1_, that acting as the walker to cleave the substrate, S_5_, immobilized on the Au NPs 1, Au_1_. (The characterization of the Au NPs by transmission electron microscopy, TEM, shown in Figure S20A‐B (Supporting Information). The successful immobilization of nucleic acids on Au NPs is verified by TEM, UV‐*vis*, Zeta Potential, and agarose gel, see Figures S20C‐F and S21 (Supporting Information). The quantification of S_5_ onto the Au NPs is depicted in Figure S22 (Supporting Information). The related discussion is shown in supporting information, page S26.) The characterization of the CDN system that control over the orthogonal DNA molecular amplifiers by polyacrylamide gel electrophoresis (PAGE) using DNA ladder as the reference is shown in Figure S23 (Supporting Information) and the accompanying discussion. The locomotion of the W_1_ along the interface of Au_1_ is probed by the fluorescence recovery from the TAMRA modified on S_5_. (The TAMRA fluorescence is quenched by Au NPs. Figure S24A (Supporting Information) verified the quenching of TAMRA by Au NPs, and the quenching effect of TAMRA by Au NPs is shown in Figure S24B, Supporting Information). The CDN recognizes hairpin H_2_ to control the second orthogonal molecular amplifier by following the same mechanism. The locomotion of the W_2_ along the interface of Au_2_ is probed by the fluorescence recovery from the FAM modified on S_6_, the corresponding verification of the quenching effect is shown in Figure S24C,D (Supporting Information). (The quantification of S_6_ onto the Au NPs based on the corresponding calibration curves of S_6_ is depicted in Figure S22C,D, Supporting Information). It should be noted that in the absence of the fragmented H_1‐1_ and H_2‐1_, the two walkers, W_1_ composed of S_1_ and S_2_, W_2_ composed of S_3_ and S_4_, cannot “cut” the corresponding substrates on the Au NPs, the preliminary fluorescence verification is shown in Figure S25 (Supporting Information). That is to say, the locomotion of the two molecular amplifiers is regulated by the CDN under the subjection of different triggers by the connection of two hairpins. We use the homogeneous substrate (sub5/6) that modified with a fluorophore and a corresponding quencher on the two ends to verify the primary regulation of each hairpin by the CDN system, the results are shown in Figure S26 (Supporting Information).

After the preliminary experiments to verify the concept, we use CDN to regulate the walker strand to move along the substrates modified on the Au NPs. The fragmented sub5 from different state of CDN is characterized by the PAGE analysis. The intensity of the gel band represents the amounts of the digested sub5, which demonstrates the locomotion speed of the corresponding walkers. As shown in Figure 4E(i), the band of sub5 fragments from CDN “X” (W_1_@X), under the treatment of 1 µM T_1_, is stronger than that from CDN “S” since the stabilization of ba' constituents and the concomitant increasing of W_1_. While the amount of sub5 fragments is weak from CDN “Y” (W_1_@Y) compared to that from CDN “S” (W_1_@S). The corresponding heat map representation and the gray scale value of the fragmented sub5 generated from Figure 4E(i) is shown in Figure 4E(ii),(iii). Using the CDN‐guided molecular amplifier to sense different concentrations of T_1_ and T_2_ by probing the recovered fluorescence of TAMRA (S_5_) is shown in Figure 4F. We use the slope of the time‐dependent fluorescence recovery of TAMRA (S_5_) released from Au NPs (from Figure S27A, Supporting Information) to represent the locomotion activity of the walker strand “W_1_”. As can be observed, the slope of fluorescence from Figure S27A (Supporting Information) gradually decreased accompanying the decreasing subjection of T_1_ and the increasing subjection of T_2_. The antagonistic, two categories of output patterns, including fluorescent response of cleavage rates of the two walkers and the gray scale value of the fragments from W_1_ and W_2_, corporately generate an “adaptive threshold” that defines the binary translation associated to each category of output pattern, as shown in Figure 4C. Accordingly, subjecting T_2_ to the CDN system leads to the more products of sub6 fragments associated to W_2_ than that of the T_1_‐treated CDN system. The corresponding bands of sub6 from different states of CDN and the fluorescence locomotion activity associated to the W_2_ is shown in Figure 4G,H. The corresponding heat map representation and the gray scale value of the fragmented sub6 generated from Figure 4G(i) is shown in Figure 4G(ii),(iii). The time‐dependent fluorescent response correlated to Figure 4H is shown in Figure S27B. The adaptive threshold based on Figure 4G,H is shown in Figure 4D.

**Figure 5:**
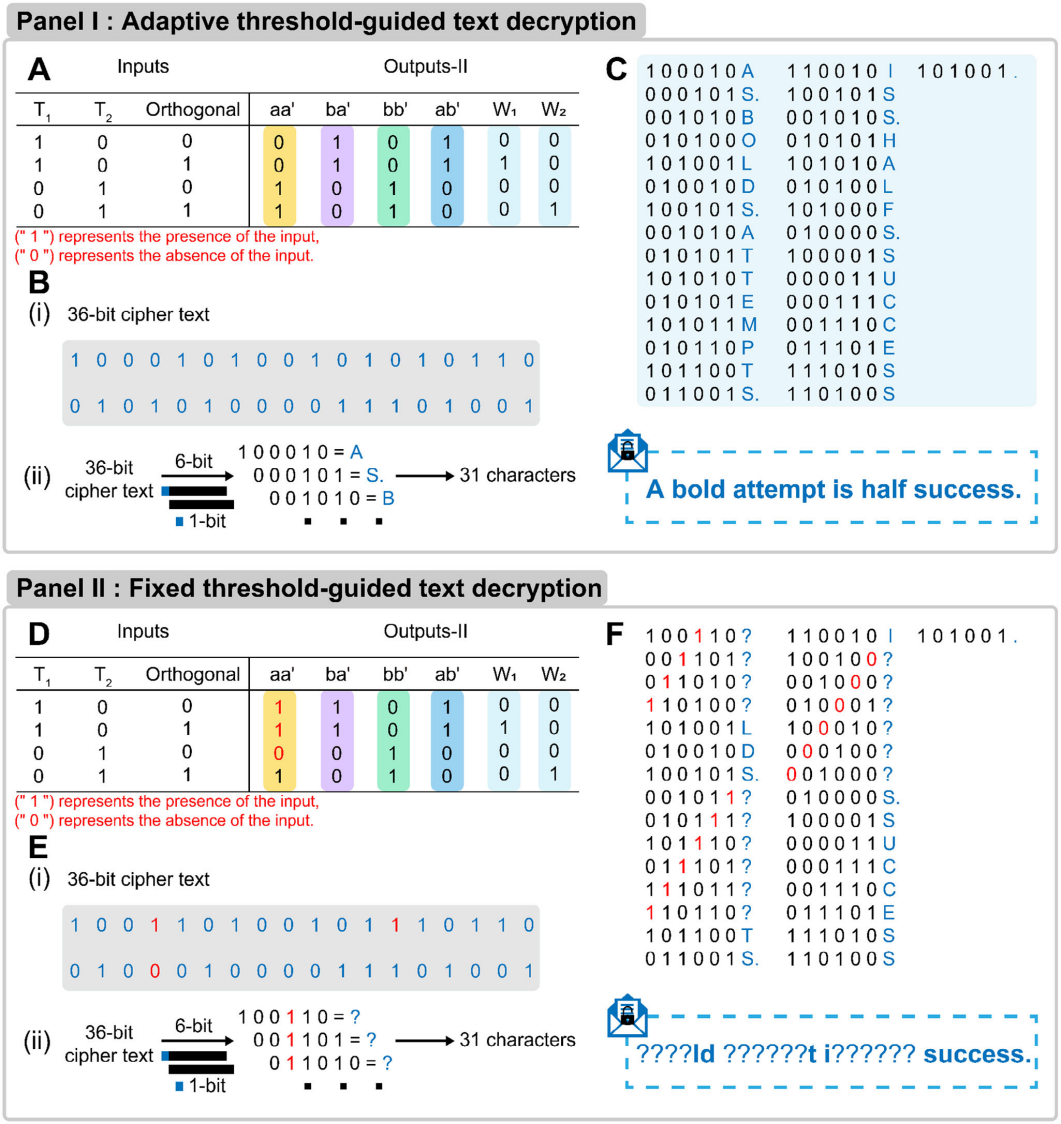
The CDN controlled over an orthogonal DNA molecular amplifier for information decryption. Panel I: adaptive threshold‐guided text decryption. A) The binary string of the inputs and outputs‐II. The inputs are according to the presence (“1”) or the absence (“0”) of the CDN and the orthogonal DNA molecular amplifiers. The outputs‐II relies on the readout patterns of the four constituents and W_1_, W_2_. The distributed data originated from Figures 2B,C and 4C,D. B) (i) A 36‐bit cipher text from the combination of inputs and outputs‐II shown in (A). (ii) The schematic description of the transposition ciphers protocol that rearranges the positions of the characters in the plaintext to produce the ciphertext. C) A 31‐letter cipher book and the corresponding plain text. “S.” represents space. Panel II: fixed threshold‐guided text decryption. D) The inputs and outputs‐II binary digits by substituting the aa' constituent in the CDN system with an individual aa'. E) (i) A 36‐bit cipher text from the combination of inputs and outputs‐II shown in (A). (ii) The schematic description of the transposition ciphers protocol. F) The cipher text is decrypted to incorrect plain text.

Realizing the programmable regulation of molecular amplifier by CDN system, we use binary codes to communicate a sentence. As shown in **Figure** **5**, panel I A, the inputs of the decryption system are according to the presence (“1”) or the absence (“0”) of the T_1_, T_2_, and the orthogonal DNA molecular amplifiers. The corresponding outputs‐II rely on the readout patterns of the four constituents (aa', ba', bb', and ab') and W_1_, W_2_ from the data originated from Figures 2B,C and 4C,D, respectively. The combination of inputs and outputs‐II provide a 36‐bit binary string, termed as cipher text (listed in Figure 5, panel I B(i)) by sequentially listing the binary digits. The decryption of the message based on the 36‐bit cipher text is ruled by a transposition cipher protocol that each 6‐bit represents for one letter and the following letter is represented by moving one bit afterwards, Figure 5, panel I B(ii). For example, “100 010” can be converted to the character “A”, and “000101” can be converted to the character “S.”. Based on that, a 31‐letter cipher text composed of 6‐bit digits is obtained and the corresponding plain text is used to transfer message of “A BOLD ATTEMPT IS HALF SUCCESS.”, as shown in Figure 5, panel I C. To demonstrate the high precision and accuracy of the proposed adaptive method, we substituted the aa' constituent in the CDN system with an individual aa', as illustrated in Figure 5, panel II. The experimental data derived from aa' (shown in Figure S15, Supporting Information) resulted in mis‐binary translation, as depicted in Figure 5, panel II F, where the misleading digits are highlighted in red. In order to further expand the scalability of the transmitted message, we have redefined the binary translation encoded by hierarchically classifying the threshold values that generates a 192‐bit ciphertext on the basis of a transposition cipher protocol, thereby expanding the capacity of decrypted text, the related discussion and detailed data is shown in Figures S28 and S29 (Supporting Information).

### 2.3 Constitutional Dynamic Network‐Guided Cascaded Molecular Amplifier for Message Decryption

**Figure 6:**
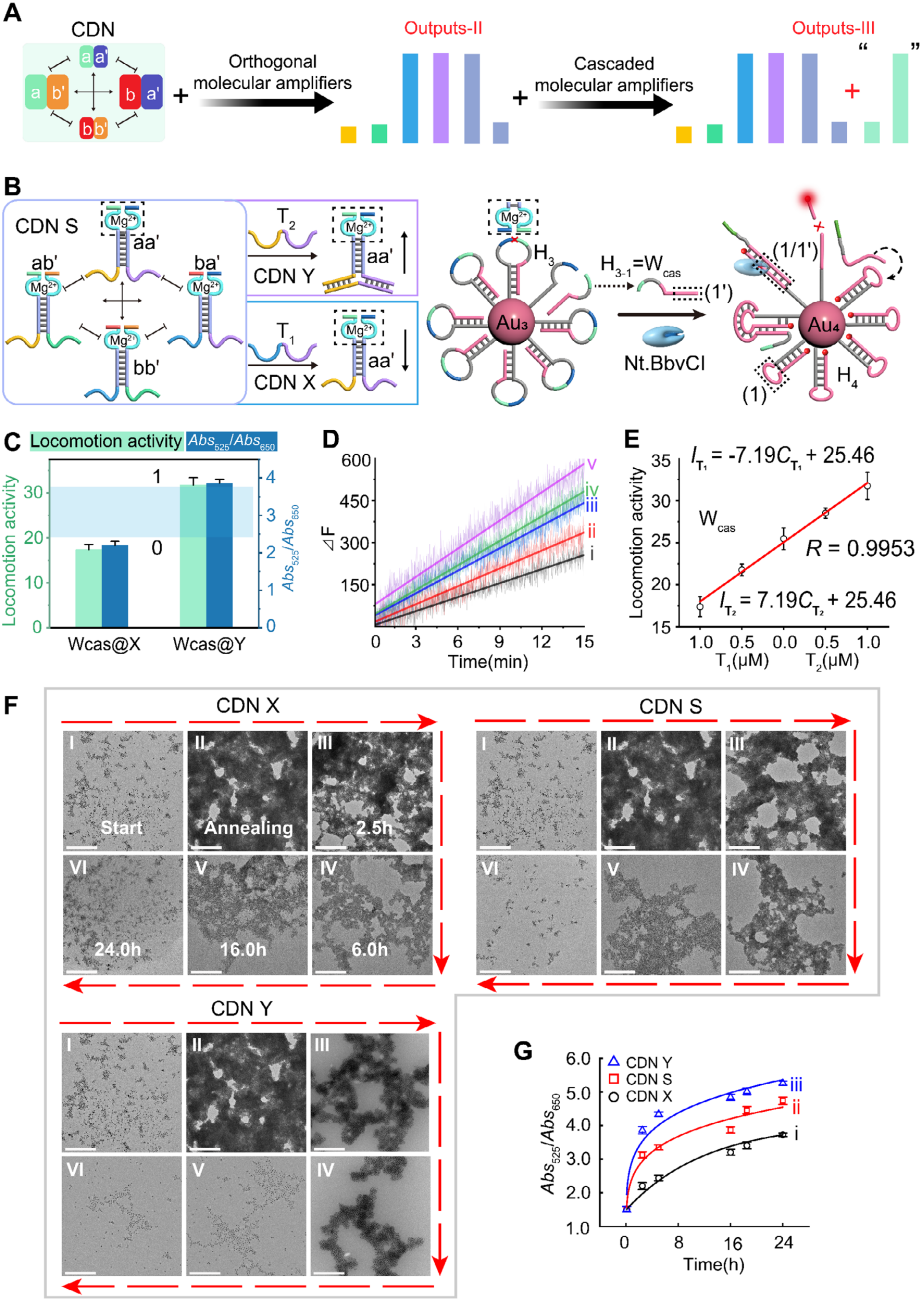
A) Schematic representation of the adaptive patterns (outputs‐III) expanded by utilizing the CDN system to control over the cascaded DNA molecular amplifiers. B) The schematic description of the CDN‐guided locomotion of the Au NP‐based cascade molecular amplifier. C) The distributed patterns and the systematic threshold of the CDN‐guided cascade walking system from the coordinately expression of the time‐varying UV‐*vis* absorbance ratio of Abs_525_/ Abs_650_ (blue column) and the locomotion activity of cascaded DNA walker from the fluorescent characterization (green column). The light blue area is the adaptive threshold. D) Time‐dependent fluorescence changes generated upon the cleavage of the H_4_ by the endonuclease at variable concentrations of triggers: (i) T_1_ = 1 µM, (ii) T_1_ = 0.5 µM, (iii) no T_1_ and T_2,_ (iv) T_2_ = 0.5 µM, and (v) T_2_ = 1 µM. E) The sensing curve of the locomotion activity of the cascaded DNA walker, W_cas_, as a function of trigger concentrations, derived from the data shown in (D). Error bars represent mean ± SD, *n* = 3. F) Time‐dependent TEM images corresponding to the dissociation of the bridged Au NPs generated by the equilibrated CDN “X”, CDN “S”, and CDN “Y”. Scale bar: 500 nm. G) Time‐dependent absorbance ratio changes of 525 nm and 625 nm upon: (i) The dissociation of the Au NPs subjected to CDN “X”. (ii) The dissociation of the Au NPs subjected to the equilibrated CDN “S”. (iii) The dissociation of the Au NPs subjected to the equilibrated CDN “Y”. Error bars represent mean ± SD, *n* = 3.

To explore the high volume of the transmitting message, a CDN regulated‐cascaded Au NPs‐based molecular amplifiers that is able to output two more patterns (outputs‐III) is set up, **Figure** **6**A. It is based on two nucleic acids‐functionalized Au NPs, Au_3_ and Au_4_. The DNA hairpin motif, H_3_, is immobilized on Au_3_, which contains a ribonucleobase‐site, rA, and could be recognized by the DNAzyme domain associated with aa'. (The quantification of H_3_ onto the Au NPs is depicted in Figure S30, Supporting Information). The cleaved fragments of H_3_, H_3‐1_ (the walker associated with cascaded system, termed as W_cas_) by the recognition of the aa' constituents, generates a toehold, (1′), that is captured by the free tether from H_4_ constructed on Au_4_. The H_4_ is modified with a fluorophore, ROX, that is quenched by Au_4_. (The quenching principle and the effect are discussed in Figure S31 (Supporting Information). The quantification of H_4_ onto the Au NPs is depicted in Figure S30, Supporting Information). The domain (1′) on H_3_ is engineered to hybridize to the loop domain (1) by generating an specific sequence of Nt.BbvCI, (1/1′). While in the absence of the aa', the two locked hairpins, H_3_ and H_4_ are unable to hybridize. When Nt.BbvCI is subjected to release the W_cas_ the less‐stable W_cas_ is able to hybridize to the loop domain in H_4_ by generating the nicking site of Nt.BbvCI, a duplex (1/1′). The domain (1/1′) is digested by Nt.BbvCI releasing the ROX‐modified tether on H_4_, which is accompanied by the fluorescence recovery of ROX. The W_cas_ is released from the Au NPs surface and hybridized to a neighboring H_4_, acting as a “walker” to move along the hairpin track on Au_4_, see Figure 6B. The characterization of the aa“‐constituents controlled over the cascaded DNA molecular amplifiers by polyacrylamide gel electrophoresis (PAGE) using DNA ladder as the reference and the preliminary fluorescence verification for the recognition domain in (1/1′) by the endonuclease are shown in Figure S32 (Supporting Information) and the accompanying discussion. By regulating the expression of aa” constituents via external trigger stimuli, the cleavage rates of the cascaded molecular amplifier can be controlled accordingly. (The locomotion of the cascaded molecular amplifier is preliminarily verified in a homogeneous phase as shown in Figure S33, Supporting Information). As can be observed from Figure 6D,E, the speed of the walker, W_cas_, increases under the increasing concentration of T_2_ and the decreasing subjection of T_1_. Since the H_3_ and H_4_ would be hybridizing and latterly be digested by aa' constituents, the CDN‐regulated the dissociation of the cascaded Au NPs is visibly characterized by TEM. A schematic representation of the cross‐talk between Au_3_ and Au_4_ is provided in supporting information, Figure S34 (Supporting Information). The TEM images of the cascaded, bridged Au NPs system controlled by different states of CDN are shown in Figure 6F. The two independent DNA‐functionalized Au NPs, Au_3_ and Au_4_ show negligible cross‐talk when simply mixing Au_3_ and Au_4_, as shown in Figure 6F(I). After annealing the mixture of Au_3_ and Au_4_ under the same concentrations, H_4_ is supposed to be opened by H_3_ through the recognition of the tether (1′) on H_3_ and loop domain (1) on H_4_. It can be observed that the degree of the aggregation of AuNPs is significantly enhanced due to the (1)/(1′) duplex‐bridged AuNPs, Figure 6F(II). After the subjection of different states of CDN into the bridged AuNPs, we can observe the dissociation of the Au NPs as a function of time, Figure 6F(III)–(VI). We can observe that the degree of Au NPs dissociation induced by CDN“X” is lower than that induced by CDN “S”, while the degree of Au NPs dissociation induced by CDN “Y” is higher than that induced by CDN “S”. The UV‐*vis* absorbance of the cascaded Au NPs system controlled by different states of CDN is shown in Figure 6G. The absorbance ratio of Abs_525_/Abs_650_ associated to cascaded Au‐NP system controlled by CDN “X” under a time‐interval of 40 h is shown in Figure S35 (Supporting Information). The associated absorbance spectra is shown in Figure S36 (Supporting Information). Based on data from Figure 6E,G, the cascaded locomotion activity and time‐varying absorbance ratio of Abs_525_/Abs_650_ in a bar presentation is shown in Figure 6C. The differential between different state of CDN‐guided cascaded molecular amplifier (Wcas@X and Wcas@Y) cooperatively generates an adaptive threshold to define the binary digital translation.

**Figure 7:**
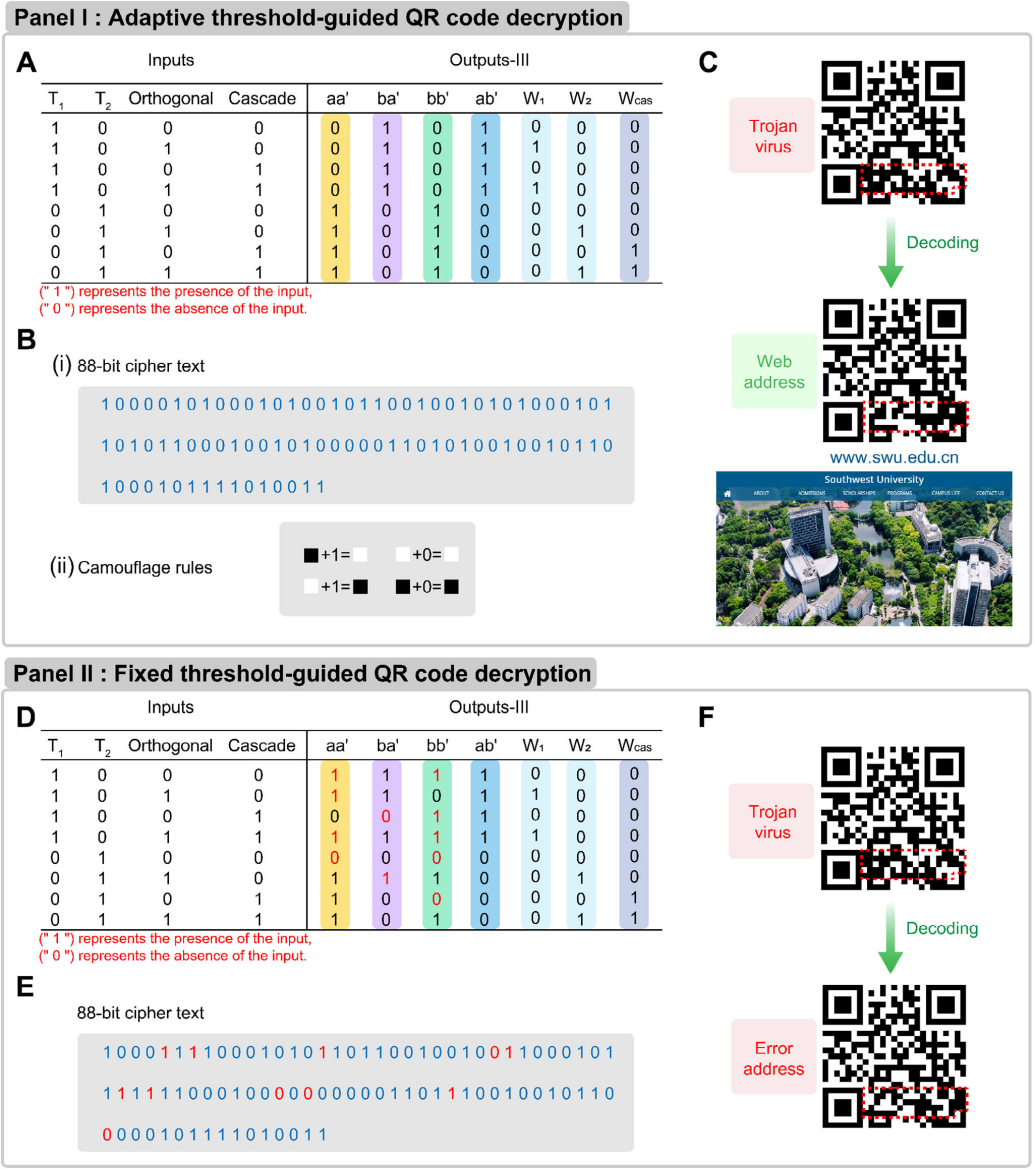
Panel I: adaptive threshold‐guided QR code decryption. A) Binary list of the CDN‐guided walking system. Inputs include the presence (“1”) or the absence (“0”) of the T_1_, T_2_, orthogonal, and cascaded molecular amplifiers. The outputs‐III includes the binary translation of four constituents of the CDN system, the W_1_, W_2_, and W_cas_ from the orthogonal molecular amplifier and the cascaded molecular amplifier. B) (i) An 88‐bit cipher text originated from (A). (ii) The camouflaging rules for decoding a QR code. “0” represents the true color of the domain and “1” represents the opposite color. C) The deciphering of a protected QR code by decoding the “red” region. Panel II: fixed threshold‐guided QR code decryption. D) The inputs and outputs‐III digital list by subsituting the aa', ba', and bb' constituent in the CDN system with an individual aa', ba', and bb'. E) An 88‐bit ciphertext generated from the procedure outlined in Figure 7D. F) The incorrect ciphertext fails to decode the “red” region in QR code. This figure highlights the impact of fixed constituent conversion on decryption accuracy and the inability to decode specific regions of the QR code under such constraints.

To further extend the expression volume of the cipher text and expand the diversity of the message decryption, a camouflaged decryption model for QR code is constructed based on the CDN‐guided cascaded molecular amplifiers. As shown in **Figure** **7**, panel I A, the inputs of the decryption system are based on the presence (“1”) or the absence (“0”) of the T_1_, T_2_, orthogonal DNA molecular amplifiers including Au_1_ and Au_2_, and the cascaded system including Au_3_ and Au_4_. The binary translated outputs‐III originates from the readout patterns of the four constituents (aa', ba', bb', and ab“), W_1_, W_2_, and W_cas_ from data in Figures 2, 4, and 6C, respectively. It should be noted that the column of “cascade” in inputs list and the W_cas_ in outputs‐III list are the two newly added columns due to the introduction of the cascaded walking system. Based on the four columns of inputs and seven columns of outputs‐III, an 88‐bit cipher text is obtained by sequentially listing the binary digits of the inputs/outputs‐III, Figure 7, panel I B(i). The “Receiver” would decrypt the camouflaged QR code (the region marks in “red”, Figure 7C) according to the 88‐bit cipher text based on the camouflaging rules shown in Figure 7, panel I B(ii). The red domain of the QR code is divided into 88 domains that are determined as black or white. Each camouflaging domain is unveiled by the corresponding binary digits that “0” represents the true color of the domain and “1” represents the opposite color (black to white, or white to black). The “Receiver” gets a true QR message by translating each domain of the red region from the QR code, or they get the trojan virus as an attack. The website of Southwest University is taken as an example in this study (Figure 7, panel I C). As a comparison, we substituted the aa”, ba', and bb' constituent in the CDN system with an individual aa', ba', and bb', as illustrated in Figure 7, panel II. The experimental data derived from aa', ba', and bb' (shown in Figures S15,S37, and S38, Supporting Information) resulted to mis‐binary translation, as depicted in Figure 7, panel II E, which leads to the misleading digits (highlighted in red) and incorrected decoding of the QR code, Figure 7E. According to the series of translation decryption from the individual constituent (aa' or aa', ba', and bb') and the CDN‐engineered system, including location translation (Figure 3), text translation (Figure 5) and the QR code translation (Figure 7), it can be concluded that the adaptive threshold derive from CDN shows high accuracy and precision than that of the artificial model.

## 3 Conclusion

In summary, we have outlined an effective and reliable strategy to generate an adaptive threshold by programmed DNA dynamic network, which enables highly authentic information decryption. The CDN system is further introduced as functional units that control and dictate the guided locomotion of gold nanoparticles‐based molecular amplifiers, which greatly improve the volume of the message transmission. This work not only enriches the application of DNA‐based system chemistry with manipulation of the molecular amplifier but also provides a highly authentic strategy for information decryption based on DNA nanoassemblies. Nevertheless, the proposed adaptive method still has some limitations. Due to the antagonistic interactions among the constituents, the difference value derived from the four outputs, termed as threshold value, is constrained. For instance, when aa' and bb' are minimized, the maximum values of ab' and ba' are determined, which restricts the volume of text that can be translated using this approach. Despite this, we have redefined the binary translation encoding by adjusting the threshold values, thereby expanding the capacity of decrypted text, as shown in Figures S28 and S29 (Supporting Information). By rational subjection of an anti‐trigger nucleic acid that could hybridize to the DNA stimuli toward the CDN system, a rewritable information processor is expected to be developed. By designing a transiently assembled DNA decryption method, it is possible to achieve automatic recovery of information after instantaneous decryption, thereby preventing subsequent information leakage.

## Conflict of Interest

The authors declare no conflict of interest.

## Supporting information



Supporting Information

## Data Availability

The data that support the findings of this study are available from the corresponding author upon reasonable request.
