# Supplementary material for: A Programmed DNA Dynamic Assembly‐Guided Molecular Amplifier for Authentic Information Decryption
Source: Adv Sci (Weinh). 2025 May 19;12(22):2409586. doi: 10.1002/advs.202409586 (PMC12165106; doi:10.1002/advs.202409586)
Supplement: Supplementary file 1 — Supporting Information [file ADVS-12-2409586-s001.docx]

**Supporting Information**

**A Programmed DNA Dynamic Assembly-Guided Molecular Amplifier for Authentic Information Decryption**

Ning Yang, Jie Zhou, Fengying Yuan, Yajuan Liu, Jia Zhang, Ying Zhuo, Yu Ouyang, Yaqin Chai, Pu Zhang, * Ruo Yuan *

*Key Laboratory of Luminescence Analysis and Molecular Sensing (Southwest University), Ministry of Education, Chongqing Engineering Laboratory of Nanomaterials & Sensor Technologies, College of Chemistry and Chemical Engineering, Southwest University, Chongqing 400715, P.R. China.*

*Tel: +86-023-68252277*

*E-mail address: puzhang@swu.edu.cn (Pu Zhang); yuanruo@swu.edu.cn (Ruo Yuan)*

**Table of Contents**

**Experimental Section S4**

Materials S4

Instruments S4

Measurements S5

Methods S5

**Results and Discussion S7**

Figure S1. Simulated design of CDN reaction system in different states using NUPACK S7

Figure S2. Polyacrylamide gel electrophoresis (PAGE) characterization of the formation of CDN system and the conversion of CDN “S” to “X” or “Y” S9

Figure S3. Calibration curves of CDN constituents (HITACHI F7100 Fluorometer) S10

Figure S4. Preliminary experiments: Time-dependent fluorescence verification of CDN conversions S11

Figure S5. Calibration curves of CDN constituents (Agilent Cary Eclipse Fluorometer) S12

Figure S6-S7. Photo-triggered conversion of equilibrated CDN “S” to CDN “Y” S13

Figure S8. Time-dependent fluorescence of adaptive defined-threshold associated with CDN system S14

Figure S9-S14. The kinetics simulation of CDN “S” converted to CDN “X” or CDN “Y” S15

Figure S15-S16. Binary translation based on individual aa'-based constituent S20

Figure S17. Selectivity of the CDN system towards the external stimuli DNA S21

Figure S18-S19. The combined subjection of T_1_ and T_2_ to CDN system S22

Figure S20-S21. Validation of the gold nanoparticles (Au NPs) and the DNA-functionalized Au NPs S24

Figure S22. Quantification of DNA (S_5_ and S_6_) assembled on Au NPs S26

Figure S23. PAGE characterization of the CDN system controlled over the orthogonal DNA molecular amplifiers S27

Figure S24. UV-*vis* absorbance and fluorescence verification of the quenching effect of S_5_ (TAMRA) and S_6_ (FAM) by Au NPs S28

Figure S25. Time-dependent fluorescence verification of the formation of DNA walkers (W_1_ and W_2_) S29

Figure S26. Preliminary experiments: Time-dependent fluorescence verification of the CDN system controlled over the orthogonal DNA molecular amplifiers in a homogeneous phase S30

Figure S27. Time-dependent fluorescence characterization of the CDN system controlled over the orthogonal Au NPs-based DNA molecular amplifiers S30

Figure S28-S29. A complicated text decrypted by hierarchically classifying the adaptive thresholds S31

Figure S30. Quantification of DNA (H_3_ and H_4_) assembled on Au NPs S33

Figure S31. UV-*vis* absorbance and fluorescence verification of the quenching effect of H_4_ (ROX) by Au NPs S34

Figure S32. Preliminary experiments: Polyacrylamide gel electrophoresis (PAGE) and time-dependent fluorescence characterization of the aa'-regulated cascaded DNA molecular amplifiers S35

Figure S33. Preliminary experiments: Time-dependent fluorescence verification of the CDN-controlled over the cascaded DNA molecular amplifiers in a homogeneous phase S36

Figure S34-S36. Control over the dissociation of aggregated Au NPs by CDNs S37

Figure S37. Binary translation based on individual ba'-based constituent S39

Figure S38. Binary translation based on individual bb'-based constituent S40

Table S1. Computational simulation rate constants of the transitions of CDN “S” to CDN “X” S15

Table S2. Computational simulation rate constants of the transitions of CDN “S” to CDN “Y” S15

Table S3. The comparison between the proposed adaptive method and other DNA-based encryption methods S23

**References S41**

**Experimental Section**

**Materials**

The materials used in this study are shown as follows. All DNA and RNA oligonucleotides are synthesized by Sangon Biotech Co., Ltd. Hydrochloric acid (HCl), trisodium citrate dihydrate (C_6_H_5_Na_3_O_7_$\text{∙}$2H_2_O), methanol (CH_3_OH) and nitric acid (HNO_3_) are purchased from Chongqing Chuandong Chemical Industry Company. Hydrogen tetrachloroaurate (III) trihydrate (HAuCl_4_$\text{∙}$3H_2_O, 99.99%) are purchased from Shanghai Adamas-beta Company. NaH_2_PO_4_, Na_2_HPO_4_, dithiothreitol (DTT) and tris (2-carboxyethyl) phosphine hydrochloride (TCEP) are provided by Shanghai Macklin Co., Ltd. NaCl, MgCl_2_$\text{∙}$6H_2_O and 2-[4-(2-Hydroxyethyl) piperazin-1-yl] ethane sulfonic acid sodium salt (HEPES) are purchased from MoLai chemistry Co., Ltd. (Hebei, China). Sodium dodecyl sulfate (SDS) is purchased from Tianjin heowns Biochemical Technology Co., Ltd. Tween-20 is obtained from Sunshine Biotechnology Co., Ltd. (Nanjing China). Agarose, hydrogen peroxide (H_2_O_2_), acryl/bis 40% solution (29:1) and N, N, N', N'-tetramethyl ethylenediamine (TEMED) are purchased from Sangon Biotech Co., Ltd. (Shanghai, China). Sucrose, phosphotungstic acid 44-hydrate and bis (p-sulfonatophenyl) phenylphosphine dihydrate dipotassium salt (BSPP) are provided by Aladdin Co., Ltd. (Shanghai, China). Nicking enzyme, Nt.BbvCI, (10,000 units·mL^−1^, 2.3 μM), CutSmart® Buffer are purchased from New England Biolabs Inc.. The chemical reagents are all of analytical grade and used with no further purification. The ultrapure water of 18.2 MΩ$\text{∙}$cm is used throughout the experiments.

The sequences of nucleic acids used in the study are listed as follows:
a: 5'-GATATCAGCGATCAAAATACTTACAGACACAACAA-3'

a': 5'-ACAGAAGAACCGTAAGTATTTTGCACCCATGTTACTCT-3'

b: 5'-CTGCTCAGCGATCAAAATACTTACCCCATCACAAAAATTT-3'

b': 5'-AAGTAAGTATTTTGCACCCATGTTCGTCA-3'

a_mod_:5'-GATATCAGCGATCAAAATACTTACAGACACAACAATCTCTCTCTCTCTCTCTCTCTCTCTCTCTCTCTCTCTCTCTCTCTCTCTCTCTCTCTCTC-3'

$\text{b'}_{\text{mod}}$: 5'-TCTCTCTCTCTCTCTCTCTCTCTCTCTCTCTCTCTCAAGTAAGTATTTTGCACCCATGTTCGTCA-3'

$\text{T}_{\text{1}}$: 5'-AAAGGTTTGTGATGGACGTTCTTCTGTC-3'

$\text{T}_{\text{2}}$: 5'- CACAGAAGAACGTGACACAACAACG-3'

T_2_': 5'- CGTTGT/iSpPC/TGTGTC/iSpPC/ACGTTC/iSpPC/TTCTGTG-3'

Sub1 (aa'): 5'-FAM-AGAGTAT**rA**GGATATC-BHQ1-3'

Sub2 (bb'): 5'-ROX-TGACGAT**rA**GGAGCAG-BHQ2-3'

Sub3 (ba'): 5'-CY5-AGAGTAT**rA**GGAGCAG-BHQ2-3'

Sub4 (ab'): 5'-ROX-TGACGAT**rA**GGATATC-BHQ2-3'

Sub5 (W_1_): 5'- BHQ2-ACTGAAT**rA**GGAGGAC- TAMRA -3'

Sub6 (W_2_): 5'- BHQ1-ACTGAAT**rA**GGAACAG-FAM-3'

Sub1-noFQ (aa'): 5'-AGAGTAT**rA**GGATATC-3'

Sub2-noFQ (bb'): 5'- TGACGAT**rA**GGAGCAG-3'

Sub3-noFQ (ba'): 5'- AGAGTAT**rA**GGAGCAG-3'

Sub4-noFQ (ab'): 5'- TGACGAT**rA**GGATATC-3'

miRNA-21: 5'- UAGCUUAUCAGACUGAUGUUGA-3'

miRNA-221: 5'- AGCUACAUUGUCUGCUGGGUUUC-3'

S_1_: 5'- CTTGACCACTTGCCGTAGCACCCATGTTTCAGT-3'

S_2_: 5'- GTCCTCAGCGATCTACGGGTCACTGTCTAC-3'

S_3_: 5'- ACACAGAAGATCCTAGAGCACCCATGTTTCAGT-3'

S_4_: 5'- CTGTTCAGCGATCTCTAGCCATCACAAAGC-3'

H_1_: 5'- ***CCACTTGGTCACTG***ATTAGAGTAT**rA**GGAGCAGGTAGA***CAGTGACCAAGTGG***TCAAG-3'

H_2_: 5'- ***GAAGATCCCATCAC***TTTAGAGTAT**rA**GGATATCGCTTT***GTGATGGGATCTTC***TGTGT-3'

H_1-1_: 5'- GGAGCAGGTAGACAGTGACCAAGTGGTCAAG-3'

H_2-1_: 5'- GGATATCGCTTTGTGATGGGATCTTCTGTGT-3'

S_5_: 5'-HS-SH-TTTTACTGAAT**rA**GGAGGAC- TAMRA -3'

S_6_: 5'-HS-SH-TTTTACTGAAT**rA**GGAACAG-FAM-3'

H_3_:5'-HS-SH-TTTTTTTTTTTTTTT***AATCACC***ATTTTTAAAGAGTAT**rA**GGATATCGAAGCTGA***GGTGATT***-3'

H_3-1_: 5'-GGATATCGAAGCTGAGGTGATT-3'

H_4_ (FQ): 5'-***GAAGCTGAG***AA/dTROX/CAC**^∇^*CTCAGCTTC***/dTBHQ2/TTTTTTTTT-3'

H_4_: 5'- HS-SH-TTTTTTTTTT***GAAGCTGAG***AATCAC**^∇^*CTCAGCTTC***-ROX-3'

The ribonucleobase cleavage site, **rA**, in different substrates associated with Mg^2+^-ion-dependent DNAzymes are indicated in bold, the corresponding Mg^2+^-ion-dependent DNAzymes sequence are underlined. The specific sequences of complementary base pairing at the stem of the hairpins are indicated in italics and bold. “**^∇^**” indicates the cleaving site of Nicking enzyme, Nt.BbvCI. The iSpPC modified in T_2_' refers to the photo-sensitive o-nitrobenzyl phosphate photoresponsive moieties.

**Instruments**

All the concentration of nucleic acid is determined from their absorbance at 260 nm using Nanodrop (KAIAO K5600C, China). Fluorescence spectra are recorded with Fluorometer (Hitachi F7100, Japan and Agilent Cary Eclipse, USA). Oil bath magnetic stirring heater (Yuhua ZNCL-G130*70, China) is employed for synthesizing the gold nanoparticles (Au NPs). Transmission electron microscopy (Talos F200X, Czech Republic) is used to characterize the morphology and the aggregation extent of the Au NPs. UV-*vis* absorbance spectra are recorded by a spectrophotometer (Shimadzu UV-2450, Japan). Polymerase chain reaction amplifier (Eastwin ETC811, China) is employed to anneal the CDN systems and related nucleic acid-based reaction samples. The Zeta potentials are determined by Zetasizer Nano particle analyzer series (Malvern ZEV3600, England). Agarose gel electrophoresis apparatus used horizontal gel electrophoresis system (Bolant BLT-31DS, China). Polyacrylamide gel electrophoresis by a vertical gel electrophoresis system (Bio-Rad Mini-PROTEAN®, USA). The PAGE gel is visualized *via* the gel imaging system (Bio-Rad ChemiDoc XRS+, USA). UV irradiation was conducted by an UV Transilluminator device (Clinx Science Instruments CUV 10, China) under 1.59 mW/cm^2^. The optical power meter (Perfectlight Technology PL-MW2000, China) is used to measure the optical power.

**Measurements**

The excitation of FAM, ROX, Cy5 and TAMRA are performed at 497, 583, 633 and 546 nm, respectively. The time-dependent emission of FAM, ROX, Cy5 and TAMRA are recorded at 520, 606, 666 and 579 nm, respectively.

It should be noted that the fluorescent measurements throughout the study are conducted by two fluorometers, HITACHI F7100 Fluorometer and Agilent Cary Eclipse Fluorometer. Figure S3, S4, S13A, S14A, S17, S28, S29, S33 are recorded by HITACHI F7100 Fluorometer, Figure S5, S7, S8, S11, S13B, S14B, S15, S16, S20, S26, S27, S31 are recorded by Agilent Cary Eclipse Fluorometer.

**Methods**

**Preparation of CDNs.**

The CDN “S” consists of the constituents aa', ab', ba' and bb' are prepared as follows. The mixture of a, a', b, and b' (1 μM each) in HEPES buffer (10 mM, pH = 7.2, containing 20 mM MgCl_2_) is annealed at 65°C for 15 min, then cooled to 25°C at a rate of 1°C every 3 min, and finally equilibrated at 25°C for 2 h, yielding a mixture of aa', ab', ba' and bb', CDN “S”. For the conversion of CDN “S” to CDN “X” or CDN “Y”, mixtures of aa', ab', ba' and bb' (CDN “S”, 0.5 μM) are triggered accordingly by $\text{T}_{\text{1}}$ or $\text{T}_{\text{2}}$ (1 μM) and equilibrated at a time-interval of 2 hours at 37°C to produce CDN “X” or CDN “Y”, respectively.

**Probing the constituents in the CDNs.**

An equilibrium mixture of 50 μL of aa', ab', ba', and bb' (CDNs) is treated with probing constituent-related fluorophore/quencher-modified substrate, and non-modified substrates associated with the non-probing constituents. For example, to probe the content of constituent aa', 50 μL of CDNs (0.5 μM) equilibrium mixture is treated with the following substrates: sub1, sub2-noFQ, sub3-noFQ, and sub4-noFQ, 2.5 μL of 100 μM each. Subsequently, time-dependent fluorescence changes generated by aa'-associated Mg^2+^-ion-dependent DNAzyme that cleaves sub1 are tracked. the contents of constituents in different CDNs are based on the calibration curves corresponding to the cleavage rates of different substrates digested by intact constituents at different concentrations.

**Kinetically probing the CDNs conversions, CDN “S” to CDN “X” or CDN “Y”.**

The preparation of CDN “S” is followed as mentioned above. The fluorescent tracking of conversion of CDN “S” to CDN “X” is taken as an example. Mixtures of aa', ab', ba' and bb' (CDN “S”, 0.5 μM) are triggered accordingly by $\text{T}_{\text{1}}$ (1 μM, 0.75 μM or 0.5 μM) and equilibrated at a time-interval of 0 min / 5 min / 10 min / 15 min / 30 min / 60 min at 37°C, respectively. The test method is consistent with that in “Probing the constituents in the CDNs” and maintained at 25 °C.

**Preparation of gold nanoparticles.**

The gold nanoparticles (Au NPs) are synthesized according to the sodium citrate-based reduction method. ^[1]^ All glassware and magnetic stirrer rotor are pretreated with aqua regia (HCl/$\text{HNO}_{\text{3}}$, 3:1) for 24 h. After soaking in aqua regia, the round bottom flask and magnetic stirrer rotor are washed with distilled water and sonicated by sonicator for 15 min. At first, 50 mL of $\text{HAuCl}_{\text{4}}$ (1.0 mM) is heated to boiling under vigorous stirring, then 5 mL of trisodium citrate (38.8 mM) is rapidly injected. After that, the color of the solution changed from light yellow to black within 20 seconds and finally turned burgundy after 70 seconds. The burgundy solution is kept boiling for another 20 min. After stopping heating, the colloid is stirred until the prepared solution reached to room temperature. The prepared Au NPs are stored at 4 °C for further using.

**Preparation of DNA (S_5_ and S_6_)-functionalized Au NPs.**

DNA-functionalized Au NPs are synthesized according to previous reports with minor modifications. ^[2]^ To activate the thiol-DNA (S_5_ and S_6_), 100 μL of 100 μM S_5_ or S_6_ is added to 40 μL of 20 mM Tris buffer (pH 7.0) containing 10 mM TCEP. The resultant solution is incubated for 8 h at room temperature. After incubation, the activated oligonucleotides are added to 3 mL of gold colloidal solution to functionalize the Au NPs through Au-S bond. The mixed solution is sonicated for 10 s, and then incubated for 25 min under continuous shaking at room temperature. After that, the resultant solution is mixed with 0.1 M phosphate buffer (pH 7.2) containing 0.1% SDS and the final concentrations of phosphate and SDS are brought to 0.01 M and 0.01%, respectively. The solution is sonicated for 10 s, and incubated for 25 min. In the subsequent salt aging process, the concentration of NaCl is first increased to 0.05 M by using 2 M NaCl, and then it gradually increased by 0.1 M each time until the final concentration of NaCl is 0.65 M. After each addition of NaCl, the DNA-Au NPs are vortexed, and sonicated for 10 s, and then incubated for 25 min. After the salt aging treatment, the mixture is shaken at room temperature overnight. To remove the excess DNA, the solution is centrifuged at 13,000 rpm for 30 min, and then redispersed in reaction buffer (pH 8.0) containing 20 mM Tris, 200 mM NaCl, 5 mM MgCl_2_, and 0.05% Tween-20. Tween-20 is used to reduce the sticking of Au NPs inside the centrifuge tubes. This step is repeated three times to sufficiently remove the excess DNA. The preparation of DNA-functionalized Au NPs are stored at 4 °C in dark for using.

**Preparation of phosphotungstic acid stained-Au NPs-DNA for TEM characterization.**

Phosphotungstic acid staining DNA functionalized-gold nanoparticles according to previous reports with minor modifications. ^[3]^ 10 μL of DNA functionalized-Au NPs (10 nM) sample is dropped on the copper grid for 10 min, then the copper grid is dried by filter paper, and 10 μL of phosphotungstic acid solution (3%, pH = 7.0) is dropped on the copper grid for 1 min to stain the nucleic acids, remove the excess dye solution, clean with ultrapure water three times, and dry completely at room temperature.

**Preparation of BSPP protected-Au NPs for the agarose gel analysis.**

The method for preparation of BSPP protected-Au NPs is adapted from previous studies. ^[4]^ In detail, 3 mg of bis (p-sulfonatophenyl) phenylphosphine dihydrate dipotassium salt (BSPP) is added to 10 mL Au NPs (10 nM) and the mixture is shaken overnight at room temperature. 58.4 mg NaCl (solid) is added slowly to this mixture while stirring until the colour changed from deep burgundy to light purple. The resulting mixture is centrifuged at 8,000 rpm for 5 min, and the supernatant is removed. The treated Au NPs are then resuspended in 0.3 mL BSPP solution (0.3 mg·mL^–1^), and mixed with 0.3 mL methanol. The mixture is again centrifuged, the supernatant is removed and the Au NPs are resuspended in 100 µL BSPP solution (0.3 mg·mL^–1^).

**Quantification of DNA (S_5_ and S_6_) assembled on Au NPs.**

To quantify the loading of S_5_ and S_6_, DTT (20 mM) is added into 100 μL Au NPs-DNA solution (5 nM), so that S_5_ and S_6_ are completely released from the gold nanoparticles. After overnight shaking at room temperature, the sample is centrifuged to separate the released DNA (S_5_ and S_6_)**_,_** and the fluorescence spectrum of the supernatant containing fluorophore-modified S_5_/S_6_ are measured.

**Preparation of the hairpins H_1_, H_2_.**

Taking H_1_ as an example, 10 µM of H_1_ in HEPES buffer (10 mM, pH = 7.2, containing 10 mM MgCl_2_) is annealed at 95 ^o^C @ 5 min, and cooled instantly to 25 ^o^C, and allowed to equilibrate for 2 hours at 25 ^o^C.

**Control over orthogonal DNA molecular amplifiers by CDNs.**

The CDN “S”, “X” and “Y” are obtained by following the above-mentioned procedure. Taking CDN “X” control over orthogonal DNA molecular amplifiers as an example, the equilibrated mixture of CDN “X” (25 μL, 1 μM) is subjected to the hairpins H_1_ (5 μL, 10 μM) and H_2_ (5 μL, 10 μM) and to the strands S_1_, S_2_, S_3_, and S_4_ (5 μL, 10 μM). The mixture is incubated at 25 ^o^C for 4.5 hours. Prior to testing, 10 μL of Au_1_-S_5_ and Au_2_-S_6_ are added (5 nM). The reaction sample is finally diluted in 50 µL HEPES buffer (10 mM, pH = 7.2, containing 10 mM MgCl_2_).

**Preparation of DNA (H_3_ and H_4_)-functionalized Au NPs.**

H_3_ and H_4_ are annealed at 95 °C for 5 min and then stabilized at 25 °C for 2 hours. 100 μL of 33 μM H_3_ or H_4_ is added to 40 μL of 20 mM Tris buffer (pH 7.0) containing 10 mM TCEP to activate the thiol-DNA (H_3_ and H_4_). The resultant solution is incubated for 8 h at room temperature. After incubation, the activated oligonucleotides are added to 0.5 mL of gold colloidal to functionalize the Au NPs. The other procedures of H_3_ and H_4_ functionalized on Au NPs are following the above-mentioned procedure of DNA (S_5_ and S_6_)-functionalized Au NPs.

**Quantification of DNA (H_3_ and H_4_) assembled on Au NPs.**

To quantify the loading of H_3_ and H_4_, DTT (20 mM) is added into 100 μL Au NPs-DNA solution (5 nM), so that H_3_ and H_4_ are completely released from the gold nanoparticles. After overnight shaking at room temperature, the sample is centrifuged to separate the released DNA (H_3_ and H_4_), and the UV-*vis* absorbance spectrum and fluorescence spectrum of DNA (H_3_ and H_4_) are measured.

**Formation of Au NPs aggregation by DNA (H_3_ and H_4_)-crosslink.**

Typically, thiol–DNA(H_3_)-modified Au NPs (10 nM) and DNA(H_4_)-modified Au NPs (10 nM) are mixed. The mixture is heated to 70 °C and incubated for 5 min. After that, the solution is cooled to room temperature, during which the color of the solution changed from burgundy to purple, and finally a large amount of aggregates precipitated at the bottom of the centrifuge tube. To ensure the homogeneity and reproducibility of Au NP aggregates, all solutions are vigorously agitated before use.

**Control over the dissociation of aggregated Au NPs by CDNs.**

The CDN “S”, “X” and “Y” are obtained by following the above-mentioned procedure. Taking CDN “S” control over the dissociation of aggregated Au NPs as an example, the equilibrated mixture of CDN “S” (10 μL, 0.415 μM) is added to a solution (50 μL) of the as-prepared DNA-crosslinked Au NPs aggregates to initiate the disassembly of Au NPs by the recognition of the rA domain engineered in H_3_. The mixed solution is incubated for 24 h at 25 °C, and the time-interval absorbance spectra are monitored using a UV-*vis* spectrometer. Transmission electron microscopy (TEM) is used to characterize the shape of mono-dispersed Au NPs and Au NPs aggregates before and after digestion by adding CDNs. The sample is prepared by dropping 5 μL of sample solution onto a carbon-coated copper grid and drying at room temperature.

**Control over cascaded DNA molecular amplifiers by CDNs.**

The CDN “S”, “X” and “Y” are obtained by following the above-mentioned procedure. Taking CDN “X” control over cascaded DNA molecular amplifiers as an example, the equilibrated mixture of CDN “X” (19 μL, 1 μM) is subjected to the Au_3_-H_3_ (6 μL, 10 nM), incubated at 25 ℃ for 4 h, then the Au_4_-H_4_ (5 μL, 10 nM) and 1.8 µL Nt.BbvCI with a concentration of 5555 units·mL^−1^ (≈1.2776 μM) and 5 µL 10 × CutSmart® Buffer are added, and incubated at 37 ℃ for 15 min before testing. The reaction sample is finally diluted in 50 µL HEPES buffer (10 mM, pH = 7.2, containing 10 mM MgCl_2_ and 1 × CutSmart® Buffer). Noting that the Nt.BbvCI (3 μL, 2.3 μM) are treated by H_2_O_2_ (2.4 μL, 50 mM) for 4 hours to reduce the effect of dithiothreitol in nicking enzyme storage solution.

**Results and Discussion**

**Simulated design of CDN reaction system in different states using NUPACK**

The engineering design of the CDN reacting system requires following procedures. Based on the constitutional dynamic exchanging properties, the engineering design of these sequences was carried out by NUPACK software tool, ensuring the stability, specificity and functionality. The simulated hybridizing scheme of each constituent presented in NUPACK is showed in Figure S1A. The rigid duplex stems (marked in blue) in each constituent are of equal length (12 bases) and have identical thermodynamic stability (the calculated thermodynamic free energy of the four constituents is basically the same), which enable the dynamic exchange varied in different constituents. It is worth noting that although the concentrations of the four constituents calculated by NUPACK are not equal, the concentrations of the constituents obtained by subsequent experiments are basically equal. NUPACK is usually capable of calculating thermodynamically feasible scenarios, whereas actual conditions may vary due to factors such as metal ions and the composition of the reaction buffer solution. The loop domain (marked in red) represents a typical E6 DNAzyme structure, enables the fluorescent signal output through cleavage of a fluorophore/quencher-labeled substrates. The tether domain (marked in green) is engineered to recognize the DNA stimuli (T_1_ or T_2_) for the reconfiguration of the dynamic system. The hybridized complex of trigger strand (T_1_ or T_2_) to tether strand is more stable and the free energy is lower (-18.07 kcal/mol → -42.80 kcal/mol), resulting in dynamic competition among the constituents. Such as subjection of T_1_ to CDN “S” stabilizes the constituent ba' by the dissociation of constituents, aa' and bb', thus increasing the content of ab' from the hybridization of the residual components a and b'. As a result, the CDN “S” transfers to CDN “X” by dynamically exchanging the components from the corresponding constituents. The structures of the constituents after subjection of a trigger and their populations also can be predicted in NUPACK software, see Figure S1B and S1C.

**
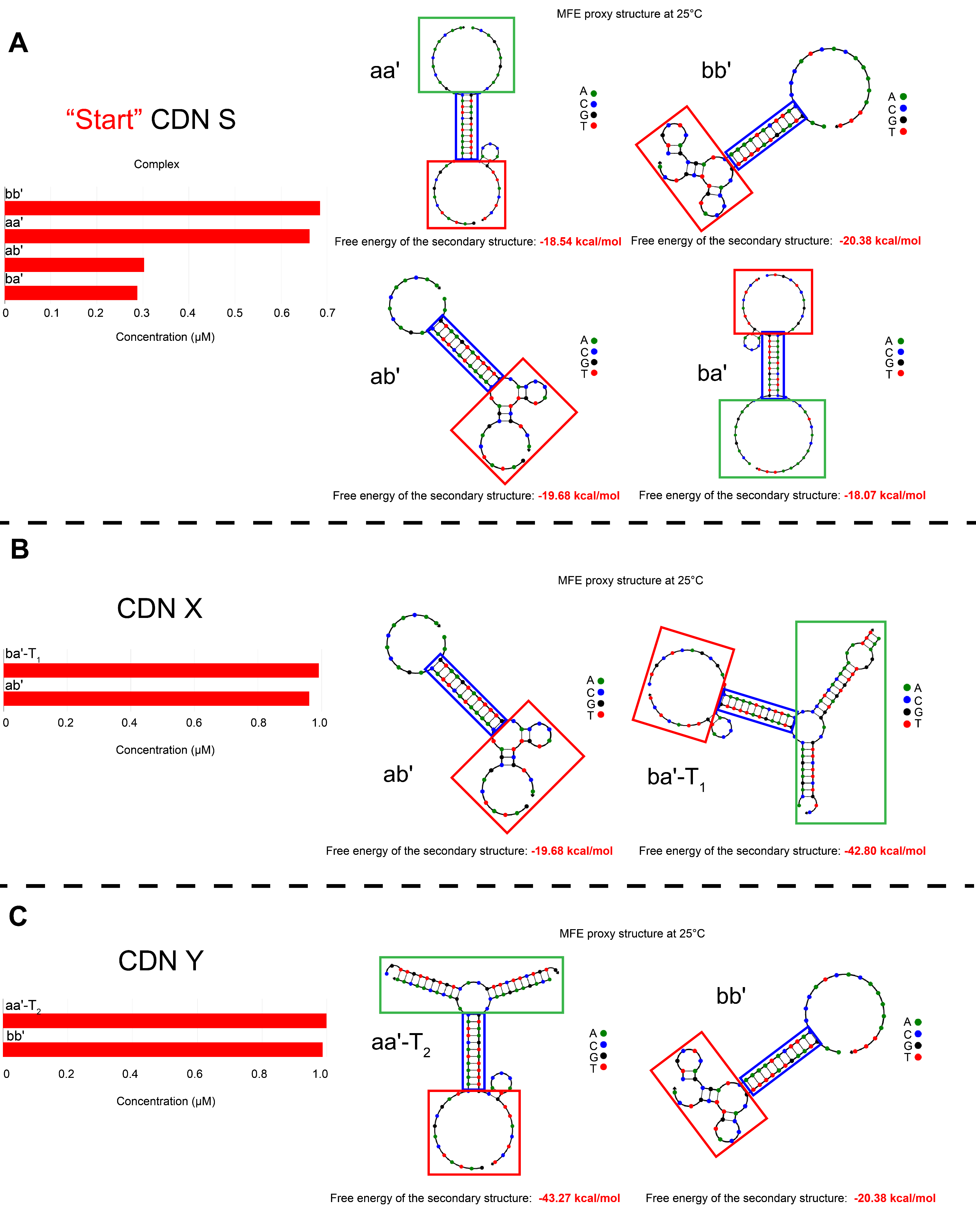
**

**Figure S1.** (A) The simulated hybridizing scheme of each constituent in CDN and the corresponding populations in CDN “S” presented by NUPACK software. (B) The structures of the constituents after subjection of T_1_ and their populations predicted by NUPACK software. (C) The structures of the constituents after subjection of T_2_ and their populations predicted by NUPACK software. NUPACK software calculation URL: https://alpha.nupack.org

**Polyacrylamide gel electrophoresis (PAGE) characterization of the formation of CDN system and the conversion of CDN “S” to “X” or “Y”.**

To demonstrate the assembly of each constituent in CDN “S”, we used a classical native polyacrylamide gel electrophoresis (PAGE) experiment. The experimental results are shown in Figure S2A. Lanes 1-3 associated with assembly of aa' constituent that is generated by the successful hybridization of a and a'; lanes 4-6 associated with assembly of bb' constituent that is generated by the successful hybridization of b and b'; Lanes 7-9 associated with assembly of ab' constituent that is generated by the successful hybridization of a and b'; Lanes 10-12 associated with assembly of ba' constituent that is generated by the successful hybridization of b and a'. According to the position of each set of lanes, these results confirmed the intact assembly of the four constituents, aa' bb' ab' and ba'.

To demonstrate the assembly of each constituent in the conversion of CDN “S” to CDN “X” or CDN “Y”, we used a classical native polyacrylamide gel electrophoresis experiment. The experimental results are shown in Figure S2B. Lanes 1-4 correspond to the individual constituents of the CDN “S”, ab', aa', bb' and ba'. Lane 5 is the separated constituents of the parent CDN “S”. The band in lane 6 corresponded to the individual constituent aa'-T_2_ as a reference. Lane 7 showed the bands corresponding to CDN “Y” generated from CDN “S” that is treated to trigger T_2_. As show, the bands corresponding to aa'- T_2_ and bb' are highly bright, while the bands corresponding to the constituents ba' and ab' are barely invisible. Lane 8 showed the band corresponding to the individual constituent ba'- T_1_. Lane 9 showed the bands corresponding to CDN “X” generated from CDN “S” that is treated to trigger T_1_. Also, the bands corresponding to ba'- T_1_ and ab' are highly bright, while the bands corresponding to the constituents aa' and bb' are barely invisible. According to the position and brightness of each set of lanes, these results confirmed the conversion of CDN “S” to CDN “X” or CDN “Y”. It should be noted, to produce a clear separation of the four constituents associated with CDN “S”, the single-stranded a and b' are extended to modify with a nucleic acid tether, respectively. These tethers might affect on the equilibrium of the CDN system. However, the modification tether (60 nt to a, 36 nt to b') are necessary to separate the four constituents, aa', ab', ba' and bb'. The sequences of a_mod_ and b'_mod_ are detailed in the list of nucleic acids. The CDN “S” consists of the constituents aa', ab', ba' and bb' (1 μM each). The individual constituents aa', ab', ba', bb', ba'- T_1_ and aa'- T_2_ are at identical concentration, 1.0 µM.


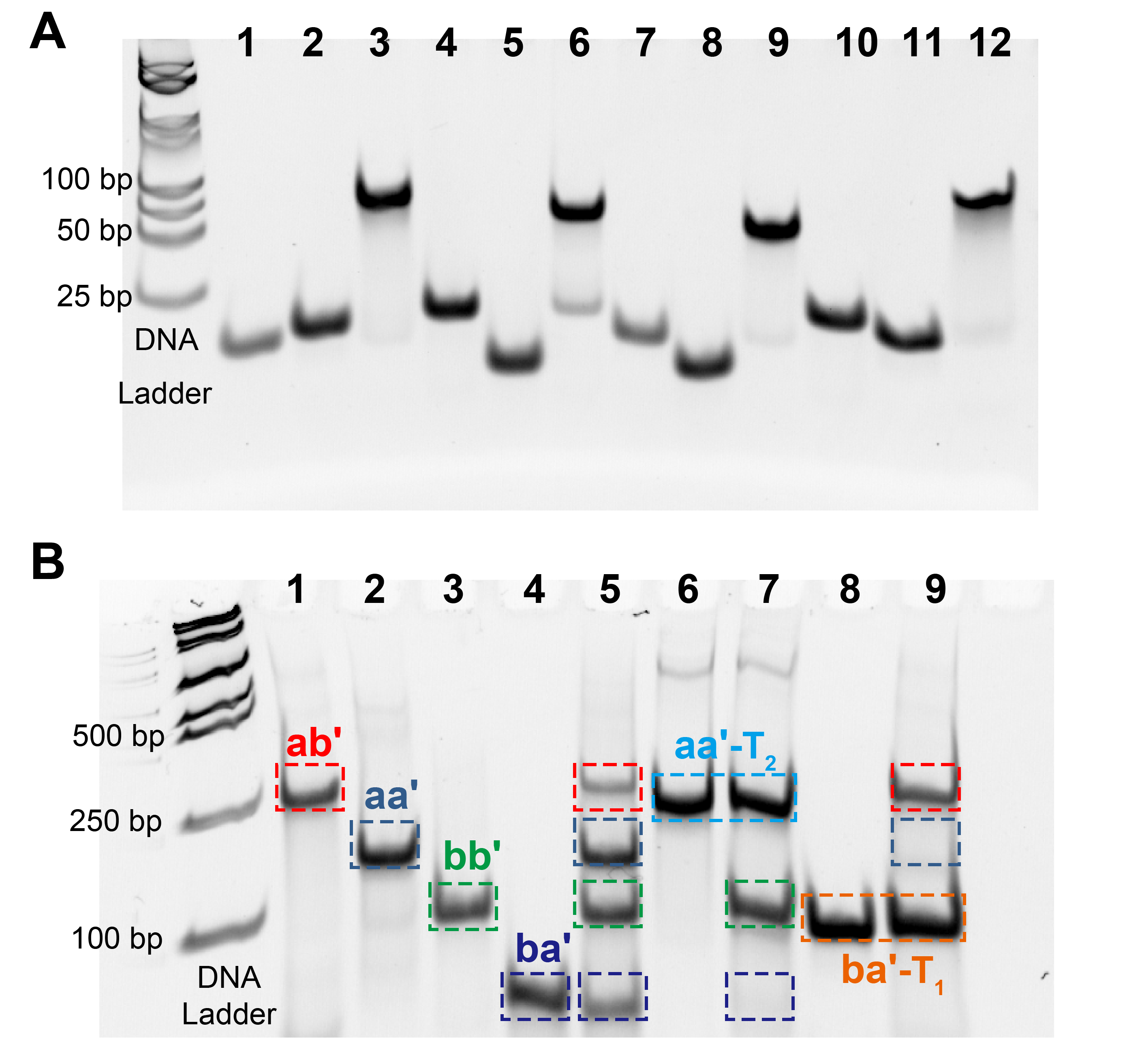


**Figure S2.** (A) Electrophoretic separation of the intact constituents associated with CDN (native PAGE). DNA ladder: 25 bp - 500 bp; Lane 1, a; lane 2, a'; lane 3, aa'; lane 4, b; lane 5, b'; lane 6, bb'; lane 7, a; lane 8, b'; lane 9, ab'; lane 10, b; lane 11, a'; lane 12, ba'. The concentration of single-stranded DNA in each lane is 1 μM, and the concentration of the duplex is 1 μM. Acrylamide (10%), gel thickness, 1 mm. All samples are separated upon applying a potential of 200 V, under ice bath. The separation of the samples is conducted for a time-interval of 1 hour. (B) Electrophoretic separation of the constituents associated with the conversion of CDN “S” to CDN “X” or CDN “Y” (native PAGE). DNA ladder: 100 bp – 5,000 bp; Lane 1, ab'; lane 2, aa'; lane 3, bb'; lane 4, ba'; lane 5, CDN “S”; lane 6, aa'- T_2_; lane 7, CDN “Y”; lane 8, ba'- T_1_; lane 9, CDN “X”. Lanes 1−4, 6 and 8 corresponded to the individual constituents, 1 μM each (Note that modified a, termed as a_mod_, and modified b', termed as b'_mod_, are used to separate the bands of four constituents within the CDN). Acrylamide (20%), gel thickness, 1 mm. Mixtures are separated upon applying a potential of 200 V, under ice bath. The separation of the samples is conducted for a time-interval of 1.5 hour following by staining the gel by Gel Red solution (3 ×) for 15 min.

**Calibration curves of CDN constituents (HITACHI F7100 Fluorometer)**


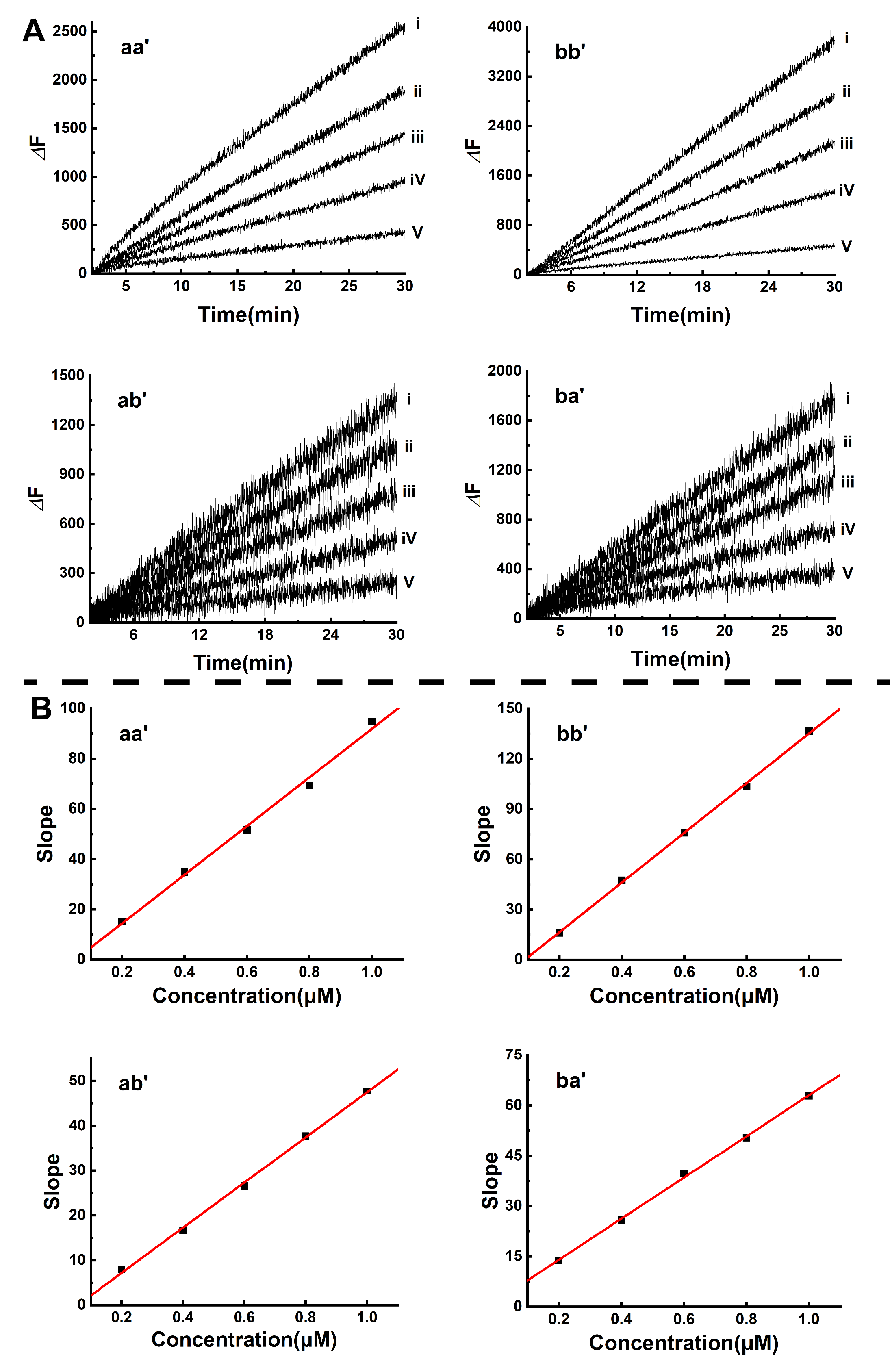


**Figure S3.** (A) Time-dependent fluorescence changes generated upon the cleavage of the fluorophore/quencher-modified substrates by the respective Mg^2+^-ion-dependent DNAzyme reporter units associated with the individual constituents at variable concentrations: (i) 1 μM, (ii) 0.8 μM, (iii) 0.6 μM, (iv) 0.4 μM, and (v) 0.2 μM. (B) Corresponding calibration curves of the catalytic rates of the different constituents as a function of their concentrations, derived from the data shown in Figure S3A.

**Preliminary experiments: Time-dependent fluorescence verification of CDN conversions**


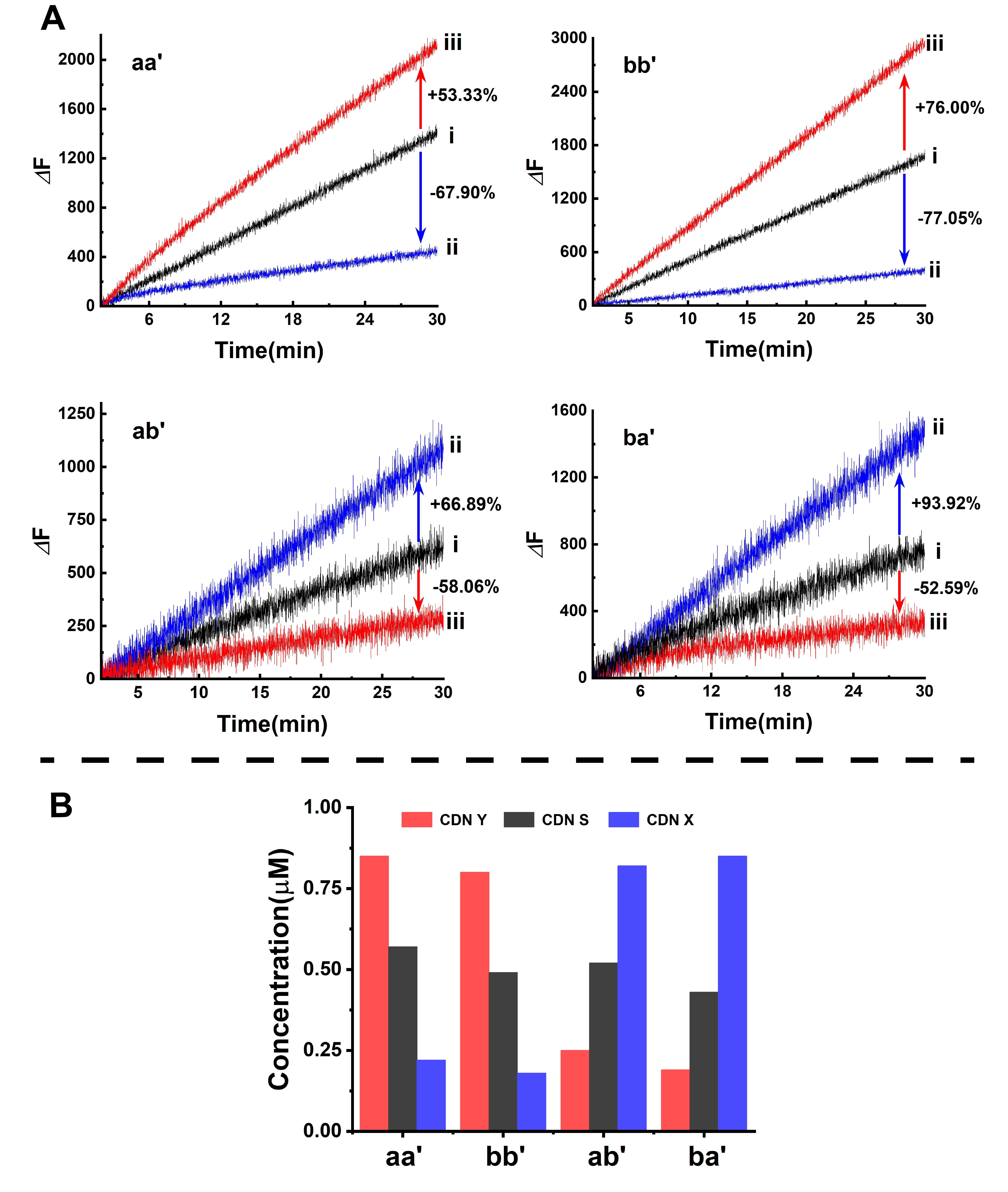


**Figure S4.** (A) Time-dependent fluorescence changes generated by DNAzyme reporter units associated with four constituents of the CDNs: (i) CDN “S”, in the absence of $\text{T}_{\text{1}}$ and $\text{T}_{\text{2}}$, (ii) CDN “X”, in the presence of 1 μM $\text{T}_{\text{1}}$, and (iii) CDN “Y”, in the presence of 1 μM $\text{T}_{\text{2}}$. (B) Concentrations of the constituents in CDNs converted by the $\text{T}_{\text{1}}$ or $\text{T}_{\text{2}}$. The concentrations of the constituents are determined by the time-dependent fluorescence changes generated by the DNAzyme reporter units and using appropriate calibration curves in Figures S3.

**Calibration curves of CDN constituents (Agilent Cary Eclipse Fluorometer)**


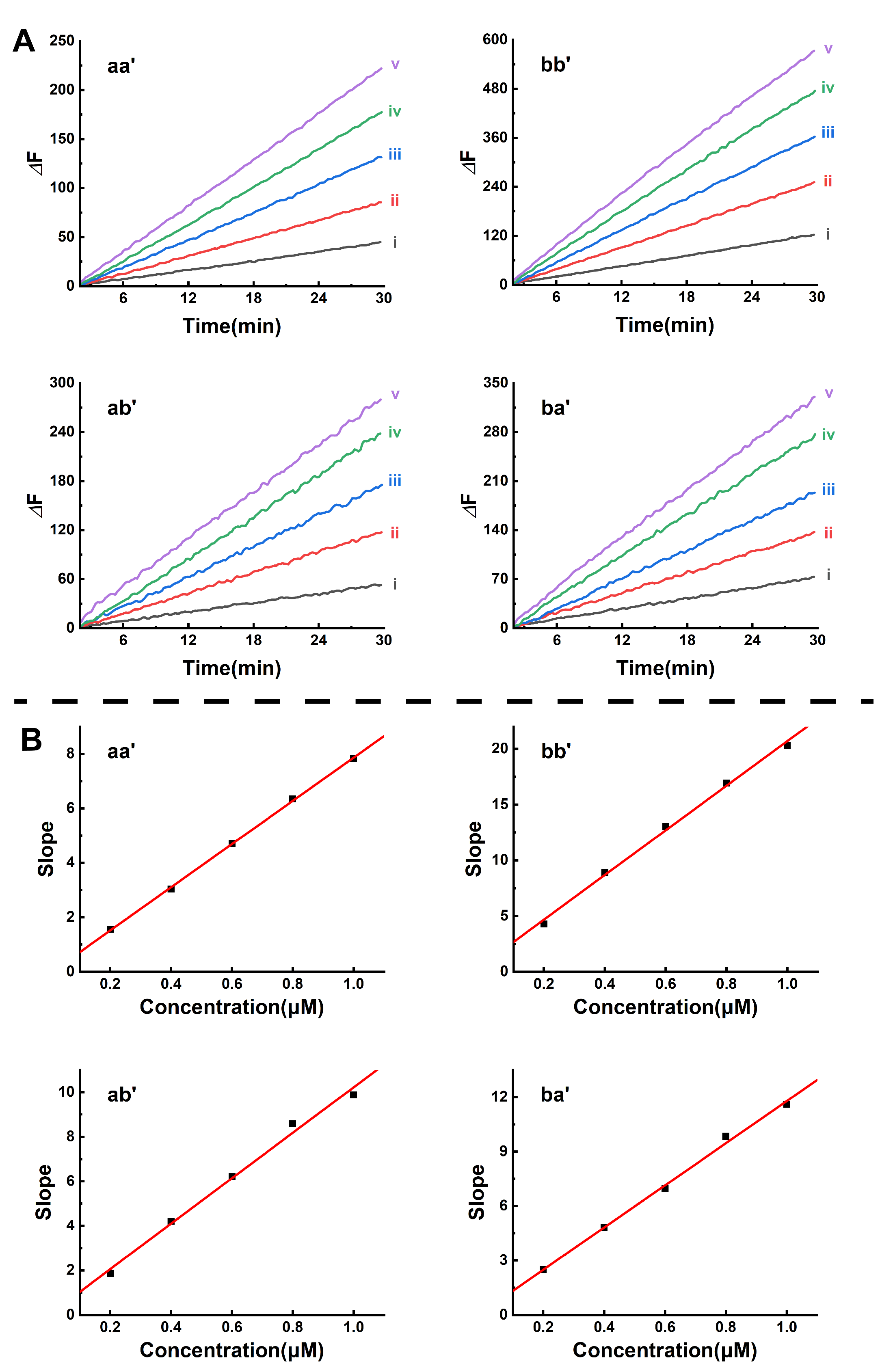


**Figure S5.** (A) Time-dependent fluorescence changes generated upon the cleavage of the fluorophore/quencher-modified substrates by the respective Mg^2+^-ion-dependent DNAzyme reporter units associated with the individual intact constituents at variable concentrations: (i) 0.2 μM, (ii) 0.4 μM, (iii) 0.6 μM, (iv) 0.8 μM, (v) 1 μM. (B) Corresponding calibration curves of the catalytic rates of the different constituents as a function of their concentrations, derived from the data shown in Figure S5A.

**Photo-triggered conversion of equilibrated CDN “S” to CDN** **“Y”**

Figure S6 schematically depicts the light-triggered emergence of a constitutional dynamic network, the CDN “S”, that cannot be regulated by T_2_ in the absence of light due to the fully complementary of T_2_ to T_2_'. The T_2_' strand, functionalized with few o-nitrobenzyl phosphate photoresponsive moieties (PC linker), undergoes photo-deprotection upon irradiation at *λ* = 365 nm. The photo-cleaved T_2_' is digested from T_2_/T_2_' duplex, releasing T_2_ to bind to the residual tether associated to aa' and allowing the transition of CDN from “S” to “Y”. The corresponding up-regulation or down-regulation of the constituents are marked with arrow. Each of the constituents includes a Mg^2+^-ion-dependent DNAzyme unit. The Mg^2+^-ion-dependent DNAzymes act as reporter units to quantitatively monitor the concentrations of the respective constituents.

Figure S7 depicts the fluorescent changes of the four constituents of the CDN system before and after light exposure by the DNAzyme units. Specifically, in Figure S7A, curve (i) corresponds to the signal of the DNAzyme unit of the four constituents before light activation, and the curve (ii) is the signal of the DNAzyme reporting unit of the four constituents after light activation. Upon light stimulation, the concentrations of aa' and bb' are significantly up-regulated, while the concentrations of ab' and ba' are down-regulated, which is concomitant with the transition from CDN “S” to CDN “Y”. Figure S7B presents the histogram derived from the conversion of the signals in Figure S7A into corresponding concentration values. The black bars represent the concentrations of the four constituents prior to light exposure, while the red bars indicate the concentrations of the same constituents following light irradiation.

**
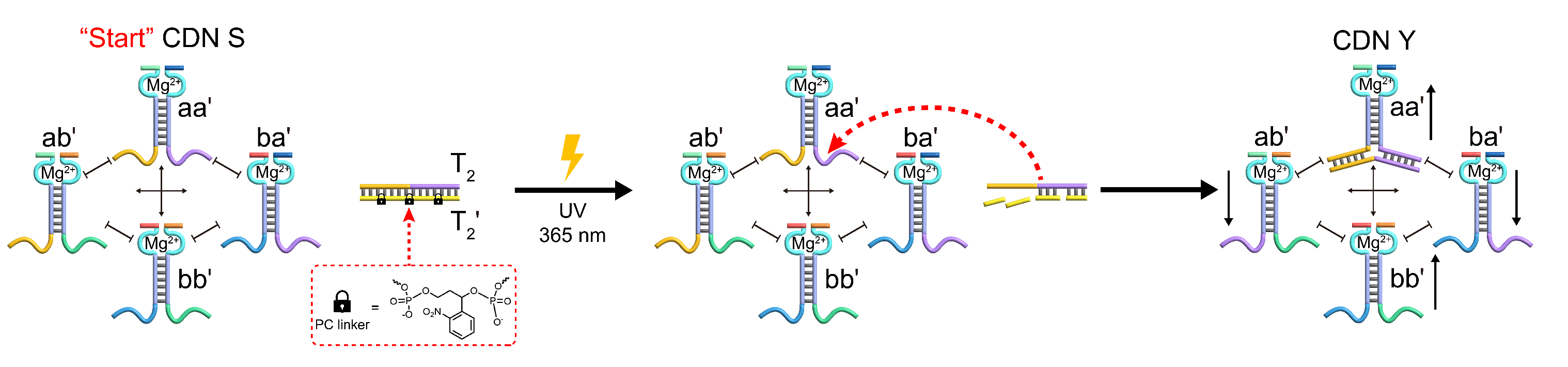
**

**Figure S6.** Schematic illustration of light-triggered reconfiguration of CDN “S” to CDN “Y”. The photo-cleaved T_2_' are digested from T_2_/T_2_' duplex, releasing T_2_ to bind to the residual tether associated to aa' and allowing the transition of CDN system from “S” to “Y”.

**
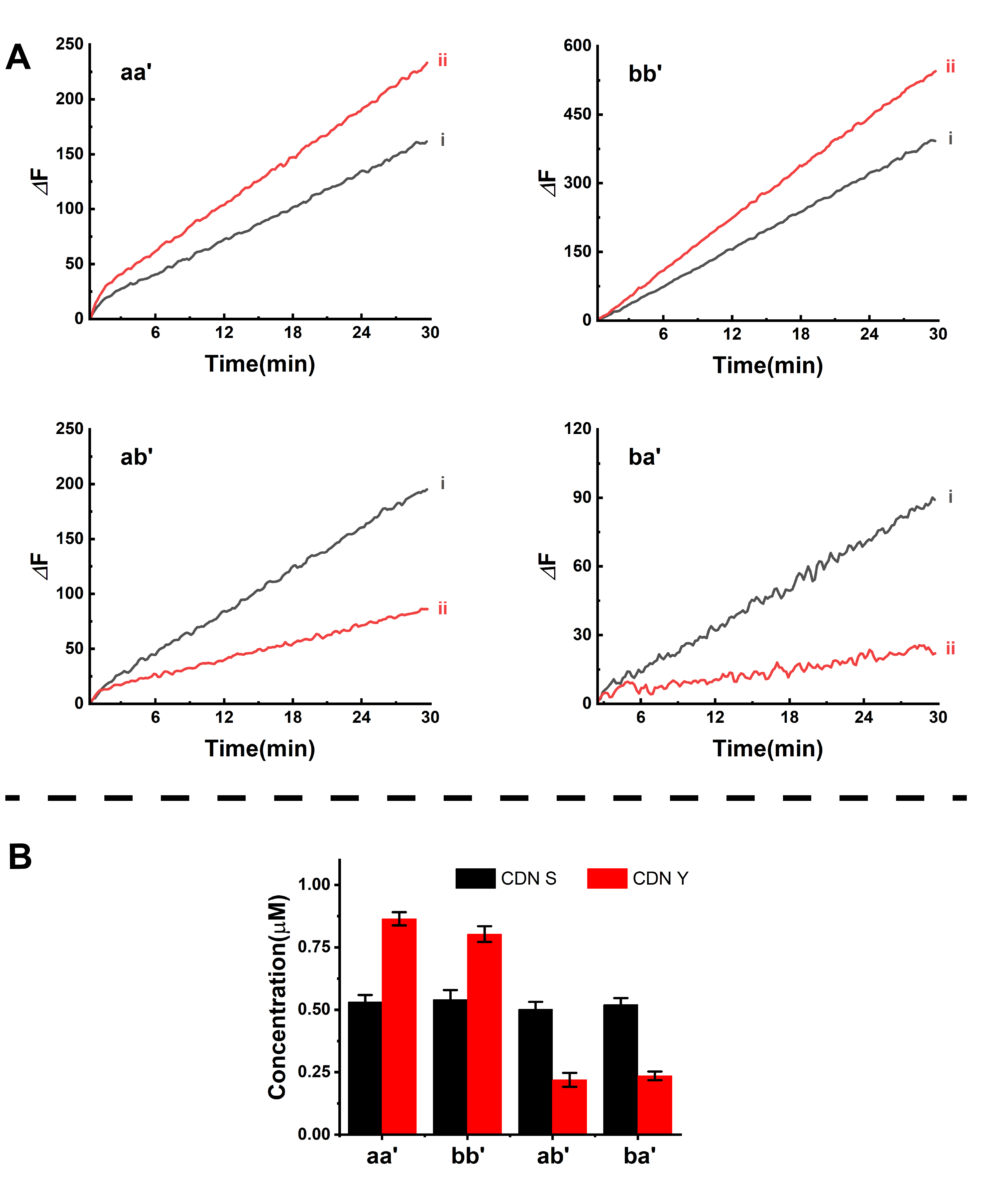
**

**Figure S7.** (A) Time-dependent fluorescence changes generated by DNAzyme reporter units associated with four constituents of the CDNs: (i) CDN “S” without exposure to a light source, (ii) CDN “Y” following 5 minutes of exposure to a light source. (B) Bar presentation of the concentrations associated to the constituents in CDN “S” converted to CDN “Y”. The concentrations of the constituents are determined by the time-dependent fluorescence changes generated by the DNAzyme reporter units and using appropriate calibration curves in Figures S5. Error bars represent mean ± SD, n = 3.

**Time-dependent fluorescence of adaptive defined-threshold associated with CDN system**

**
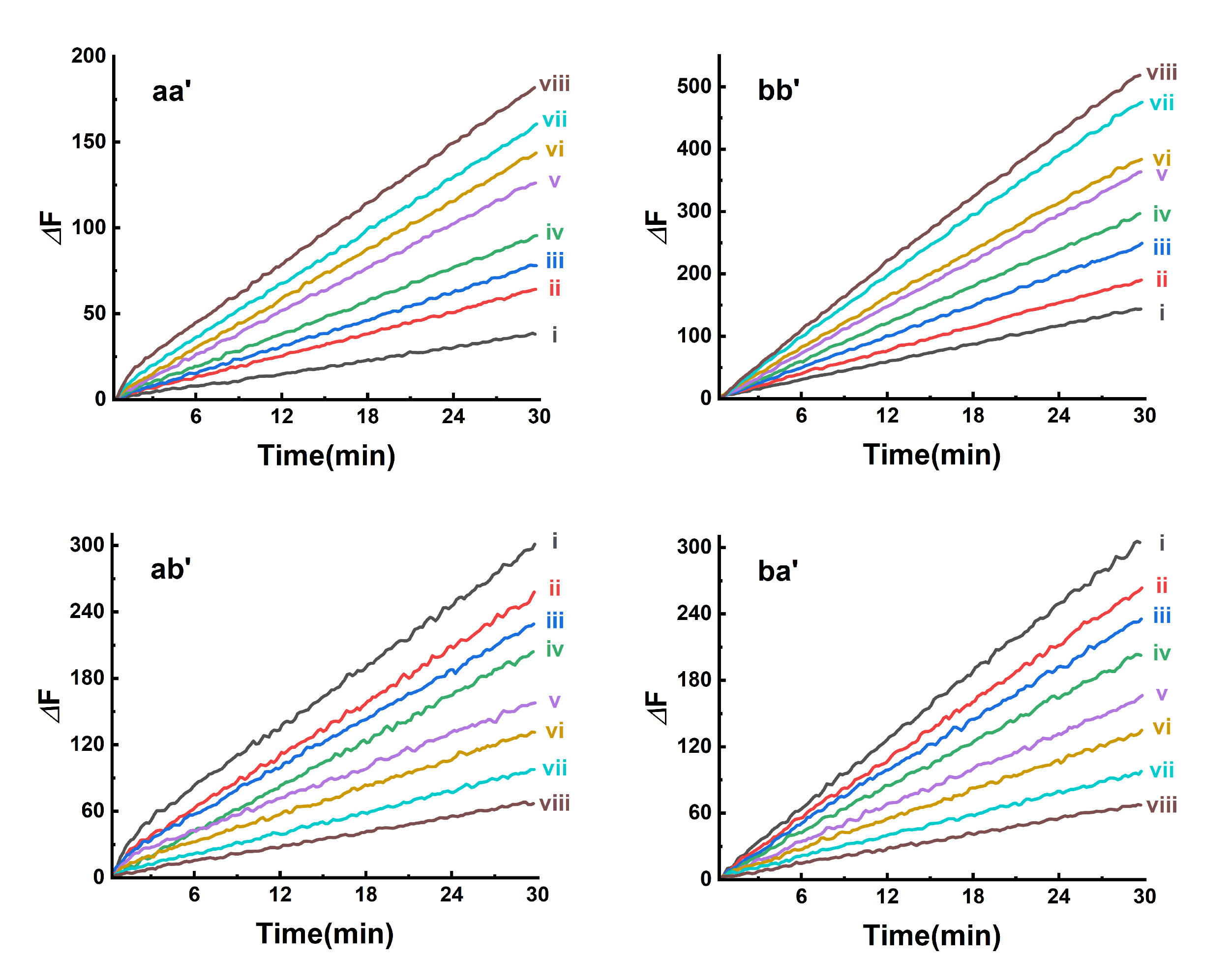
**

**Figure S8.** Time-dependent fluorescence changes generated by DNAzyme reporter units associated with four constituents of the CDNs for adaptive defined threshold: (i) 1.0 μM T_1_, (ii) 0.75 μM T_1_, (iii) 0.5 μM T_1_, (iv) 0.25 μM T_1_, (v) 0.25 μM T_2_, (vi) 0.5 μM T_2_, (vii) 0.75 μM T_2_, (viii) 1.0 μM T_2_.

**The kinetics simulation of CDN “S” converted to CDN “X” or CDN “Y”**

Figure 2D showed time-dependent concentration changes of different constituents from CDN “S” to CDN “X”, the specific concentrations of four constituents from the corresponding calibration curves are shown in the dots, and the simulation calculation data is shown by solid line. The kinetic reactions associated with the time-dependent concentration changes of the constituents of CDN “X” upon subjecting the CDN “S” to the trigger strand T_1_ that shifts the equilibrium of the constituents in CDN “X” is summarized in equations (1) to (8). When CDN “S” is treated with trigger strand T_1_, the concentrations of ab' and ba' showed a robust increase in fluorescence after only 15 min of incubation at 37 ℃. And the concentration changes of the two constituents followed the first order reaction. The concentrations of aa' and bb' decreased accordingly.

Figure 2E showed time-dependent concentration changes of different constituents from CDN “S” to CDN “Y”, the specific concentrations of four constituents from the corresponding calibration curves are shown in the dots, and the simulation calculation data is shown by solid line. The kinetic reactions associated with the time-dependent concentration changes of the constituents of CDN “Y” upon subjecting the CDN “S” to the trigger strand T_2_ that shifts the equilibration of the constituents in CDN “Y” is summarized in equations (9) to (16). When CDN “S” is treated with trigger strand T_2_, the concentrations of aa' and bb' showed a robust increase in fluorescence after only 15 min of incubation at 37 ℃. And the concentration changes of the two constituents followed the first order reaction. The concentrations of ab' and ba' decreased accordingly. The experimental data points are obtained by time-dependent fluorescence at 25 °C for 30 min, the concentrations of the constituents are determined by the time-dependent fluorescence changes generated by the DNAzyme reporter units and using appropriate calibration curves in Figure S3 and S5.

**Table S1.** Rate constants of equations (1) ~ (8) (constituents exchanging reaction within the network) derived from the computational simulation of the transition of CDN “S” from CDN “X”.

| $\boldsymbol{k}_{\boldsymbol{1}}$ | 0.30724 μM^-1^min^-1^ | $\boldsymbol{k}_{\boldsymbol{2}}$ | 9.78014 μM^-1^min^-1^ |
| --- | --- | --- | --- |
| $\boldsymbol{k}_{\boldsymbol{-1}}$ | 0.23265 μM^-1^min^-1^ | $\boldsymbol{k}_{\boldsymbol{-2}}$ | 0.04500 min^-1^ |

**Table S2.** Rate constants of equations (9) ~ (16) (constituents exchanging reaction within the network) derived from the computational simulation of the transition of CDN “S” to CDN “Y”.

| $\boldsymbol{k}_{\boldsymbol{1}}$ | 0.30724 μM^-1^min^-1^ | $\boldsymbol{k}_{\boldsymbol{3}}$ | 11.69853 μM^-1^min^-1^ |
| --- | --- | --- | --- |
| $\boldsymbol{k}_{\boldsymbol{-1}}$ | 0.23265 μM^-1^min^-1^ | $\boldsymbol{k}_{\boldsymbol{-3}}$ | 0.73822 min^-1^ |

**Figure S9.** Computational simulation of the transitions of CDN “S” to CDN “X”. The kinetic reactions associated with the time-dependent concentration changes during the transitions of CDN “S” to CDN “X” is summarized in equations (1) to (8). Knowing the time-dependent concentration changes of the CDNs constituents during the transitions of CDN “S” to CDN “X”, we computationally simulated the time-dependent concentration changes of the constituents by using MATLAB R2022b.

**Figure S10.** Computational simulation of the transitions of CDN “S” to CDN “Y”. The kinetic reactions associated with the time-dependent concentration changes during the transitions of CDN “S” to CDN “Y” is summarized in in equations (9) to (16). Knowing the time-dependent concentration changes of the CDNs constituents during the transitions of CDN “S” to CDN “Y”, we computationally simulated the time-dependent concentration changes of the constituents by using MATLAB R2022b.


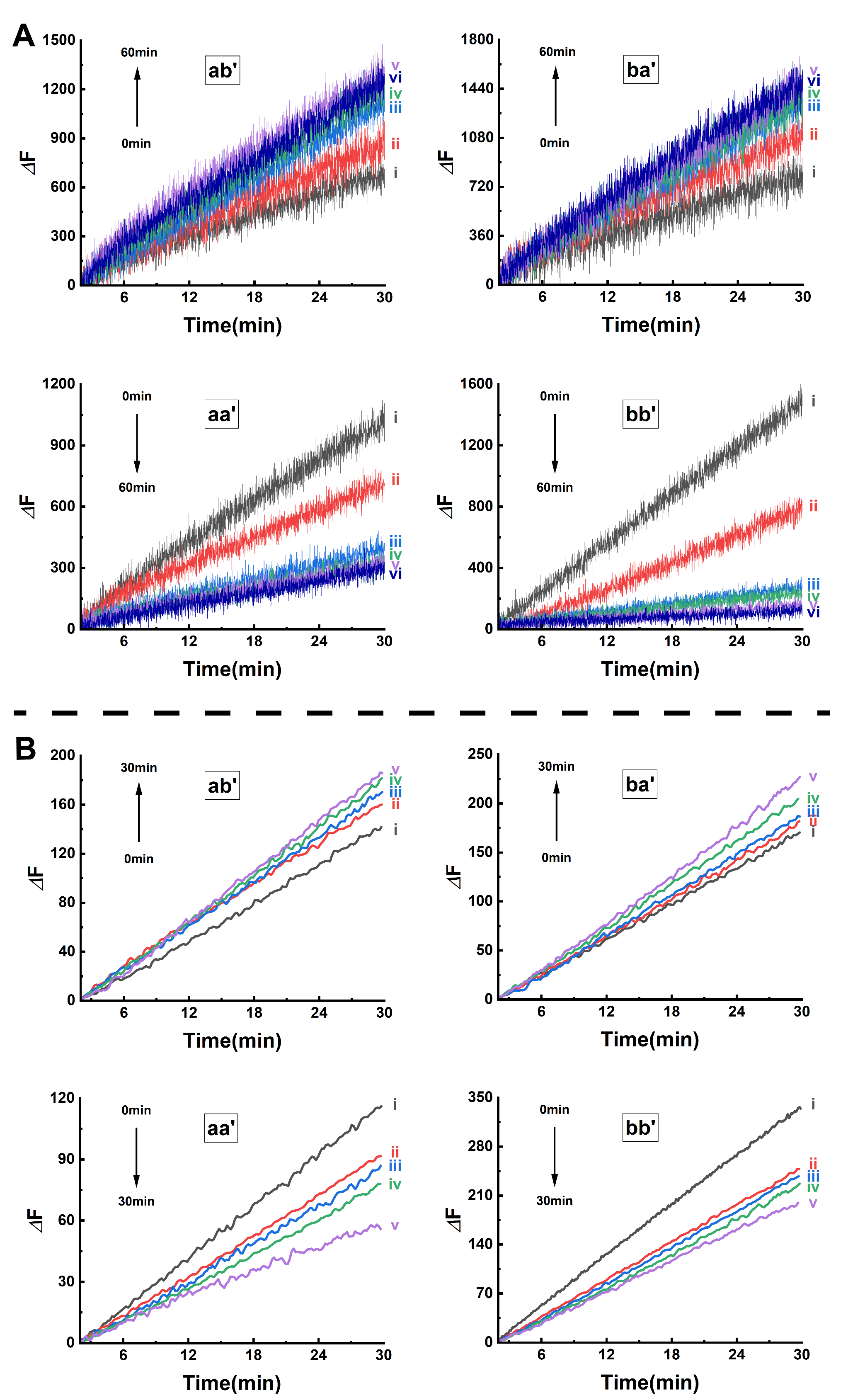


**Figure S11.** (A) Time-dependent fluorescence changes generated by DNAzyme reporter units coupled to the constituents of CDN “X” upon subjecting the CDN “S” to the trigger T_1_, 1.0 μM. (i) 0 min, (ii) 5 min, (iii) 10 min, (iv) 15 min, (v) 30 min, (vi) 60 min. (B) Time-dependent fluorescence changes generated by DNAzyme reporter units coupled to the constituents of CDN “X” upon subjecting the CDN “S” to the trigger T_1_, 0.5 μM. The fluorescent measurement was monitored under the incubation of CDN “S” to T_1_ after different time-intervals: (i) 0 min, (ii) 5 min, (iii) 10 min, (iv) 15 min, (v) 30 min.

Note that the fluorescence response of Figure S13B is recorded by Cary Eclipse Fluorometer.


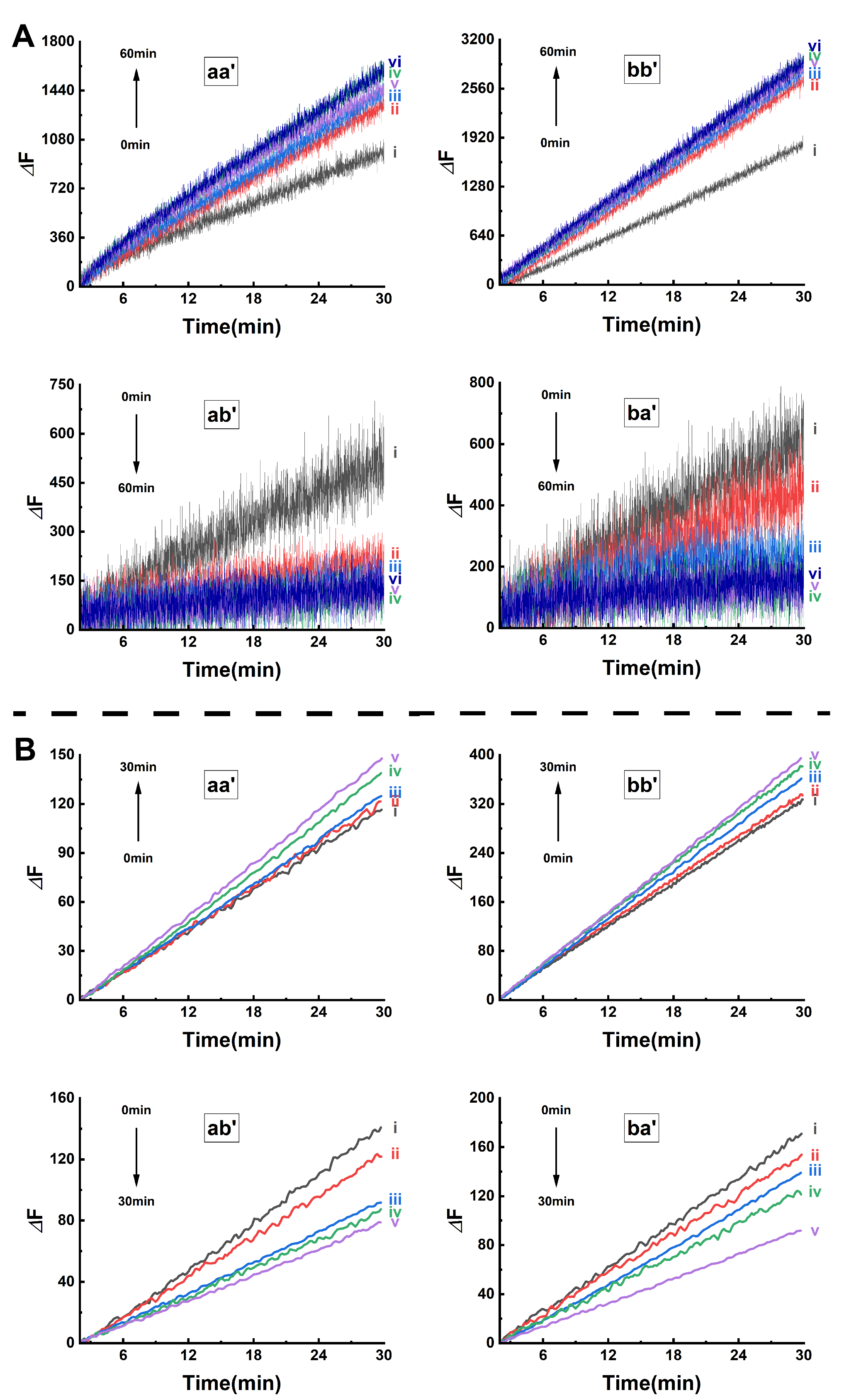


**Figure S12.** (A) Time-dependent fluorescence changes generated by DNAzyme reporter units coupled to the constituents of CDN “Y” upon subjecting the CDN “S” to the trigger T_2_, 1.0 μM. (i) 0 min, (ii) 5 min, (iii) 10 min, (iv) 15 min, (v) 30 min, (vi) 60 min. (B) Time-dependent fluorescence changes generated by DNAzyme reporter units coupled to the constituents of CDN “Y” upon subjecting the CDN “S” to the trigger T_2_, 0.5 μM. The fluorescent measurement was monitored under the incubation of CDN “S” to T_2_ after different time-intervals: (i) 0 min, (ii) 5 min, (iii) 10 min, (iv) 15 min, (v) 30 min.

Note that the fluorescence response of Figure S14B is recorded by Cary Eclipse Fluorometer.

**The reconfiguration of CDN “S” to CDN “X” triggered by 0.75 μM T_1_ and corresponding simulation**

**
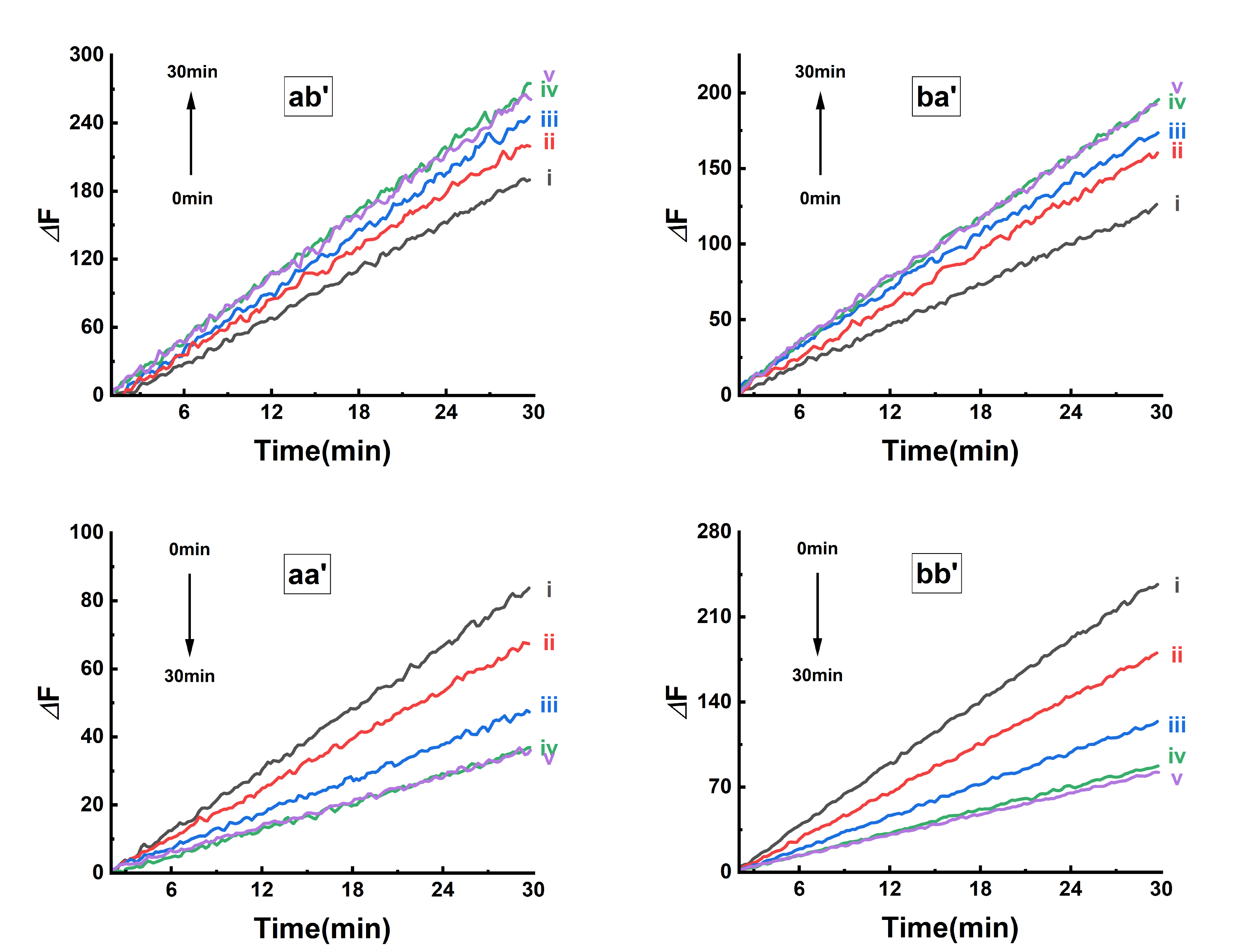
**

**Figure S13.** Time-dependent fluorescence changes generated by DNAzyme reporter units coupled to the constituents of CDN “X” upon subjecting the CDN “S” to the trigger T_1_, 0.75 μM. The fluorescent measurement is monitored under the incubation of CDN “S” to T_1_ after different time-intervals: (i) 0 min, (ii) 5 min, (iii) 10 min, (iv) 15 min, (v) 30 min.

**
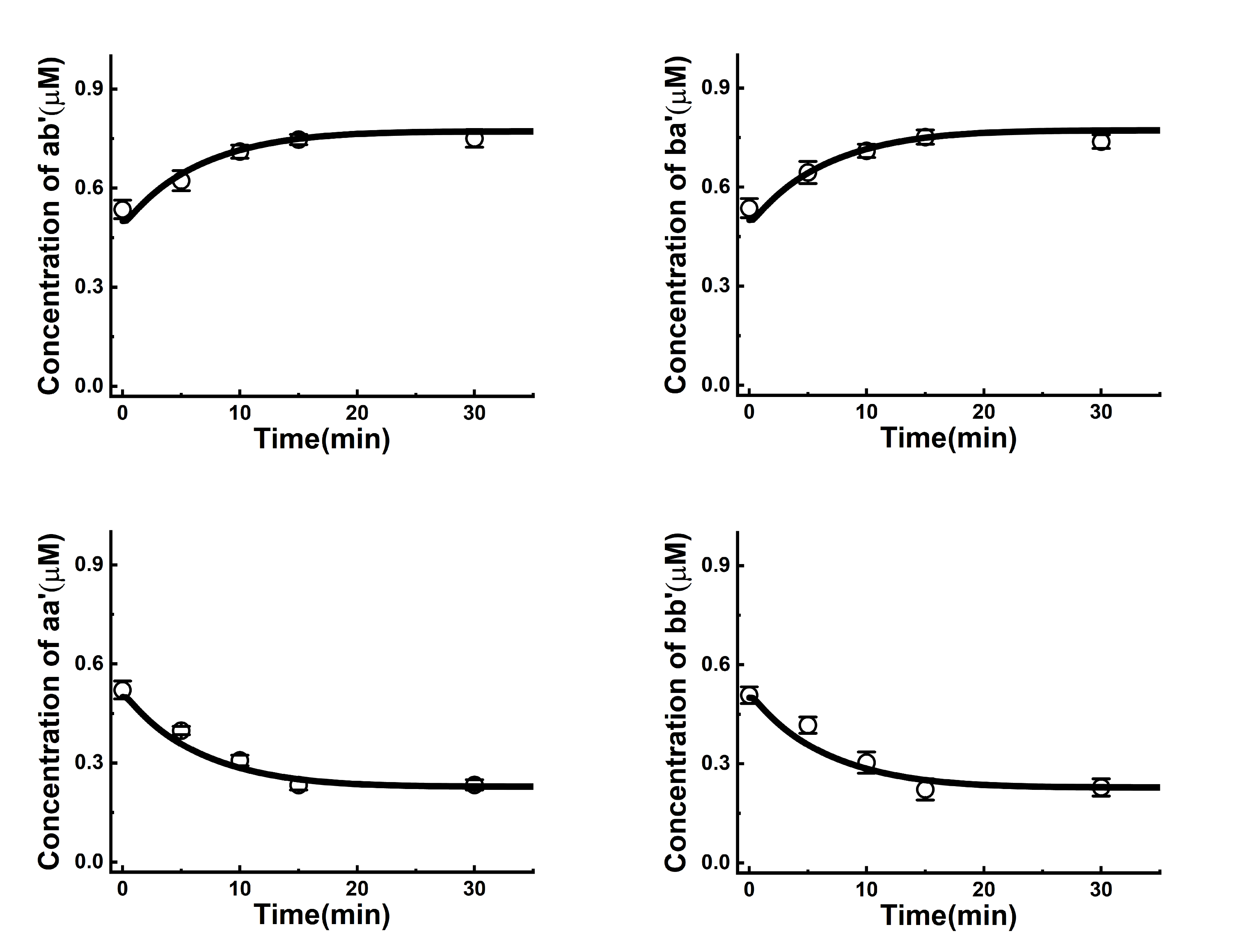
**

**Figure S14.** Temporal concentration changes of different constituents of 0.75 μM T_1_-triggered reconfiguration of CDN “S” to CDN “X”. (Experimental data: points; Computational simulation: solid lines. The computationally simulated concentration changes are based on the kinetic model formulated in Figure S9). Error bars represent mean ± SD, n = 3.

**Binary translation based on individual aa'-based constituent**

The two sets of original experimental data for the confusion matrix analysis in Figure 2 on individual aa' DNAzyme and binary digital translation is depicted in Figure S15 (set 1) and S16 (set 2).


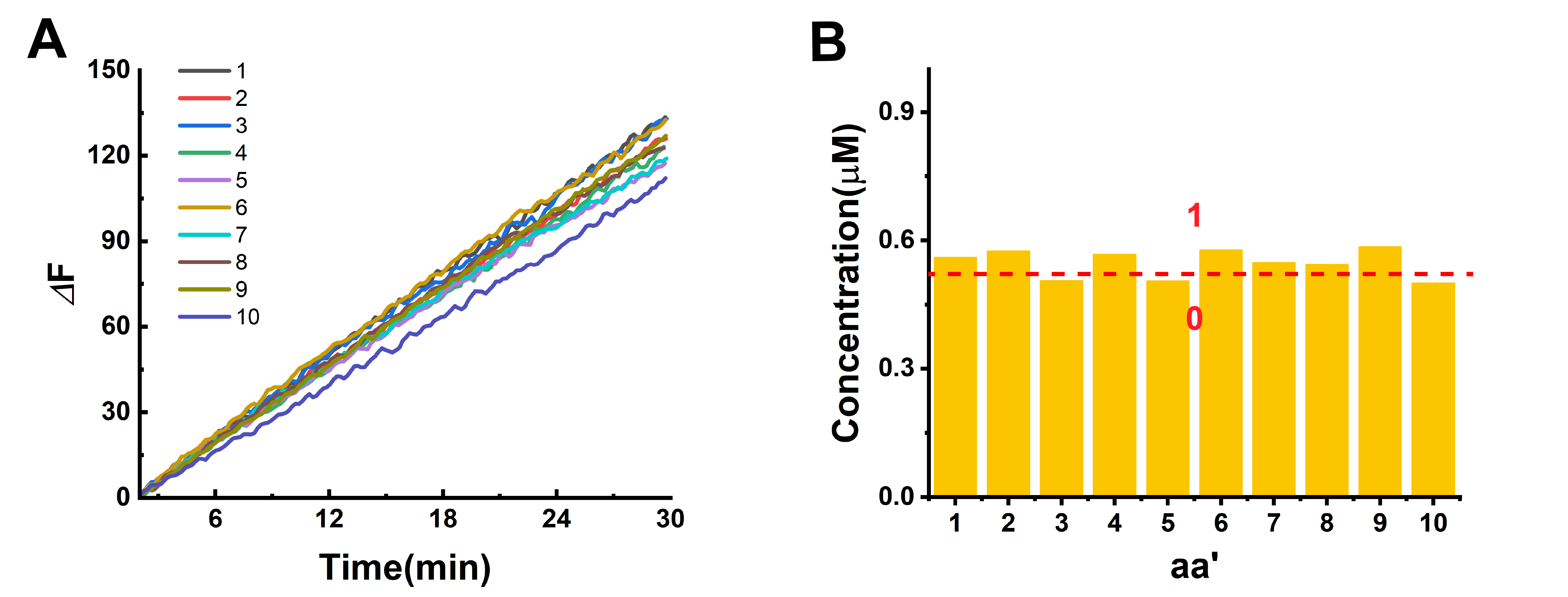


**Figure S15.** (A) Time-dependent fluorescence changes generated by independent aa'-DNAzyme under 10 parallel experiments, set 1. (B) The concentrations of aa' in the form of a bar presentation. The concentrations of the aa' is determined by the time-dependent fluorescence changes generated by the DNAzyme reporter unit and using appropriate calibration curve in Figure S5. The red dotted line is the fixed threshold.


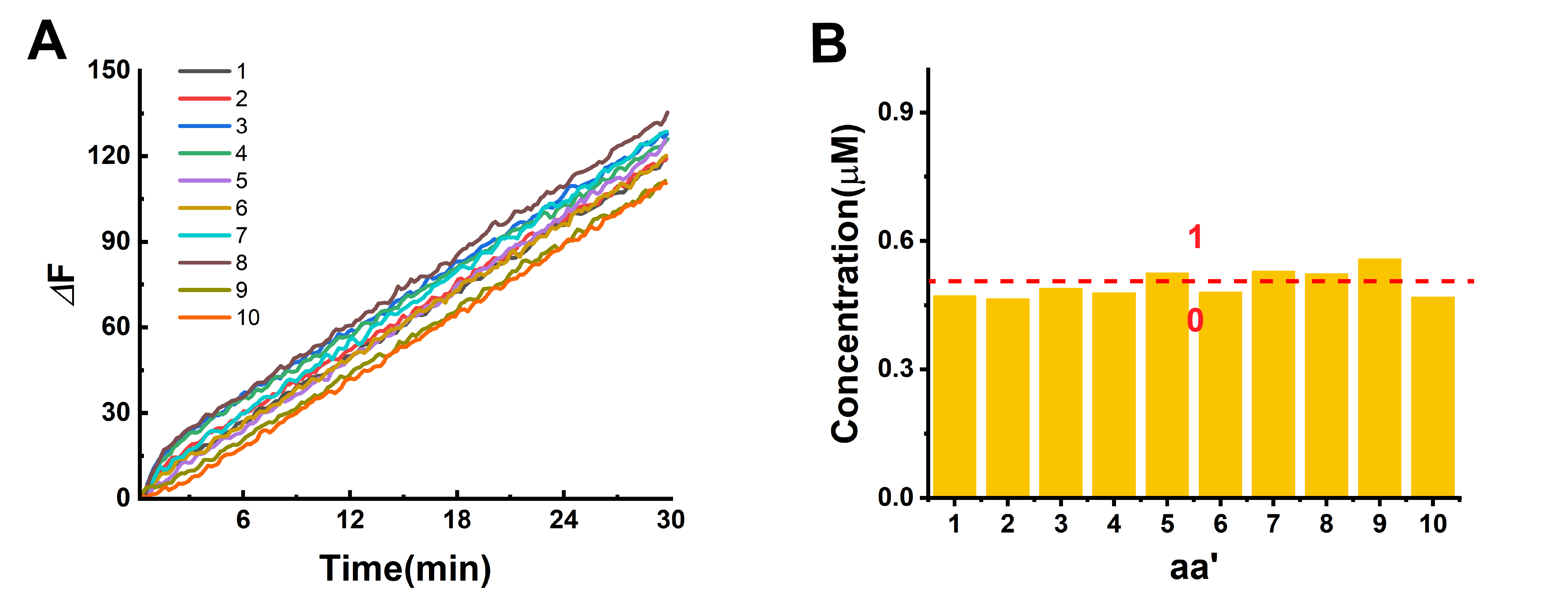


**Figure S16.** (A) Time-dependent fluorescence changes generated by DNAzyme reporter unit associated with constituent of the aa' under 10 times experiments, set 2. (B) The concentrations of aa' in the form of a bar presentation. The concentrations of the aa' is determined by the time-dependent fluorescence changes generated by the DNAzyme reporter unit and using appropriate calibration curve in Figure S5. The red dotted line is the fixed threshold.

**Selectivity of the CDN system** **towards the external stimuli DNA**

Figure S17 showed the selectivity corresponding to the CDN system. We used two irrelevant miRNAs (miRNA-21, miRNA-221) as the interferences to assess the selectivity of the CDN system. Negligible time-dependent fluorescence changes are obtained in response to miRNA-21or miRNA-221, the two constituents (bb' and ab') that corresponding to the up-regulate or down-regulate in response in the CDN “X” or CDN “Y” state show essentially the same concentration as in the CDN “S” state, suggesting a good selectivity of the CDN system.


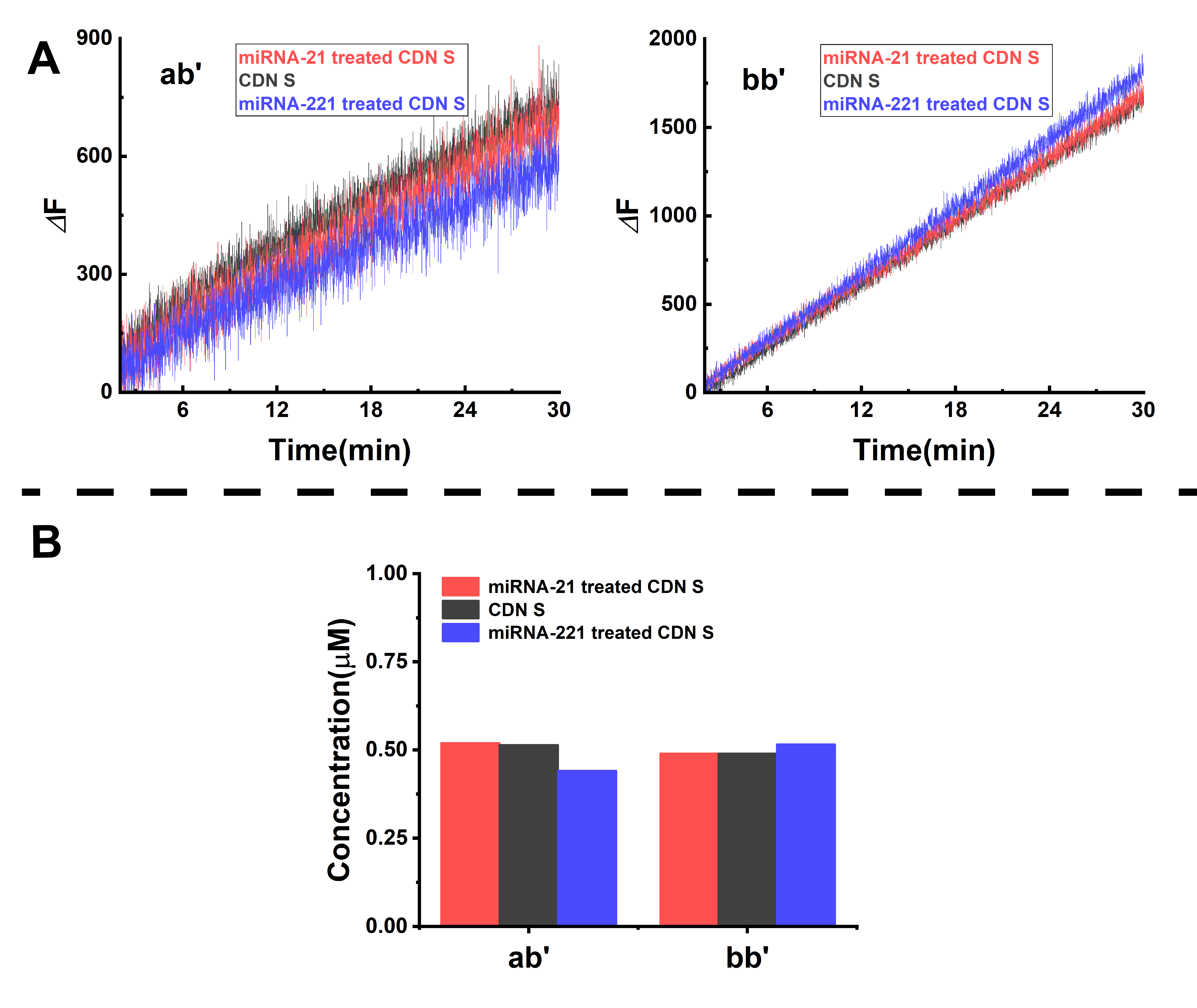


**Figure S17.** Selectivity of the CDNs system. (A) Time-dependent fluorescence changes generated by DNAzyme reporter units associated with ab' and bb'. (B) The concentrations of ab' and bb' in the form of a bar presentation. The concentrations of the constituents are determined by the time-dependent fluorescence changes generated by the DNAzyme reporter units and using appropriate calibration curves in Figures S3.

**The combined subjection of T_1_ and T_2_ to CDN system**

When both T_1_ and T_2_ are introduced into the CDN system, the transition behavior of the system is determined by the relative concentrations of the triggers, T_1_ and T_2_, which compete to regulate the constituent components. As illustrated in Figure 2A, trigger T_1_ facilitates the transition of the CDN from state “S” to state “X” through the up-regulation of ba'. Conversely, when the concentration of T_2_ exceeds that of T_1_, the system tends to transition into state “Y” due to the up-regulation of aa' induced by T_2_. To investigate this behavior, experiments were conducted by maintaining the concentration of T_1_ at a constant level of 0.5 μM, while varying the concentration of T_2_ at 0.1 μM and 1.0 μM.

As depicted in Figure S20 and S21, the concentration of T_1_ is held constant at 0.5 μM. When the concentration of T_2_ (0.1 μM) is lower than that of T_1_, the competitive interaction between T_1_ and T_2_ results in the dominance of T_1_, leading to an increase in the concentrations of ab' and ba'. Conversely, the concentrations of aa' and bb' decrease due to the constraint of the constant total concentration of the four constituents. In contrast, when the concentration of T_2_ (1.0 μM) exceeds that of T_1_, the opposite trend is observed. When the concentrations of T_2_ (0.5 μM) and T_1_ are equal, the competitive effects of the two triggers essentially neutralize each other, and the concentrations of the four constituents remain approximately consistent with those observed in the initial state of CDN “S”. In the absence of both T_1_ and T_2_ in the system, referred to as CDN "S", the concentrations of the four constituents, aa', ab', ba', and bb', are designed to be equal, as depicted in Figure S4. The CDN "S" system does not exhibit any threshold segmentation, and consequently, no outputs are generated from this state.

**
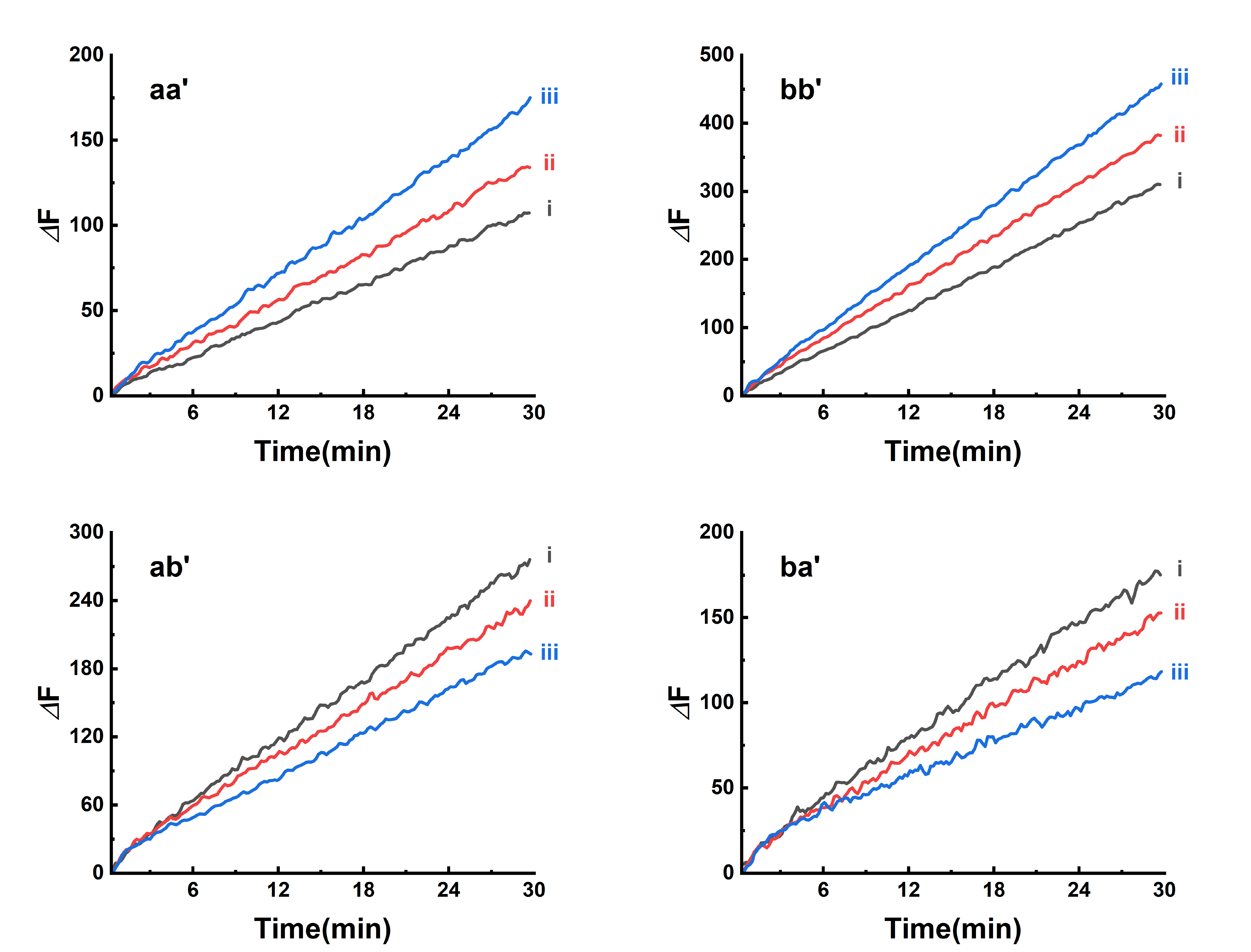
**

**Figure S18.** Time-dependent fluorescence changes generated by DNAzyme reporter units associated with four constituents of the CDNs: (i) CDN “S”, in the presence of 0.5 μM T_1_ and 0.1 μM T_2_, (ii) CDN “S”, in the presence of 0.5 μM T_1_ and 0.5 μM T_2_, (iii) CDN “S”, in the presence of 0.5 μM T_1_ and 1.0 μM T_2_.

**
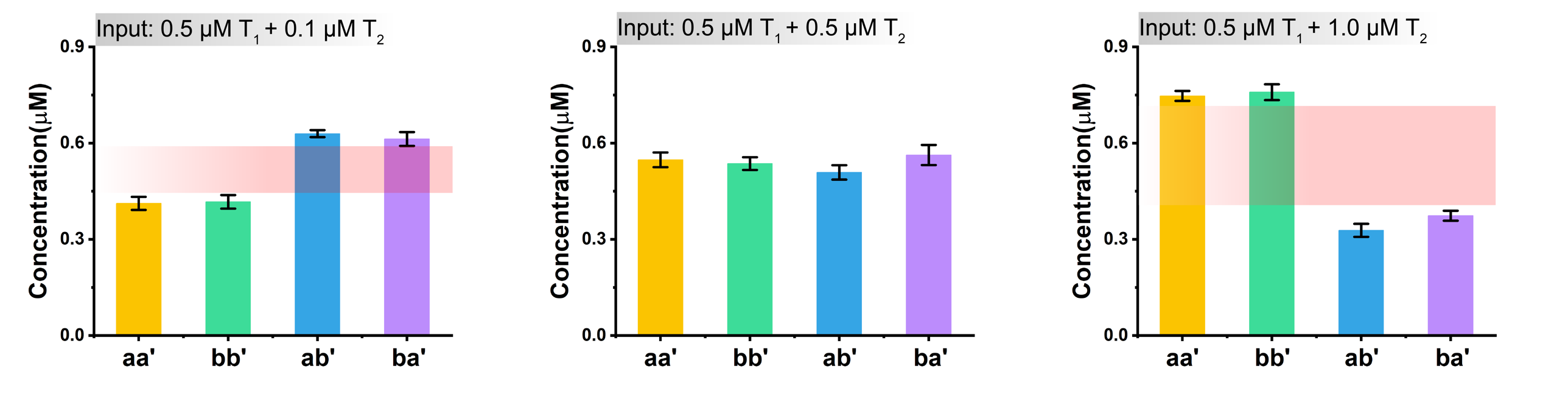
**

**Figure S19.** Bar presentation of the concentrations of different constituents in CDNs converted by the co-existence of T_1_ and T_2_. The concentrations of the constituents are determined by the time-dependent fluorescence changes generated by the DNAzyme reporter units in Figure S5. Error bars represent mean ± SD, n = 3.

**Table S3.** **The comparison between the proposed adaptive method and other DNA-based encryption methods.**

| DNA configuration | Test method | Time complexity | Read-out time | Reference |
| --- | --- | --- | --- | --- |
| DNA hairpin | Fluorometer and spectrophotometer | O(*n*) - Linear Time | ~ 18 min | 5 |
| DNA origami | Atomic force microscope (AFM) | O(*n*) - Linear Time | ~ 40 min | 6 |
| DNA origami | Atomic force microscope (AFM) | O(*n*) - Linear Time | ~ 40 min | 7 |
| DNA duplex | Fluorometer and spectrophotometer | O(*n*) - Linear Time | ~ 15 min | This work |

[5] L. Zheng, J. Li, M. Wen, D. Xi, Y. Zhu, Q. Wei, X. Zhang, G. Ke, F. Xia, Z. Gao, *Sci. Adv.* **2023**, *9,* eadf5868.

[6] S. Fan, D. Wang, J. Cheng, Y. Liu, T. Luo, D. Cui, Y. Ke, J. Song, *Angew. Chem. Int. Ed*. **2020**, *59*, 12991.

[7] Y. Zhang, F. Wang, J. Chao, X. Liu, C. Fan, Q. Li, J. Shi, M. Xie, H. Liu, M. Pan, L. Wang, J. Hu, L. Wang, E. Kopperger, F. C. Simmel, *Nat. Commun.* **2019**, *10*, 5469.

It is noteworthy that these statistical timeframes were roughly estimated and calculated based on the experimental procedures described in the literature.

**Validation of the gold nanoparticles (Au NPs) and the DNA-functionalized Au NPs**

The property of the gold nanoparticles (Au NPs) is characterized by TEM. As shown in Figure S20A, Au NPs showed spherical shape with good dispersity. As shown in Figure S20B, the distribution of Au NPs diameter based on the Figure S20A exhibited a good normal distribution, 14 nm diameter. These characterizations of the Au NPs confirmed that the gold nanoparticles (Au NPs) are successfully synthesized.

The property of the DNA-functionalized gold nanoparticles (Au NPs-DNA) is characterized by TEM, UV−*vis*, Zeta Potential and agarose gel. The Au NPs-DNA compared with the bare Au NPs (Figure S20C), the surface of Au NPs-DNA stained with phosphotungstic acid (3%) can be observed with a layer of halo (Figure S20D), suggesting that the DNA layer surrounded the Au NPs. As shown in Figure S20E, the maximum absorbance peak of UV−*vis* absorbance spectrum of Au NPs and Au NPs-DNA are 519 nm and 523 nm, respectively, suggesting the Au NPs and the Au NPs-DNA are successfully synthesized. Also, the zeta potential of Au NPs is also different from that of Au NPs-DNA in Figure S20F. Since DNA is negatively charged, the overall potential decreased after DNA modification on gold nanoparticles.


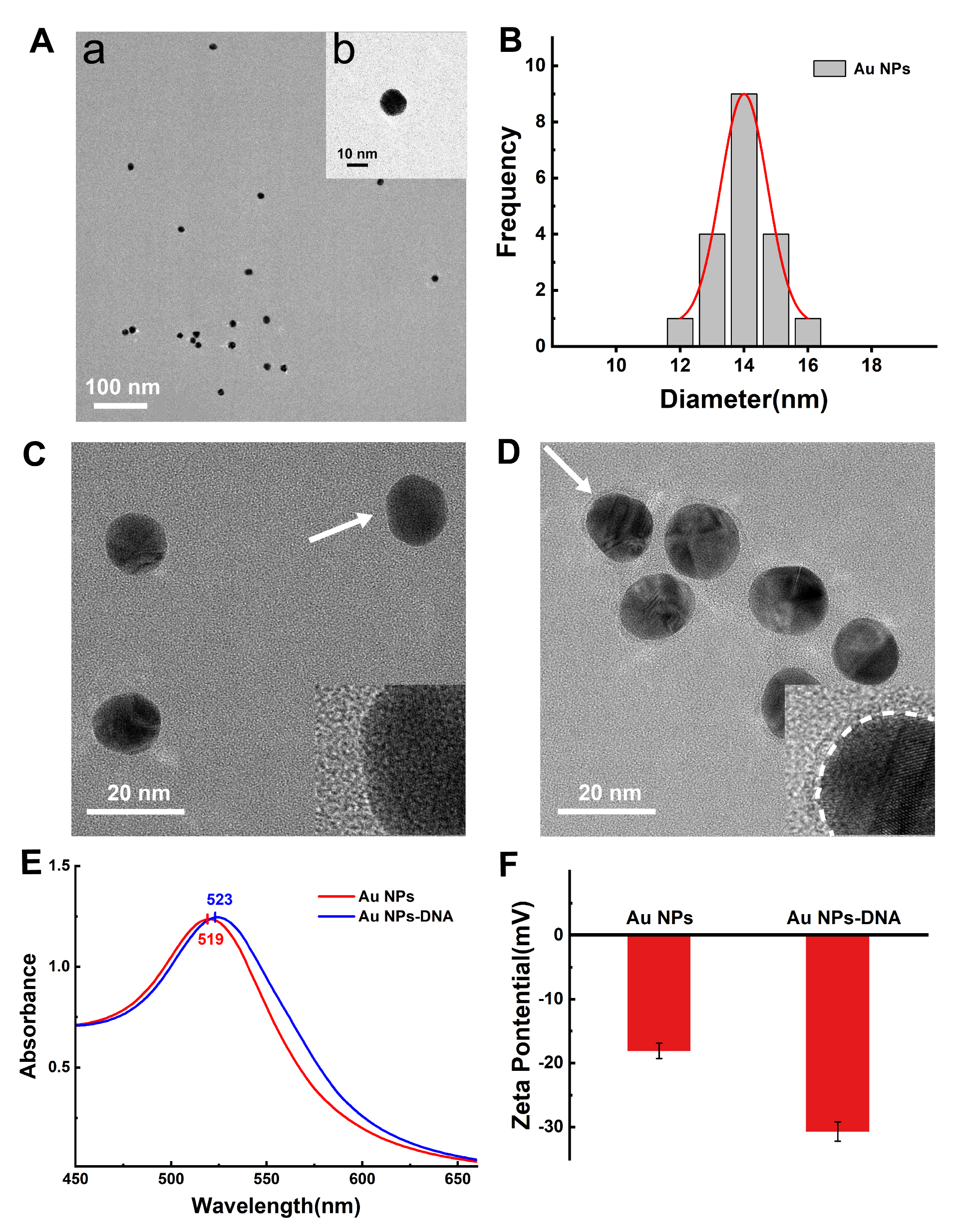


**Figure S20.** (A) TEM images of Au NPs, scale bar = 100 nm. The inset showed a single Au NP, scale bar = 10 nm. (B) The distribution of Au NPs diameter generated from Figure S20A. (C) Control TEM images of Au NPs without DNA functionalization, scale bar = 20 nm. (D) TEM images of DNA-functionalized Au NPs stained by phosphotungstic acid, scale bar = 20 nm. (E) UV−*vis* absorbance spectra of Au NPs (red line) and DNA-functionalized Au NPs (blue line). (F) Zeta potential of Au NPs and DNA-functionalized Au NPs.

Agarose gel electrophoresis of Au NPs and Au NPs-DNA is shown in Figure S21. lanes 1 corresponds to the 14 nm diameter Au NPs as the reference. Lane 2 and lane 3 corresponds to the previously modified DNA gold nanoparticle, Au NPs-S_6_ and Au NPs-S_5_, respectively. Because the DNA-functionalized gold nanoparticles increased the resistance through the gel pores during electrophoresis, the bands of lane 2 and lane 3 are slower than that of lane 1. This result proved the successful synthesis of DNA-functionalized gold nanoparticles.


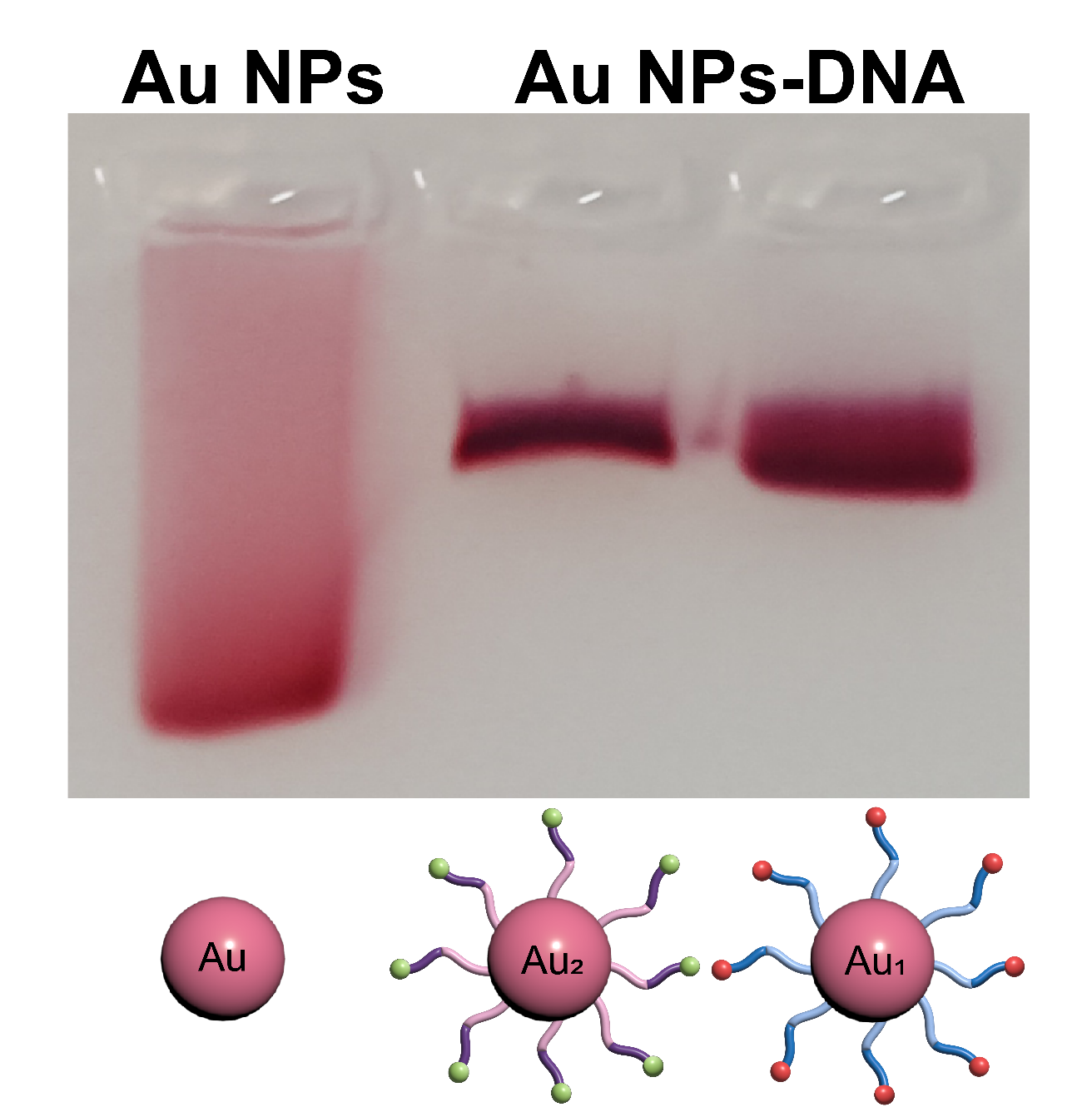


**Figure S21**. Agarose gel electrophoresis of Au NPs and Au NPs-DNA. Photograph is taken with a digital camera. Agarose (3%), gel thickness, 1 mm. All samples are separated upon applying a potential of 100 V, under ice bath. The separation of the samples is conducted for a time-interval of 0.5 hour in 0.5 × TBE. The 50% sucrose solution is added to the solution to obtain a final mixture of 25% sucrose to prevent the Au NPs and Au NPs-DNA from dispersing in the buffers during gel electrophoresis.

Note that Au NPs without a protective coating of nucleic acids are treated with BSPP solution to enhance their salinity tolerance.

**Quantification of DNA (S_5_ and S_6_) assembled on** **Au NPs**

To quantify the loading of S_5_ and S_6_ on the corresponding Au_1_ and Au_2_. DTT is added into solution of S_5_-functionalized Au_1_ and S_6_-functionalized Au_2_, respectively. The subjection of DTT leads to the release of S_5_/S_6_ from Au_1_ or Au_2_ due to reduction reaction of Au-S bond. According to the fluorescence intensity of the supernatant collected after DTT treatment (illustrated in Figure 22B and 22D) and the corresponding calibration curves of the fluorescence signal with different concentrations of S_5_/S_6_, Figure S22A and 22C, the number of S_5_ and S_6_ loaded on each gold nanoparticles is calculated to be ~ 240 and 220, respectively.

The concentration of S_5_ immobilized on the Au_1_ (5 nM) is approximately ~1.1 μM and the concentration of S_6_ immobilized on the Au_2_ (5 nM) is approximately ~ 1.2 μM. The calibration equation: *F* = -238.72514 + 2799.84269 × *C* (S_5_) and *F* = -290.20183 + 6592.21076 × *C* (S_6_), respectively.


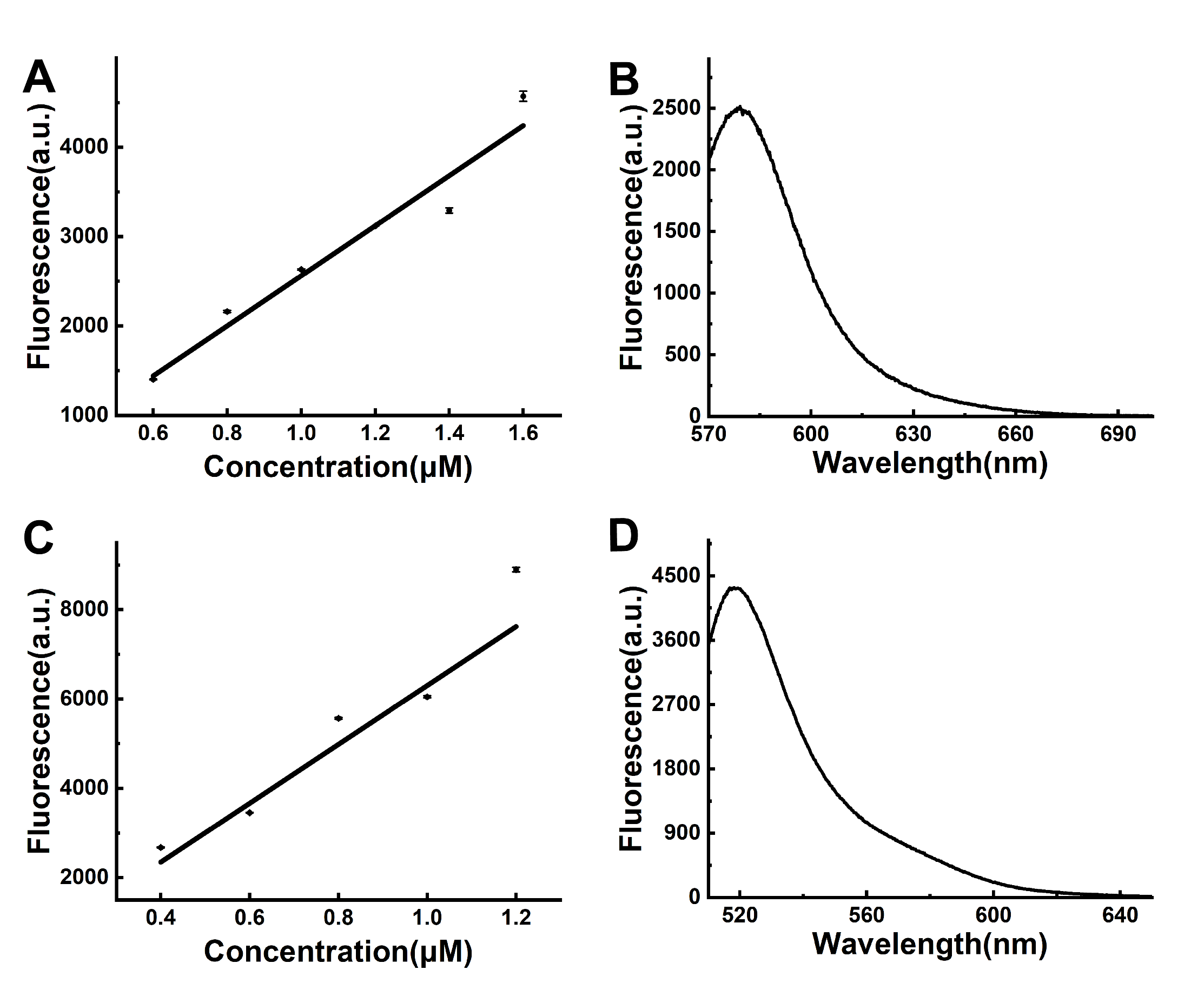


**Figure S22.** Quantification of DNA (S_5_ and S_6_) assembled on Au NPs. (A) Calibration curve of the fluorescence signal with different concentrations of S_5_. (B) Fluorescence spectrum of the supernatant after DTT treatment of S_5_-functionalized Au_1_ release of S_5_. The sample is excited at 546 nm (TAMRA) and collected the emission spectra from 570 to 700 nm. (C) Calibration curve of the fluorescence signal with different concentrations of S_6_. (D) Fluorescence spectrum of the supernatant after DTT treatment of S_6_-functionalized Au_2_ release of S_6_. The sample is excited at 546 nm (FAM) and collected the emission spectrum from 510 to 650 nm. Error bars represent mean ± SD, n = 3.

**Polyacrylamide gel electrophoresis (PAGE) characterization of the CDN system controlled over the orthogonal DNA molecular amplifiers**

To demonstrate the CDN system controlled over the orthogonal DNA molecular amplifiers, we used a classic native polyacrylamide gel electrophoresis (PAGE) experiment. The experimental results of the W_1_ cleaved sub5 system are shown in Figure S23A. Lanes 1-4 corresponds to ba', H_1_, S_1_ and S_2_, respectively. Taking S_1_ from lane 3 and S_2_ from lane 4 as the references, it can be concluded from lane 5 that S_1_ strand and S_2_ strand cannot directly form a duplex. Lane 6 corresponds to H_1-1_ strand, which is the key strand for the formation of W_1_. Lane 7 corresponds to H_1-1_, S_1_ strand and S_2_ strand. The higher band in lane 7 demonstrates the successful generation of W_1_. Lane 8 corresponds to the sample including ba', H_1_, S_1_, S_2_ and sub5. From the bands in lane 8, it can be concluded that the H_1-1_ derived from ba' cleaved-H_1_ self-assembled to S_1_ strand and S_2_ strand with the generation of W_1_. It should be noted that the band associated to W_1_ in lane 8 showed slightly growth, which may because the W_1_ and sub5 hybridized at low temperatures (in ice bath), and the W_1_ cleaved substrate (sub5) with the production of fragmented sub5 (marked with a dotted box). These results proved the successful engineering of the CDN constituents ba' guided the walker to digest a substrate through a hairpin structure. Accordingly, Figure S23B verified the CDN constituents aa' guided the walker to digest a substrate through a hairpin structure. According to the position of each set of lanes, these results confirmed the successful construction of the CDN system controlled over the orthogonal DNA molecular amplifiers.


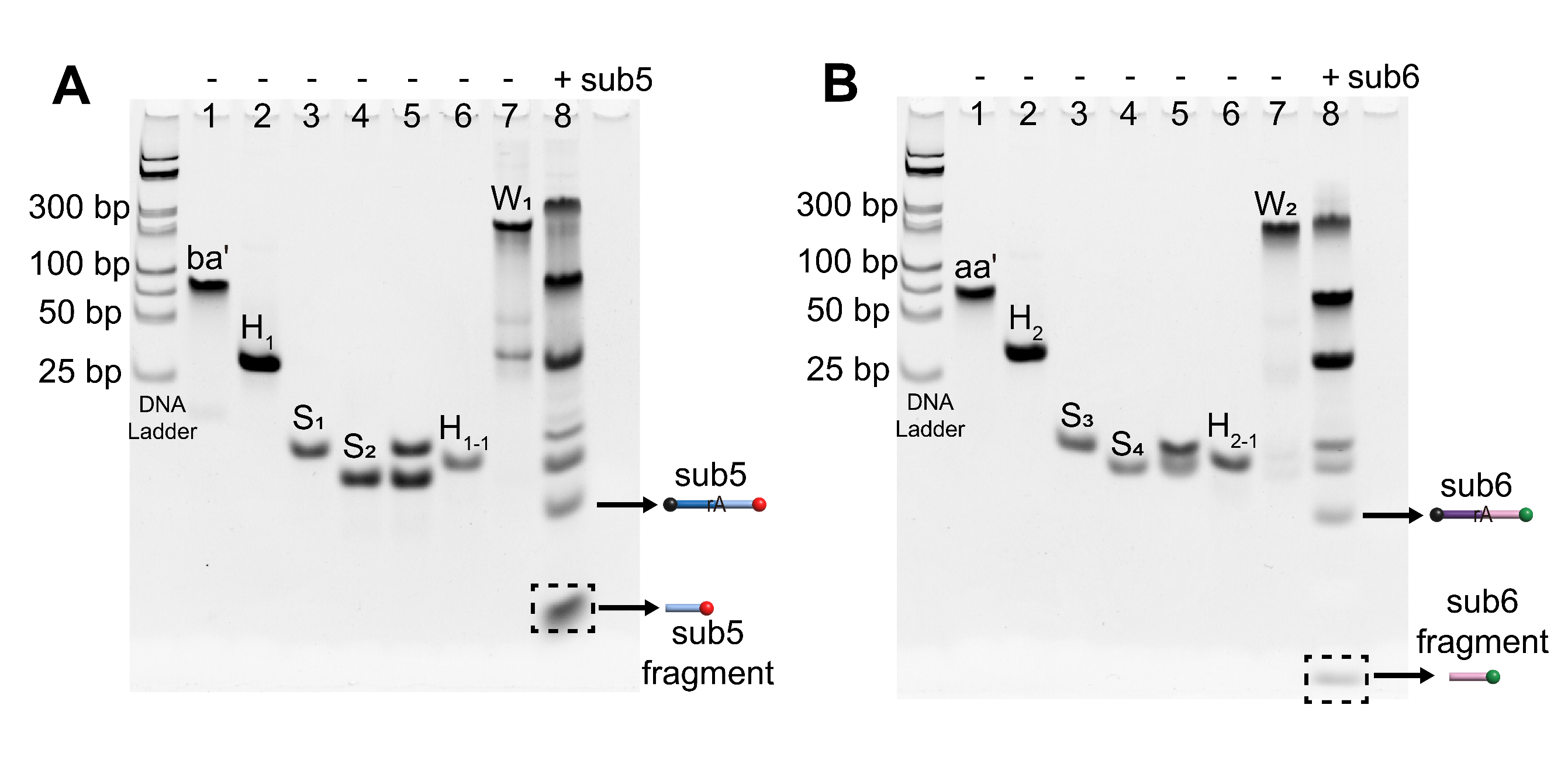


**Figure S23.** (A) Polyacrylamide gel electrophoresis (PAGE) characterization of CDN constituents ba' guided the walker to digest a substrate through a hairpin structure. DNA ladder: 25 bp - 500 bp; lane 1, ba'; lane 2, H_1_; lane 3, S_1_; lane 4, S_2_; lane 5, S_1_ + S_2_; lane 6, H_1-1_; lane 7, H_1-1_ + S_1_ + S_2_ (W_1_); lane 8, ba' + H_1_ + S_1_ + S_2_ +sub5. (B) Polyacrylamide gel electrophoresis (PAGE) characterization of CDN constituents aa' guided the walker to digest a substrate through a hairpin structure. DNA ladder: 25 bp - 500 bp; lane 1, aa'; lane 2, H_2_; lane 3, S_3_; lane 4, S_4_; lane 5, S_3_ + S_4_; lane 6, H_2-1_; lane 7, H_2-1_ + S_3_ + S_4_ (W_2_); lane 8, aa' + H_2_ + S_3_ + S_4_ +sub6. These samples concentration in each lane is 1 μM, excepted for the substrate (10 μM). Acrylamide (20%), gel thickness, 1 mm. All samples are separated upon applying the potential of 200 V, under ice bath. The separation of the samples is conducted for a time-interval of 106 min.

**UV-*vis* absorbance and fluorescence verification of the quenching effect of S_5_ (TAMRA) and S_6_ (FAM) by Au NPs**

To demonstrate the quenching effect of S_5_ (TAMRA) and S_6_ (FAM) by Au NPs, we used UV-*vis* absorbance and fluorescence experiments. Taking the quenching effect of S_5_ (TAMRA) by Au NPs as an example, Figure S24A showed the UV−*vis* absorbance spectrum of Au NPs and the fluorescence excitation spectrum of S_5_ (TAMRA), the quenching of TAMRA by Au NPs through the overlap between absorbance of the Au NPs and the fluorescent emission of TAMRA. The fluorescence emission spectrum of S_5_ (TAMRA) with a concentration of 1.1 μM is shown Figure S24B, orange curve. It is obvious that there is a strong fluorescence emission peak noted at 579 nm. However, when the S_5_ (TAMRA) is functionalized on Au NPs (The concentration of S_5_ (TAMRA) is kept at 1.1 μM), the emission peak at 579 nm decreased distinctly, as shown in Figure S24B, black curve. This experimental result indicates that the Au NPs can significantly quench the fluorescence of S_5_ (TAMRA). Accordingly, Figure S24C and S24D verified the quenching effect of S_6_ (FAM) by Au NPs.


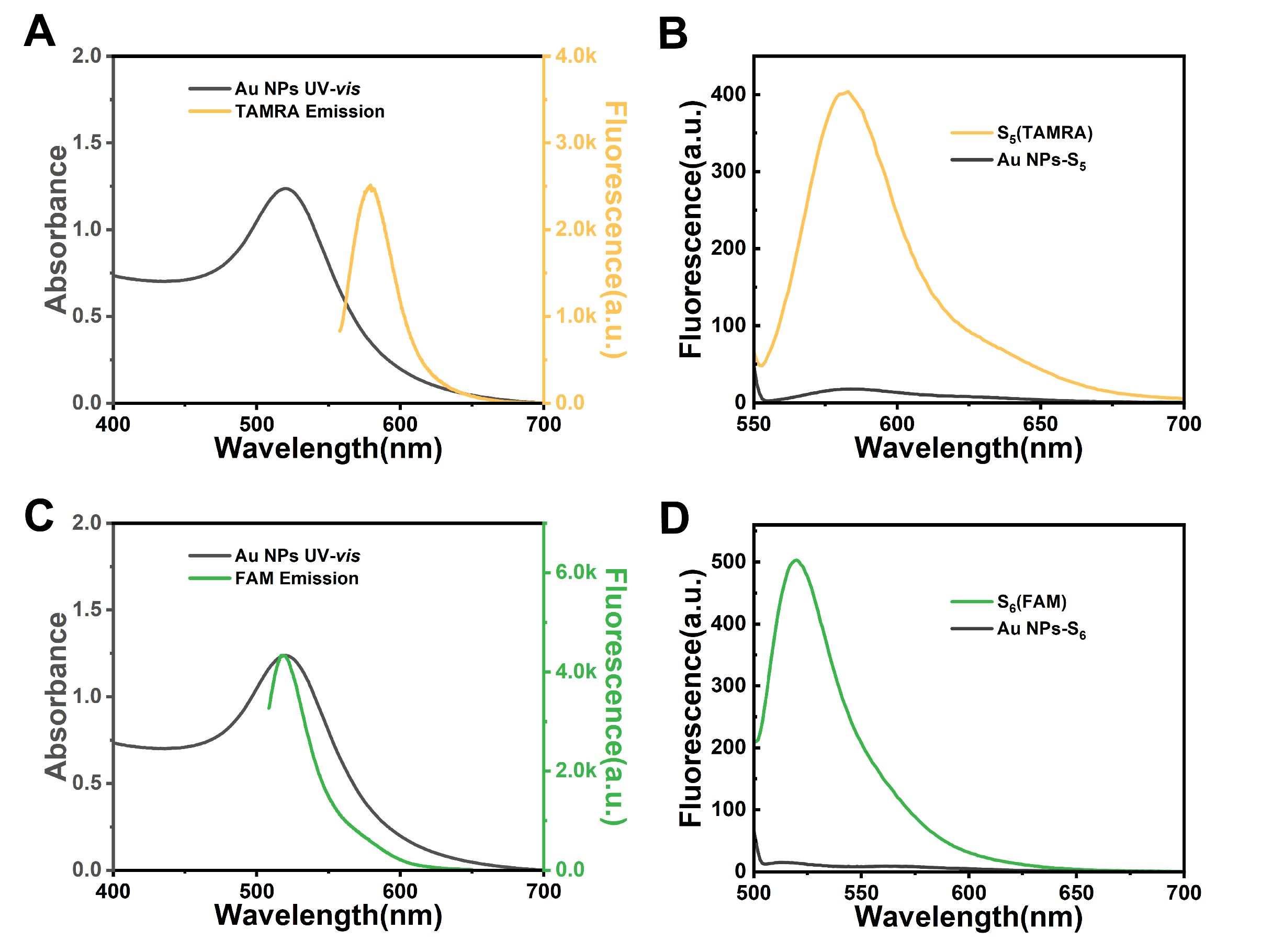


**Figure S24.** (A) The absorbance spectrum of the Au NPs and the fluorescent emission spectrum of TAMRA. (B) The TAMRA fluorescence emission spectrum of the free S_5_ strand and the S_5_ assembled on Au NPs. The concentration of S_5_ on Au NPs and in the homogeneous phase is the same, 1.1 μM. (C) The absorbance spectrum of the Au NPs and the fluorescent emission spectrum of FAM. (D) The FAM fluorescence emission spectrum of the free S_6_ strand and the S_6_ assembled on Au NPs. The concentration of S_6_ on Au NPs and in the homogeneous phase is the same, 1.2 μM.

**Time-dependent fluorescence verification of the formation of DNA walkers (W_1_ and W_2_)**

To demonstrate the formation of DNA walkers (W_1_ and W_2_), we used a H_1-1_-bridged S_1_/S_2_ to generate an intact DNAzyme structure that is able to cleave a corresponding substrate by recording the time-dependent fluorescence response. Taking the formation of W_1_ as an example, Figure S25A showed the time-dependent fluorescence of DNAzyme reporter units associated with W_1_ in the absence (black curve) and presence (blue curve) of H_1-1_, and the presence (red curve) of H_1_, respectively. Control experiments from curve black revealed that the S_1_ and S_2_ did not form into an intact DNAzyme reporter structure in the absence of H_1-1_. Also, hairpin H_1_ cannot be fully opened and stimulate the formation of W_1_ (red curve). While the time-dependent fluorescence response increased under the treatment of H_1-1_, demonstrating the efficient generation of the DNAzyme reporter by a cleaved hairpin (blue curve). These results originated from the fact that the hairpin H_1_ is engineered to be opened and stimulate the formation of W_1_ only in the presence of H_1-1_. Accordingly, the hairpin H_2_ is engineered to be opened and stimulate the formation of W_2_ only in the presence of H_2-1_, Figure S25B.


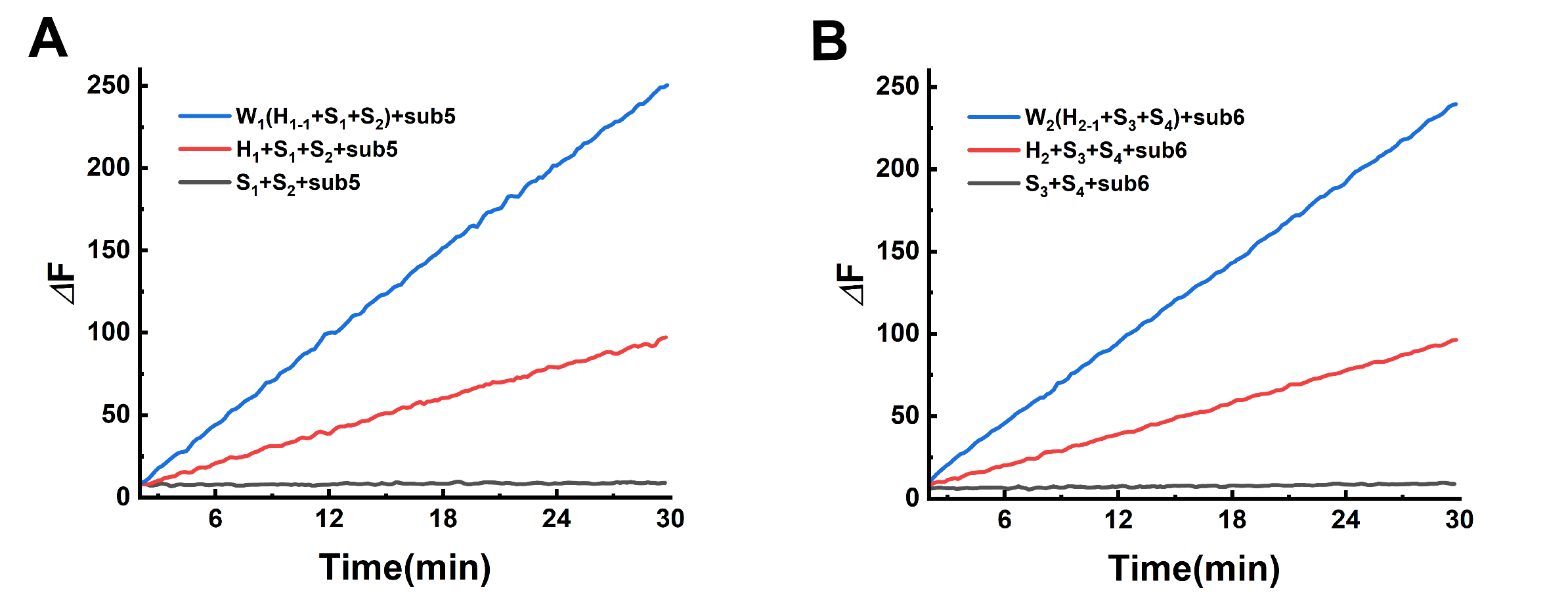


**Figure S25.** (A) Time-dependent fluorescence changes generated by DNAzyme reporter units associated with W_1_ in the absence (black curve) and presence (blue curve) of H_1-1_, and presence (red curve) of H_1_. (B) Time-dependent fluorescence changes generated by DNAzyme reporter units associated with W_2_ in the absence (black curve) and presence (blue curve) of H_2-1_, and presence (red curve) of H_2_.

**Preliminary experiments: Time-dependent fluorescence verification of the CDN system controlled over the orthogonal DNA molecular amplifiers** **in a homogeneous phase**


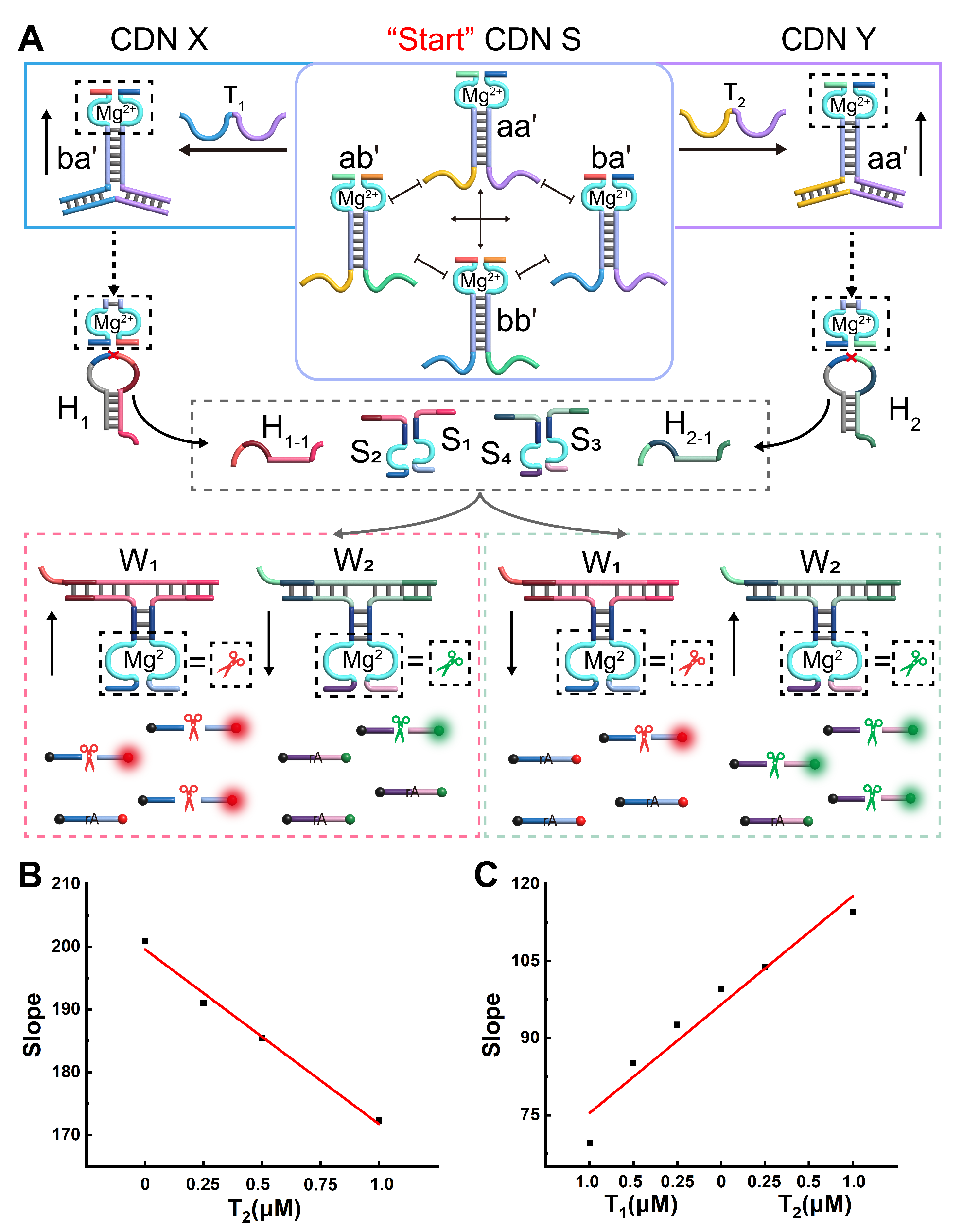


**Figure S26.** (A) Pre-experiments scheme: CDNs-controlled over the locomotion of orthogonal walkers by cleaving the sub5 and sub6. (B) The sensing curve of the slope of the W_1_ under different concentrations of T_2_. (C) The sensing curve of the slope of the W_2_ under different concentrations of T_1_ and T_2_.

**Time-dependent fluorescence characterization of the CDN system controlled over the orthogonal Au NPs-based DNA molecular amplifiers**

**
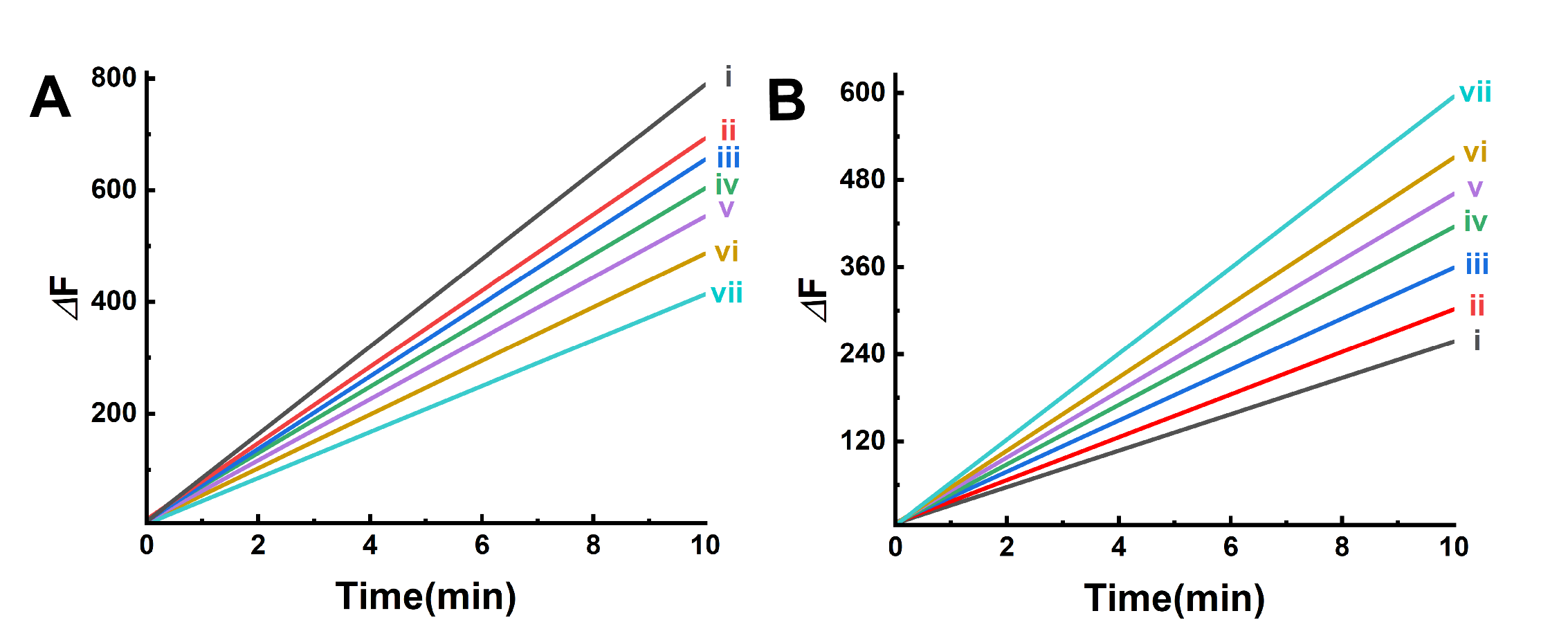
**

**Figure S27.** (A) Time-dependent fluorescence changes generated upon the cleavage of the S_5_-modified AuNPs by the W_1_ at different concentrations of trigger: (i) T_1_=1 μM, (ii) T_1_=0.5 μM, (iii) T_1_=0.25 μM, (iv) no T_1_ and T_2,_ (v) T_2_ =0.25 μM, (vi) T_2_ =0.5 μM, (vii) T_2_ =1 μM. (B) Time-dependent fluorescence changes generated upon the cleavage of the S_6_-modified Au NPs by the W_2_ at different trigger concentrations: (i) T_1_=1 μM, (ii) T_1_=0.5 μM, (iii) T_1_=0.25 μM, (iv) no T_1_ and T_2,_ (v) T_2_ =0.25 μM, (vi) T_2_ =0.5 μM, (vii) T_2_ =1 μM.

**A complicated text decrypted by hierarchically classifying the adaptive thresholds**

To enhance the length of the encrypted text, we employ a hierarchical classification of the adaptive threshold by transforming a single binary digit into a sequence of four binary digits, thereby significantly augmenting the scalability of the decrypted text. Taking Figure S28A as an example, the adaptive threshold exhibits a gradual increment in proportion to the concentration of T_1_. The adaptive threshold, derived by subtracting the average concentrations of aa' and bb' from the average concentrations of ab' and ba', is computed, and its absolute value is subsequently converted into a decimal format. This decimal value is then transformed into a binary representation in accordance with the methodology outlined in Figure S28C. The T_2_-triggered CDN-guided hierarchically thresholds are shown in Figure S28B.

The integration of inputs and outputs-I generates a 192-bit binary string, referred to as the ciphertext (as enumerated in Figure S29B(i)), by sequentially arranging the binary digits. The decryption process of the message, based on the 192-bit ciphertext, is governed by a transposition cipher protocol that rearranges the positions of the characters in the plaintext to produce the ciphertext. In this protocol, each 8-bit segment corresponds to a single letter, and the subsequent letter is represented by shifting one bit forward, as illustrated in Figure S29B(ii). For instance, the binary sequence “00010101” can be decoded as the letter “T”, while “00101010” corresponds to the character “H”. Utilizing this method, a 185-letter ciphertext composed of 8-bit segments is derived, along with its corresponding plaintext.

**
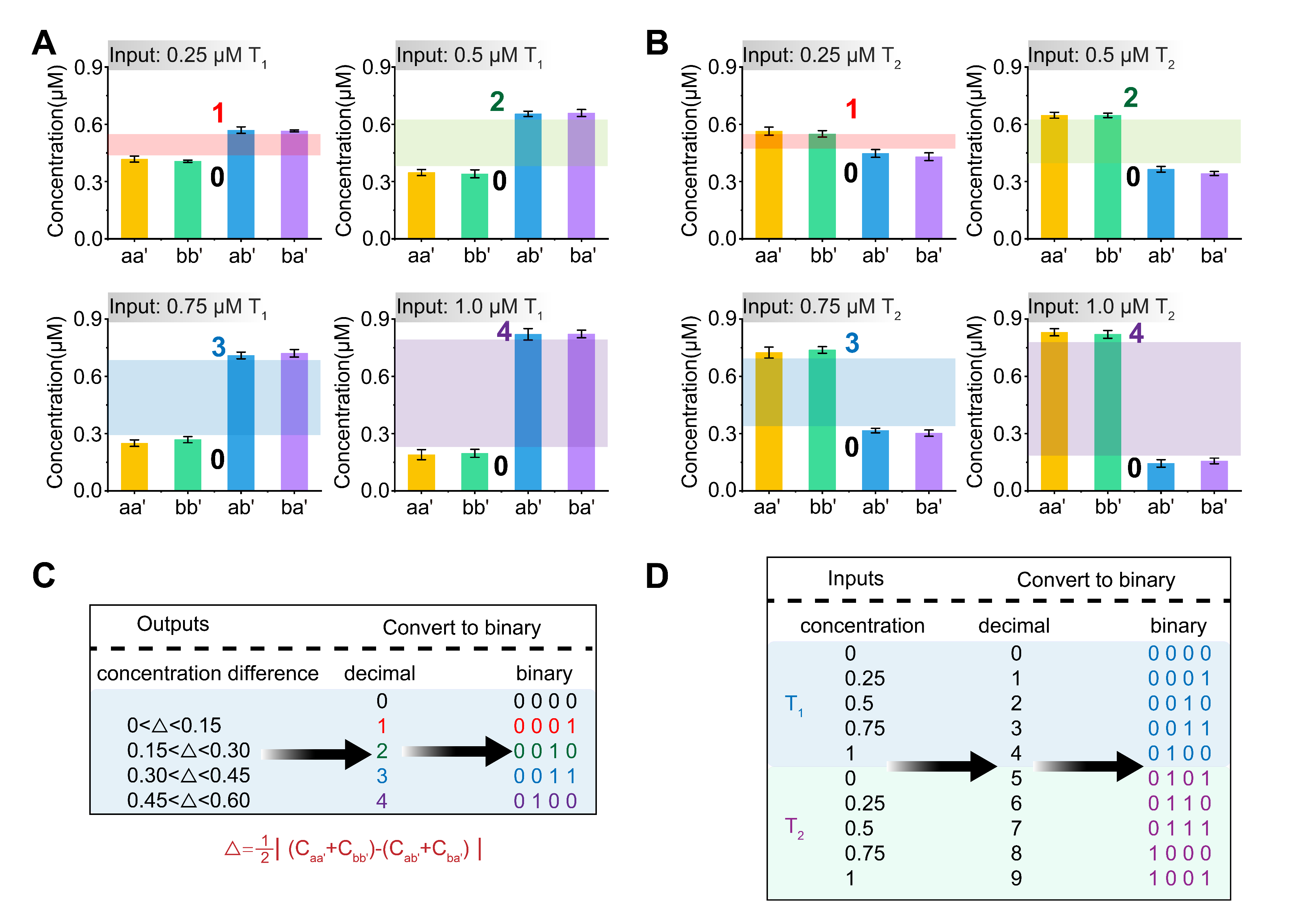
**

**Figure S28.** (A) Content changes generated by the reporter units associated with CDN constituents at different concentrations of T_1_ in the form of a bar presentation. The colored area is the hierarchically adaptive threshold. (B) Content changes generated by the reporter units associated with CDN constituents at different concentrations of T_2_ in the form of a bar presentation. The colored area is the hierarchically adaptive threshold. (C) The “outputs” library given by the “Sender” under specific concentrations difference of △ ((△= $\frac{\text{1}}{\text{2}}$｜(C_aa'_+C_bb'_) - (C_ab'_+C_ba'_)｜), and the corresponding decimal and binary digital translation. (D) The “inputs” library given by the “Sender” under specific concentrations of T_1_ and T_2_, and the corresponding decimal and binary digital translation.

**
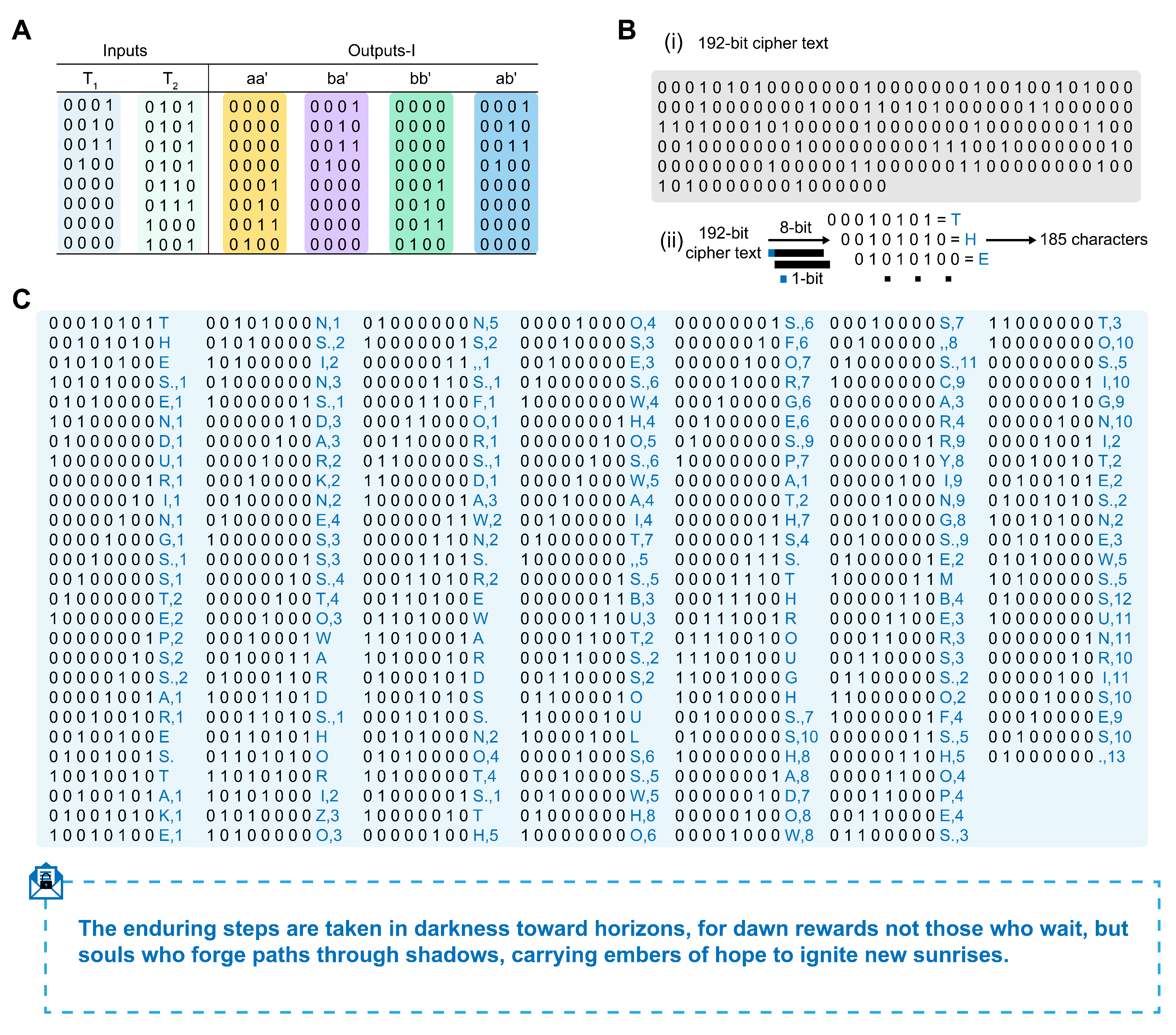
**

**Figure S29.** (A) The binary digits library of the inputs and outputs-I derived from Figure S28A and S28B after decimal and binary digits conversion. (B) (i) A 192-bit cipher text from the combination of inputs and outputs-I shown in (A). (ii) The schematic description of the transposition ciphers protocol that rearranges the positions of the characters in the plaintext to produce the ciphertext. (C) A 185-letter cipher book and the corresponding plain text. “S.” represents space. The notation “, number” following the letter signifies the sequential order in which the plaintext is presented.

**Quantification of DNA (****H_3_ and H_4_) assembled on Au NPs**

To quantify the loading of H_3_ and H_4_ on the corresponding Au_3_ and Au_4_. DTT is added into Au NPs-DNA solution of H_3_-functionalized Au_3_ and H_4_-functionalized Au_4_, respectively. The supernatant is collected after DTT treatment and purified for three times by ultrafiltration (Amicon® Ultra 3K, Millipore), and the excess DTT remaining in the solution is removed. After the purification of the reaction sample, the UV-*vis* absorbance is recorded as illustrated in Figure S30B. Based on the corresponding calibration curve of the UV-*vis* absorbance with different concentrations of H_3_, Figure S30A, the number of H_3_ loaded on Au_3_ is calculated to be ~ 124. Besides, the fluorescence intensity of the H_4_-functionalized Au_4_ supernatant collected after DTT treatment (illustration in Figure S30D), the number of H_4_ loaded on Au_4_ is calculated to be ~ 116.

The concentration of H_3_ immobilized on the Au_3_ (5 nM) is approximately ~ 0.62 μM according to the calibration equation: *Abs* = -0.1725 + 0.55962 × *C* (H_3_). The concentration of H_4_ immobilized on the Au_4_ (5 nM) is approximately ~ 0.58 μM according to the calibration equation: *F* = -256.74465 + 946.75303 × *C* (H_4_).


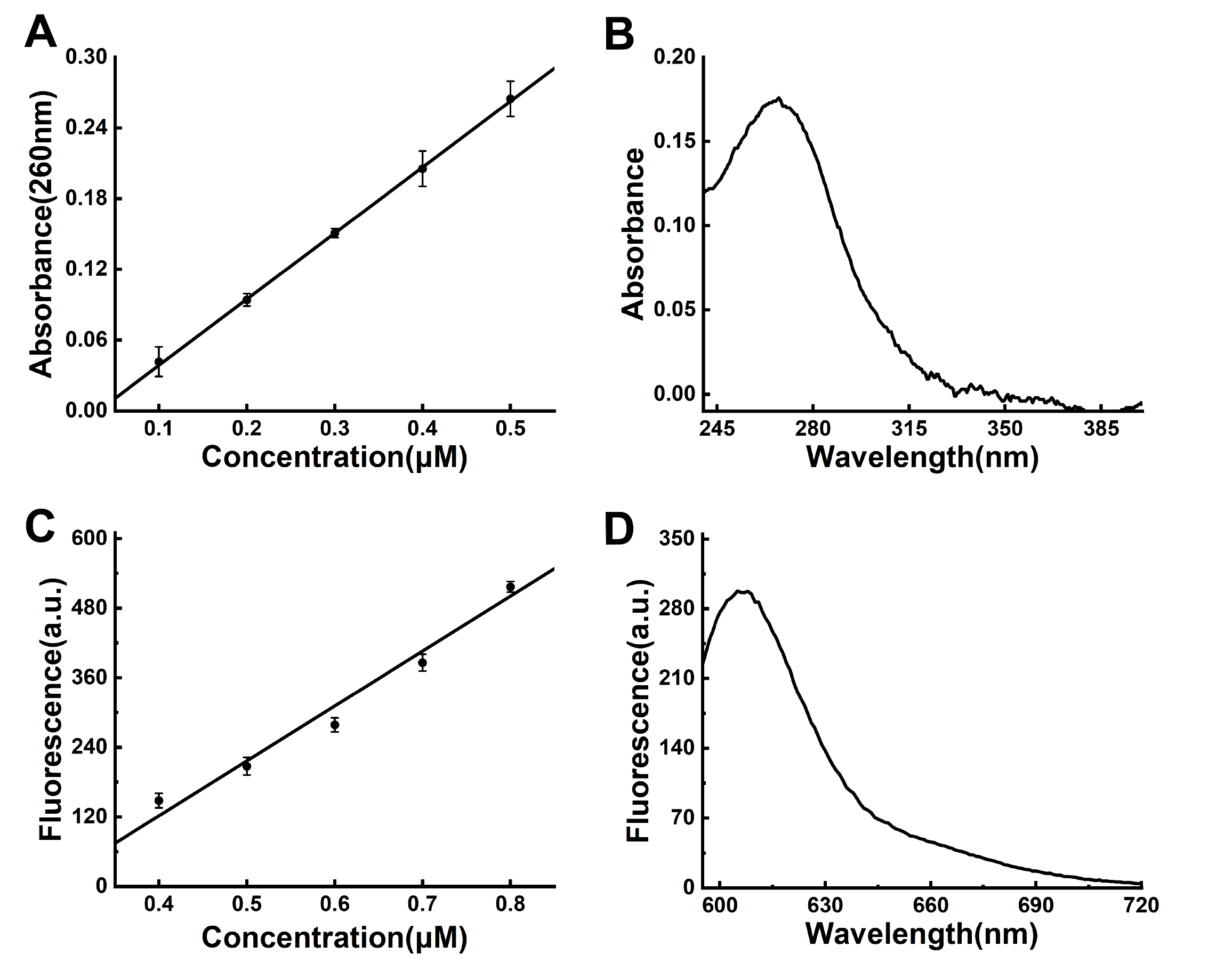


**Figure S30.** Quantification of DNA (H_3_ and H_4_) assembled on Au NPs. (A) Calibration curve of the UV-*vis* absorbance signal (260 nm) with different concentrations of H_3_. Error bars represent mean ± SD, n = 3. (B) UV-*vis* absorbance spectrum of the supernatant after DTT treatment of H_3_-functionalized Au_3_ release of H_3_. The sample is collected UV-*vis* absorbance spectrum at 260 nm. (C) Calibration curve of the fluorescence signal with different concentrations of H_4_. Error bars represent mean ± SD, n = 3. (D) Fluorescence spectra of the supernatant after H_4_-functionalized Au_4_ release of H_4_. The sample is excited at 583 nm (ROX) and collected the emission spectrum from 590 to 720 nm.

**UV-*vis* absorbance and fluorescence verification of the quenching effect of H_4_ (ROX) by Au NPs**

To demonstrate the quenching effect of H_4_ (ROX) by Au NPs, we used UV-*vis* absorbance and fluorescence experiments. Figure S31A showed the UV−*vis* absorbance spectrum of Au NPs and the fluorescence emission spectrum of H_4_ (ROX), the quenching of ROX by Au NPs through the overlap between absorbance of the Au NPs and the fluorescent emission of ROX. The fluorescence emission spectrum of H_4_ (ROX) with a concentration of 0.58 μM is shown Figure S31B, red curve. It is obvious that there is a strong fluorescence emission peak noted at 606 nm. However, when the H_4_ (ROX) is functionalized on Au NPs (the concentration of H_4_ (ROX) is kept at 0.58 μM), the emission peak at 606 nm decreases distinctly, as shown in Figure S31B, black curve. This experimental result indicates that the Au NPs can quench the fluorescence of H_4_ (ROX).

**
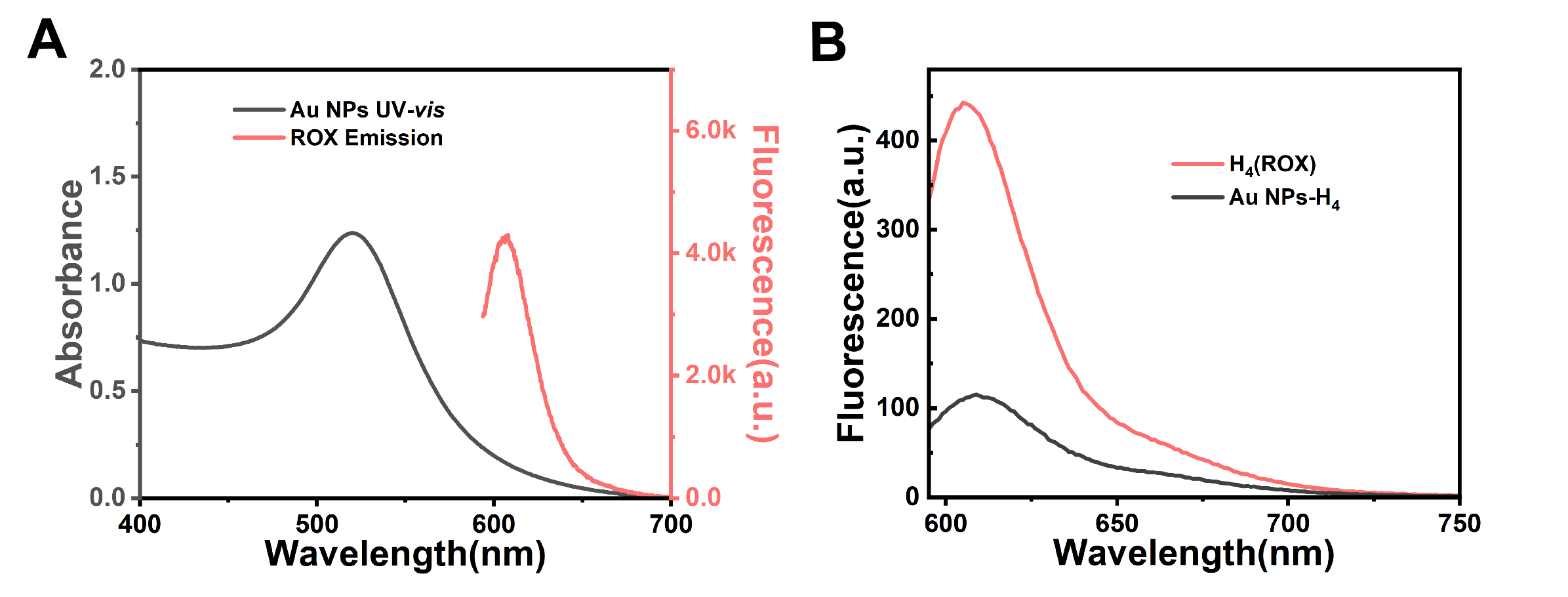
**

**Figure S31.** (A) The absorbance spectrum of the Au NPs and the fluorescent emission spectrum of ROX. (B) The ROX fluorescence emission spectrum of the free H_4_ strand and the H_4_ assembled on Au NPs. The H_4_ in a homogeneous phase at the same concentrations present on Au NPs, 0.58 μM.

**Preliminary experiments: Polyacrylamide gel electrophoresis (PAGE) and time-dependent fluorescence characterization of the aa'-regulated cascaded DNA molecular amplifiers**

As shown in Figure S32A, to demonstrate the CDN-controlled over the cascaded DNA molecular amplifiers, a fluorophore (ROX)/quencher (BHQ2)-modified on the two ends of H_4_ (FQ) is used to preliminarily cleaving of the Nt.BbvCI on the (1/1') domain on H_3_/ H_4_ duplex. The fluorescence of ROX is quenched when the H_4_ (FQ) stays the intact hairpin structure due to the close distance between the ROX and BHQ2. We used H_3-1_ to open the loop domain of H_4_, thus generating the nicking site of Nt.BbvCI, termed as the duplex, (1/1'). The cleavage of Nt.BbvCI on (1/1') is able to release the fluorophore-modified end of H_4_ (FQ) and to generate a fluorescence recovery of ROX due to the remoting from the quencher. Figure S32B, blue curve, showed the time-dependent fluorescence of the H_4_ (FQ) by subjecting the H_3-1_ and Nt.BbvCI. While in the absence of the H_3-1_, or substitution of H_3-1_ by an intact H_3_, the H_4_ (FQ) cannot hybridize to the (1) domain in H_3_ due to the lack of H_3_ or the block of (1') domain in the stem of H_3_. The time-dependent fluorescence of the H_4_ (FQ) corresponding to the above control experiments shows in Figure S32B, black curve and presence red curve. These results originated from the fact that the hairpin H_3_ is engineered to be opened and stimulate hairpin H_4_ (FQ) only in the presence of H_3-1_.

In addition to this, we also conducted a classical native polyacrylamide gel electrophoresis (PAGE) experiment. The experimental results are shown in Figure S32C. Lane 1-3 corresponded to aa', H_3_, and H_4_, respectively. Taking H_3_ from lane 2 and H_4_ from lane 3 as the references, it can be concluded from lane 4 that hairpin H_3_ and hairpin H_4_ cannot directly form a duplex. Lane 5 and lane 6 indicated that hairpin H_4_ or the mixture of H_3_/ H_4_ cannot be directly cleaved by endonuclease, Nt.BbvCI. Lane 7 corresponds to the sample including aa' and hairpin H_3_. From the bands in lane 7, it can be concluded that the H_3-1_ is derived from aa'-cleaved H_3_. Lanes 8 and 9 showed that H_3-1_ derived from aa' by cleaving hairpin H_3_, which can open hairpin H_4_, and cleavage of the (1/1') occurred only in the presence of Nt.BbvCI. The production fragments H_4-1_ is marked with a dotted box at lane 9. These results proved the successful engineering design of the CDN constituents aa'-guided the cascaded walker through a hairpin structure.

**
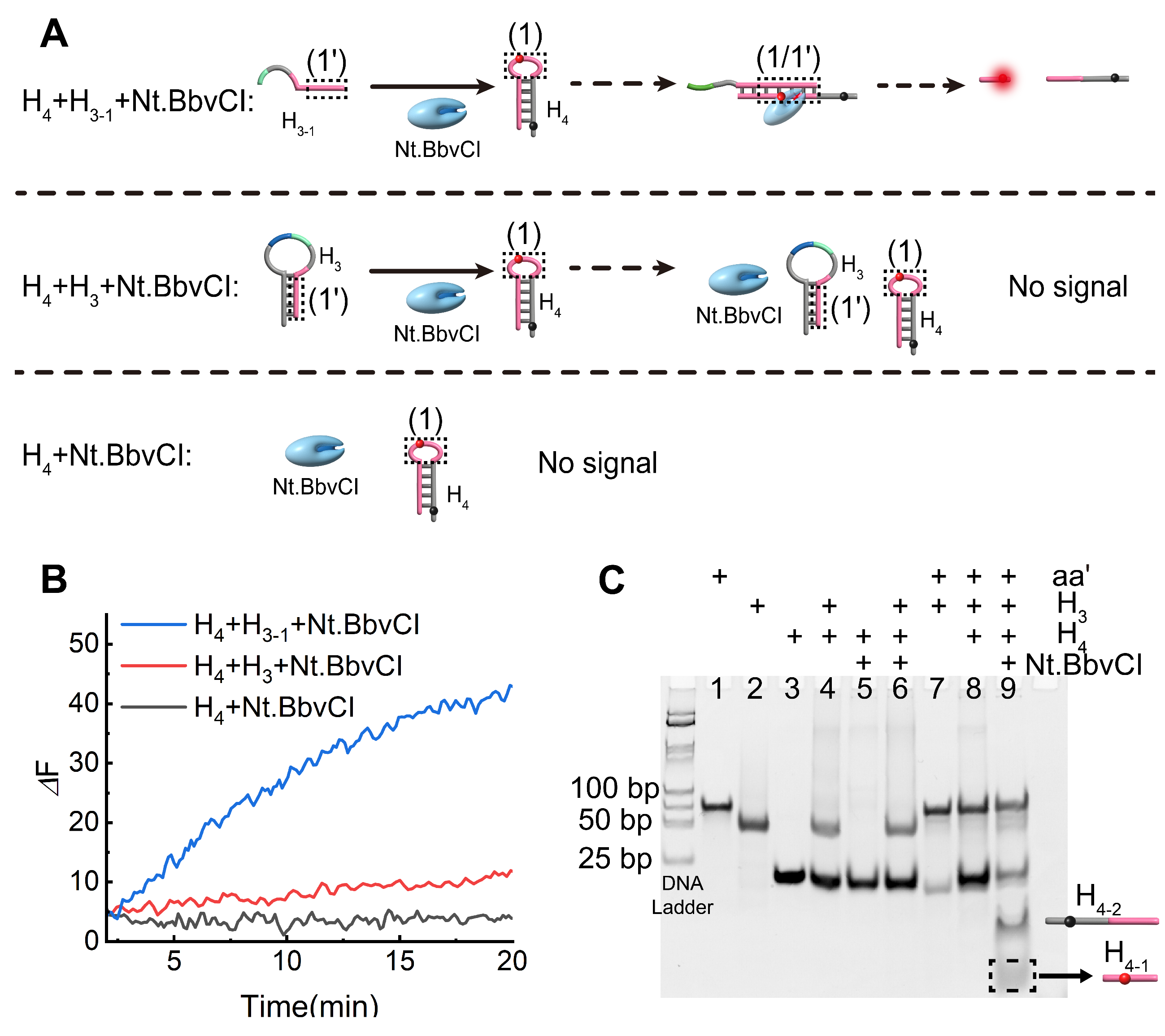
**

**Figure S32.** (A) Schematic presentation of the cascaded DNA walker by probing with a fluorophore/quencher-modified substrate, H_4_ (FQ) in a homogeneous phase. (B) Time-dependent fluorescence verification of the cascaded DNA walker (H_3-1_), the H_4_ (FQ) by subjecting the Nt.BbvCI (black curve), the H_4_ (FQ) by subjecting the H_3_ and Nt.BbvCI (red curve), and the H_4_ (FQ) by subjecting the H_3-1_ and Nt.BbvCI (blue curve). (C) Polyacrylamide gel electrophoresis (PAGE) characterization of aa'-regulated cascaded DNA molecular amplifiers. DNA ladder: 25 bp – 500 bp; lane 1, aa'; lane 2, H_3_; lane 3, H_4_; lane 4, H_3_ + H_4_; lane 5, H_4_ + Nt.BbvCI; lane 6, H_3_ + H_4_ + Nt.BbvCI; lane 7, aa' + H_3_; lane 8, aa' + H_3_ + H_4_; lane 9, aa' + H_3_ + H_4_+ Nt.BbvCI. The concentration of the samples in each lane is 1 μM. Acrylamide (20%), gel thickness, 1 mm. All samples are separated upon applying the potential of 200 V, under ice bath. The separation of the samples is conducted for a time-interval of 145 min.

**Preliminary experiments: Time-dependent fluorescence verification of the CDN-controlled over the cascaded DNA molecular amplifiers** **in a homogeneous phase**

The substrate, H_4_ (FQ), modified on the two ends with fluorophore ROX and quencher BHQ2 is used to substitute the H_4_ functionalized on the gold nanoparticles.


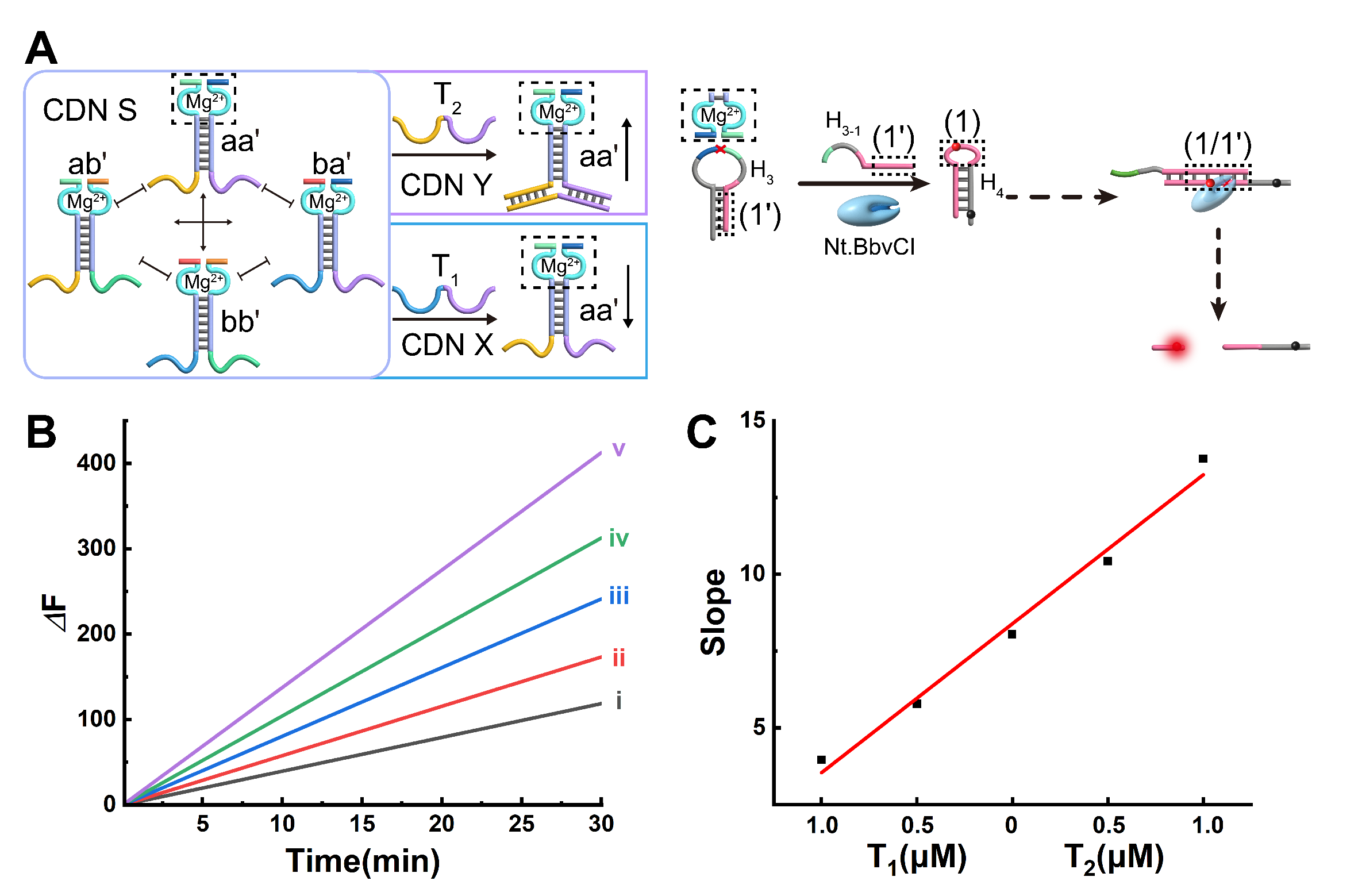


**Figure S33.** (A) schematic presentation of CDNs-controlled cascaded DNA walker by probing with a fluorophore/quencher-modified substrate, H_4_ (FQ) in a homogeneous phase. (B) Time-dependent fluorescence changes generated upon the cleavage of the H_4_ (FQ) by the H_3-1_ at different concentrations of T_1_ and T_2_: (i) T_1_=1 μM, (ii) T_1_=0.5 μM, (iii) no T_1_ and T_2,_ (iv) T_2_ =0.5 μM, (v) T_2_ =1.0 μM. (C) Corresponding sensing curves of the cleavage rates of the H_4_ (FQ) by the H_3-1_, derived from the data shown in Figure S33B.

**Control over the dissociation of** **aggregated Au NPs by CDNs**

We apply the CDNs to control the dissociation of aggregated Au NPs and to control the resulting optical property of the Au NPs by the respective dissociates. A schematic representation of the cross-talk between Au_3_ and Au_4_ is provided in Figure S34. Figure S34A shows the mechanism to induce the aggregation of Au NPs by annealing the mixture of Au_3_ and Au_4_ under the same concentrations. Figure S34B represents the mechanism of the dissociation of aggregated Au NPs by CDNs. The CDNs are subjected to aggregated Au NPs, the duplex domain (1/1') engineered in H_3_/ H_4_ acting as the substrate for the Mg^2+^-ion-dependent DNAzyme associated with the constituent aa'. The aa' cleaved the ribonucleobase-modified loop domain of H_3_, the cleavage of the H_3_ induces, then, the dissociation of aggregated Au NPs. The dissociation of aggregated Au NPs regulated by CDN “X”, “S”, “Y” is probed by TEM images, and the dissociation of aggregated Au NPs regulated by CDN “X”, “S”, “Y” is probed by UV-*vis* absorbance spectra.


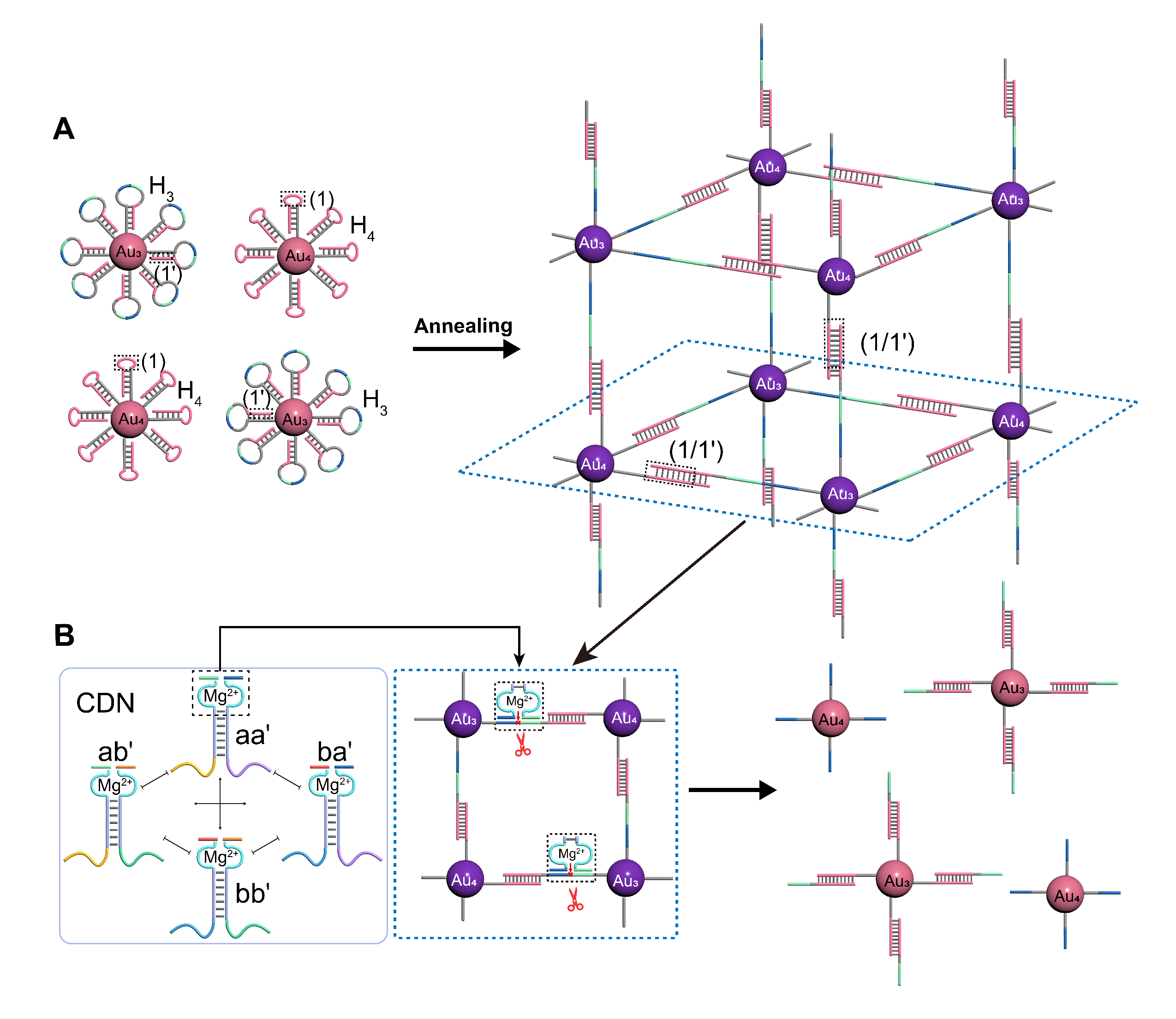


**Figure S34.** Scheme of the assembly and disassembly of DNA-functionalized Au NPs. (A) Scheme of formation of DNA-cross-linked Au NPs aggregates. (B) Scheme of CDNs controlling the dissociation of Au NPs aggregates.


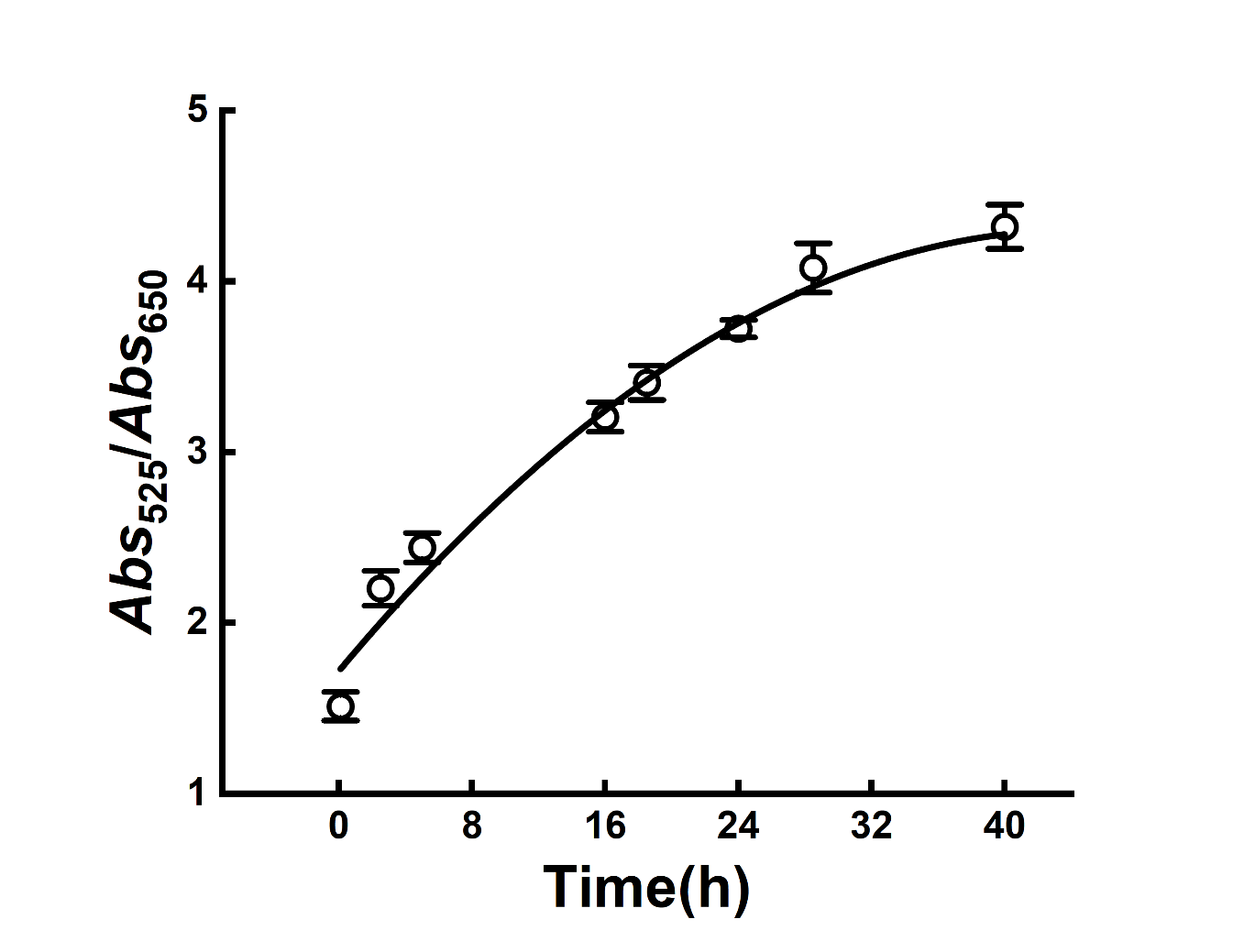


**Figure S35.** Time-dependent absorbance ratio changes of 525 nm and 625 nm upon the dissociation of the Au NPs generated by the equilibrated CDN “X”. Data from Figure S36A.

**
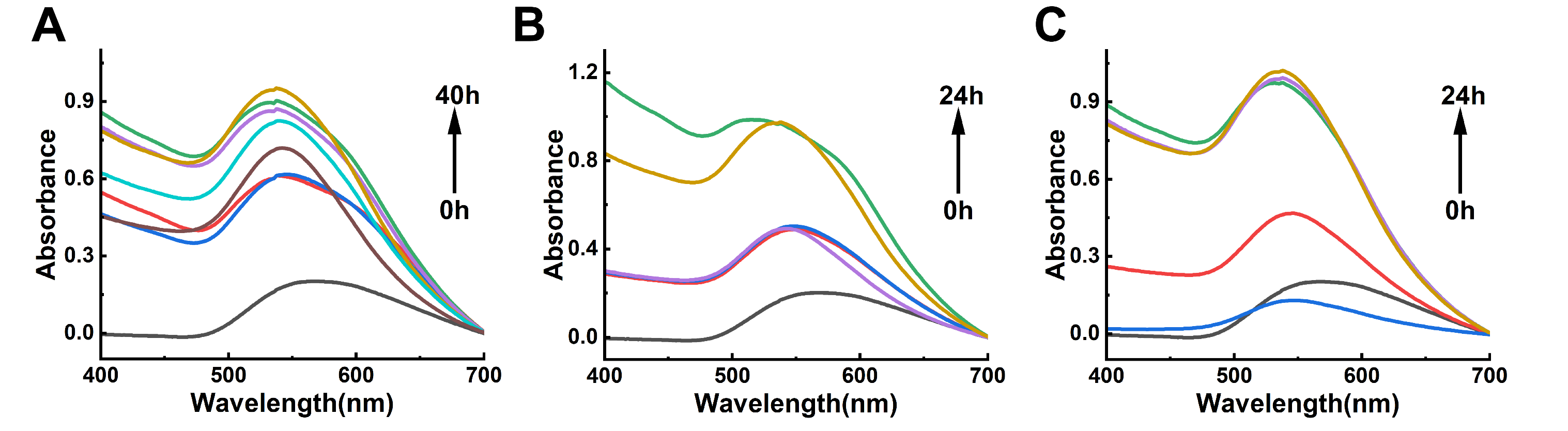
**

**Figure S36.** (A) Absorbance spectra associated with the time-dependent dissociation of the Au NPs in the presence of the equilibrated CDN “X”. (B) Absorbance spectra associated with the time-dependent dissociation of the Au NPs in the presence of the equilibrated CDN “S”. (C) Absorbance spectra associated with the time-dependent dissociation of the Au NPs that are subjected to the CDN “Y”.

**Binary translation based on individual ba'-based constituent**

**
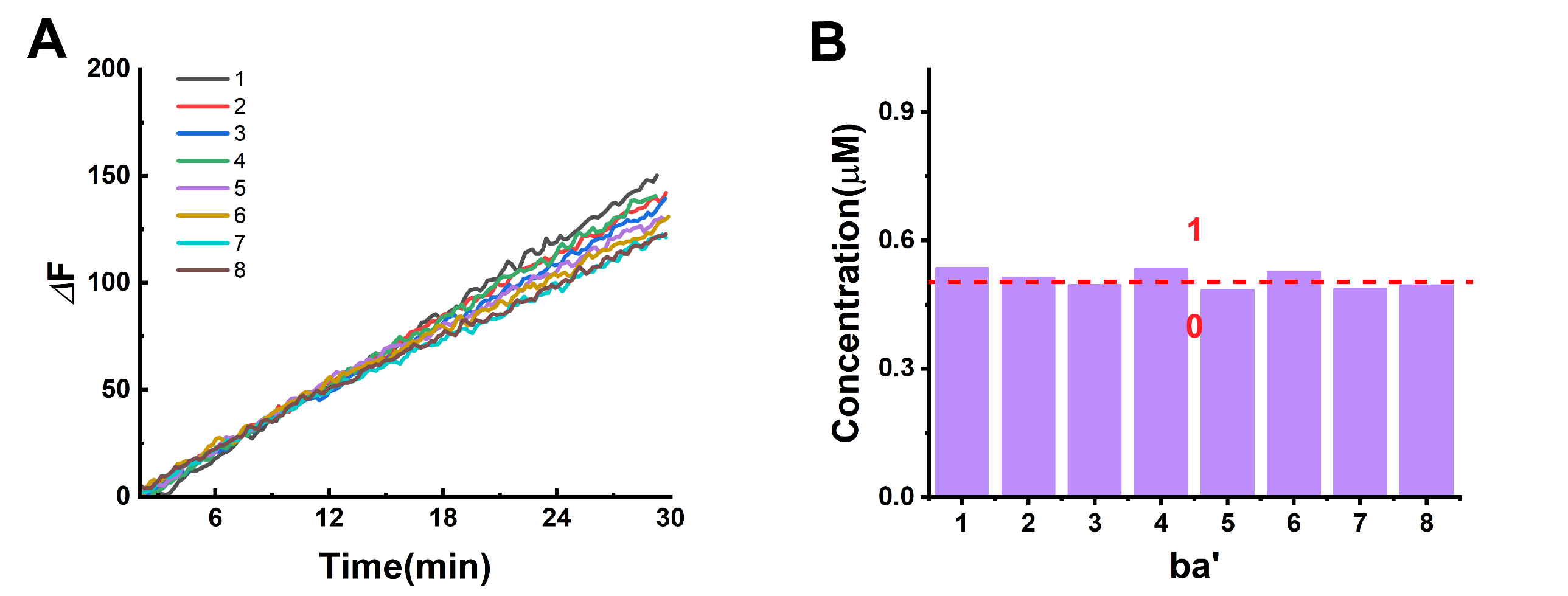
**

**Figure S37.** (A) Time-dependent fluorescence changes generated by DNAzyme reporter unit associated with constituent of the ba' under 8 parallel measurements. (B) The concentrations of ba' in the form of a bar presentation. The concentrations of the ba' are determined by the time-dependent fluorescence changes generated by the DNAzyme reporter unit and using appropriate calibration curve in Figures S5. The red dotted line is the fixed threshold.

**Binary translation based on individual bb'-based constituent**

**
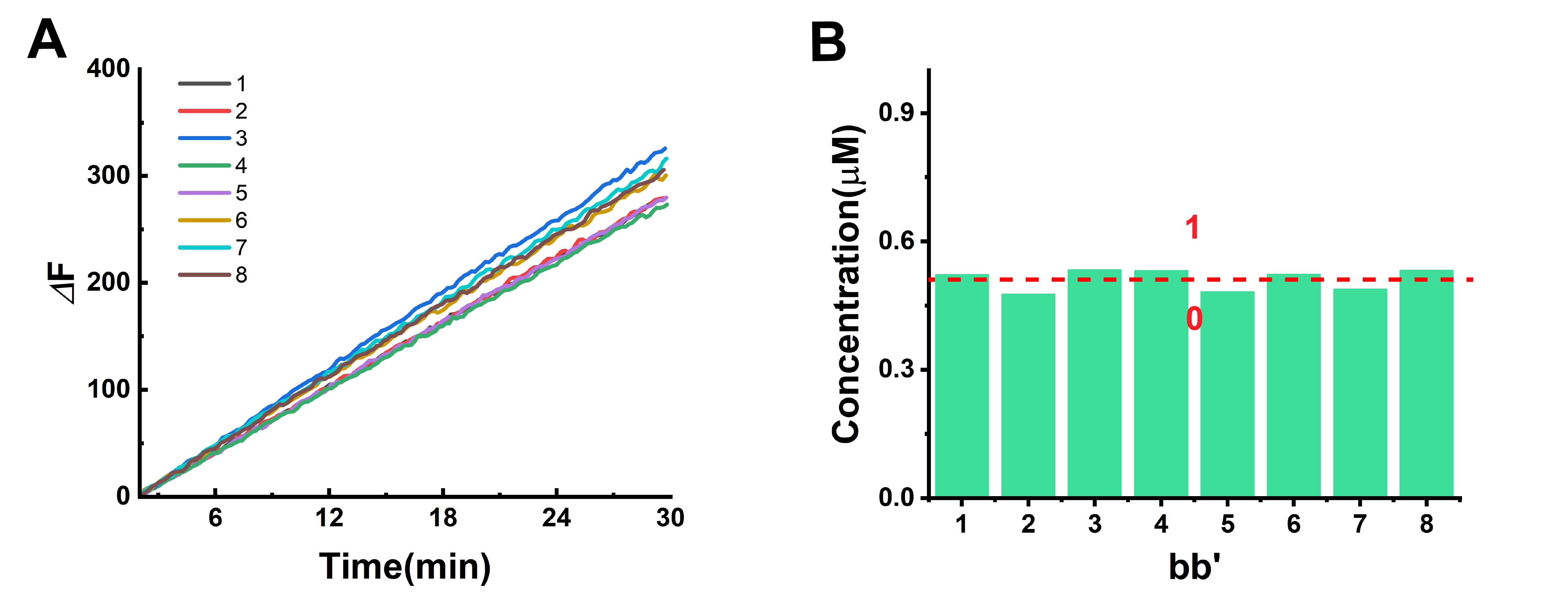
**

**Figure S38.** (A) Time-dependent fluorescence changes generated by DNAzyme reporter unit associated with constituent of the bb' under 8 parallel experiments. (B) The concentrations of bb' in the form of a bar presentation. The concentrations of the bb' are determined by the time-dependent fluorescence changes generated by the DNAzyme reporter unit and using appropriate calibration curve in Figures S5. The red dotted line is the fixed threshold.

**REFERENCES**

[1] J. Zhang, S. Song, L. Wang, D. Pan, C. Fan, *Nat. Protoc.* **2007**, *2*, 2888.

[2] J. Liu, Y. Lu, *Nat. Protoc.* **2006**, *1*, 246.

[3] M. Wei, N. Chen, J. Li, M. Yin, L. Liang, Y. He, H. Song, C. Fan, Q. Huang, *Angew. Chem. Int. Ed.* **2012**, *51*, 1202.

[4] D. Lim, K. Jeon, J. Hwang, H. Kim, S. Kwon, Y. D. Suh, J. Nam, *Nat. Nanotechnol.* **2011**, *6*, 452.
